# The Origin and Diversity of *Cpt1* Genes in Vertebrate Species

**DOI:** 10.1371/journal.pone.0138447

**Published:** 2015-09-30

**Authors:** Mónica Lopes-Marques, Inês L. S. Delgado, Raquel Ruivo, Yan Torres, Sri Bhashyam Sainath, Eduardo Rocha, Isabel Cunha, Miguel M. Santos, L. Filipe C. Castro

**Affiliations:** 1 CIIMAR, Interdisciplinary Centre of Marine and Environmental Research, CIMAR Associate Laboratory, UPorto–University of Porto, Porto, Portugal; 2 ICBAS, Abel Salazar Biomedical Sciences Institute, University of Porto, Porto, Portugal; 3 Department of Biology, Faculty of Sciences, University of Porto, Porto, Portugal; University of Rouen, France, FRANCE

## Abstract

The *Carnitine palmitoyltransferase I* (*Cpt1*) gene family plays a crucial role in energy homeostasis since it is required for the occurrence of fatty acid β-oxidation in the mitochondria. The exact gene repertoire in different vertebrate lineages is variable. Presently, four genes are documented: *Cpt1a*, also known as *Cpt1a1*, *Cpt1a2*; *Cpt1b* and *Cpt1c*. The later is considered a mammalian innovation resulting from a gene duplication event in the ancestor of mammals, after the divergence of sauropsids. In contrast, *Cpt1a2* has been found exclusively in teleosts. Here, we reassess the overall evolutionary relationships of *Cpt1* genes using a combination of approaches, including the survey of the gene repertoire in basal gnathostome lineages. Through molecular phylogenetics and synteny studies, we find that *Cpt1c* is most likely a rapidly evolving orthologue of *Cpt1a2*. Thus, *Cpt1c* is present in other lineages such as cartilaginous fish, reptiles, amphibians and the coelacanth. We show that genome duplications (2R) and variable rates of sequence evolution contribute to the history of *Cpt1* genes in vertebrates. Finally, we propose that loss of *Cpt1b* is the likely cause for the unusual energy metabolism of elasmobranch.

## Introduction

Long chain fatty acids are vital players in energy homeostasis since they undergo catabolism through the β-oxidation pathway in the mitochondria. Given that the inner mitochondrial membrane is only permeable to acyl groups if linked to carnitine, fatty acid uptake requires the action of carnitine palmitoyltransferase (CPTs). This system comprises two proteins with reverse functions, CPT1 and CPT2, residing in the outer and inner mitochondrial membranes, respectively [1]. CPT1 is the rate-limiting enzyme in the trans-esterification of acyl groups from coenzyme A (CoA) to carnitine due to its sensitivity and inhibition by malonyl-CoA, an intermediate of fatty acid synthesis [2].

In mammals CPT1 enzymes are encoded by three separate genes designated *Cpt1a*, *Cpt1b* and *Cpt1c*, each expressed in different tissue compartments [1, 3–5]. *Cpt1a*, known as the liver-expressing enzyme, is found also in other tissues [6]. The second isoform, *Cpt1b*, is primarily expressed in cardiac and skeletal muscle, hence termed muscle specific, although it can also be detected in testis and adipose tissue [1, 3, 5]. A more divergent *Cpt1* gene was described and named *Cpt1c*. Commonly designated as the brain isoform, it is expressed mostly in the hypothalamus but residual levels can also appear in the ovary, testis and intestine [1, 7].

The evolution and orthology assignment of vertebrate *Cpt1* genes has posed complex questions. Orthologs of *Cpt1a*, also referred as *Cpt1a1* (see below), and *Cpt1b* have been previously identified in most vertebrate lineages [5, 8–11]. As for *Cpt1c*, the origin and function has remained difficult to elucidate. The prevailing consensus considers that *Cpt1c* is a recent gene duplicate that emerged in the mammalian lineage [2, 5, 12], probably acting as a malonyl-CoA targeted energy-sensor [2]. Recently, Ka and collaborators (2013) suggested that the sauropsid *Cpt1b* is pro-orthologous to mammalian *Cpt1b* and *Cpt1c* [8].

A further line of evolutionary complexity results from the identification of two extra *Cpt1* genes designated *Cpt1a2* alpha and beta, so far identified uniquely in teleosts [5, 9]. Their phylogenetic positioning suggests that they are a subfamily of *Cpt1a* [5, 9].

Here we re-examine the repertoire and evolutionary history of *Cpt1* genes in vertebrate species. By means of comparative genomics, phylogenetics and sampling of a basal vertebrate lineage, the chondrichthyans, we provide important insights into the evolution of the *Cpt1* gene family.

## Material and Methods

### Database identification and collection of Cpt1 genes

Using the *H*. *sapiens* CPT amino acid sequences, blastp and tblastn searches were performed in NCBI and Ensembl databases in order to identify and retrieve sequences from the following species: *Mus musculus*, *Sus scrofa*, *Monodelphis domestica*, *Gallus gallus*, *Falco peregrinus*, *Anolis carolinensis*, *Xenopus tropicalis*, *Latimeria chalumnae*, *Danio rerio*, *Takifugu rubripes*, *Oryzias latipes*, *Gasterosteus aculeatus*, *Oreochromis niloticus*, *Tetraodon nigroviridis*, *Tachysurus fulvidraco*, *Callorhynchus milii*, *Drosophila melanogaster* and *Ciona instestinalis*. For *Leucoraja erinacea* Blast searches were performed on the existing genome assemblies (Build 2) and transcriptomic assemblies (Build 2) available at SkateBase [13] (S1 Table).

### Phylogenetic analysis

Sequence alignment was performed using MAFFT with the L-INS-i method [14]. The final alignment with 52 sequences was curated in BioEdit version 7.2.5 [15] with the removal of all columns containing gaps (S1 Fig), leaving an alignment with 655 gaps free sites for phylogenetic analysis. The original file with the sequence alignment containing gaps was also maintained for further phylogenetic analysis (S2 and S3A Figs). To determine the best evolutionary model of amino acid substitution, the sequence alignments were submitted to the ProtTest 2.4 server, resulting in a LG+I+G+F model [16]. Maximum Likelihood trees were reconstructed using PhyML 3.0 [17]. Branch support was assessed with aBayes [18]. Supplementary phylogenetic analysis using bayesian inference and neighbor-joining methods were conducted with the initial sequence alignment without gaps. Methods are described in S3B, S3C and S3D Fig. The resulting trees were visualized in Fig Tree V1.3.1 and rooted with the *Cpt1* homologues of *D*. *melanogaster* and *C*. *intestinalis*.

### Comparative genomics and neighbouring gene families

Comparative synteny maps were constructed with Ensembl comparative genomics pipeline, using as reference the latest available genome assemblies (Ensembl release 80—May 2015) for the following species: *H*. *sapiens* (GRCh38.p2), *M*. *domestica* (monDom5), *G*. *gallus* (Galgal4), *A*. *carolinensis* (AnoCar2.0), *X*. *tropicalis* (JGI_4.2), *L*. *chalumnae* (LatCha1) and *D*. *rerio* (GRCz10). The *F*. *peregrinus* data was collected from the latest assembly *F*. *peregrinus* v1.0 available in NCBI. For each species we analysed the genomic location of each *Cpt* gene, as well as, the five contiguous flanking genes to each side of the target gene, when possible. Following the assembly of the synteny maps, we proceeded to identify and localize the corresponding human orthologues of non-conserved neighbouring genes. Orthology was determined through the Ensembl orthologue-paralogue pipeline and our own phylogenetic analysis (not shown). Finally, synteny maps and annotated orthologues were then used to infer the localization of the ancestral *Cpt* gene in the reconstructed genome of the vertebrate ancestor using as reference the reconstruction presented by Nakatani and colleagues [19]. Synteny statistics was performed using CHSminer v1.1 [20]; input data was automatically retrieved from ensemble release 64, statistical analysis was performed for *H*. *sapiens* vs *A*. *carolinesis* and *H*. *sapiens* vs *D*. *rerio* and *H*. *sapiens* vs *X*. *tropicalis* when possible. If not indicated otherwise search parameters maintained as default maximal gap = < 30 and size> = 2. To further support synteny analysis we selected two flanking genes from each *Cpt1 locus* with representation in the majority of lineages analysed if not all, and performed phylogenetic analysis to address the orthology of the sequences (methods described in S4, S5 and S6 Figs).

### Polymerase chain reaction (PCR) and gene expression analysis

Tissues were collected from *Leucoraja erinacea* obtained from Woods Hole, USA (kind gift from Neelakanteswar Aluru). Procedures were approved by the Animal Care and Use Committee of the Woods Hole Oceanographic Institution. Total RNA was extracted using the illustra RNAspin Mini kit (GE Healthcare, UK). The RNA extraction process included an on-column DNase I treatment (provided in the kit). RNA integrity was assessed on a 1% agarose TAE gel stained with GelRed™ nucleic acid stain (Biotium, Hayward, CA, USA). The Quant-iT™ RiboGreen® RNA Assay Kit (Life Technologies, Carlsbad, CA, USA) was used to measure total RNA concentration. Reverse transcription reactions were performed with the iScript cDNA Synthesis Kit (Bio-Rad, Hercules, CA, USA). Primers targeting *Cpt1a* and *Cpt1c* genes were designed with Primer3 Software [21], using the unassembled genome sequence from *L*. *erinacea* [13]. PCR was performed with Phusion Flash High-Fidelity PCR Master Mix (Thermo Fisher Scientific, USA). Reactions were set up for a final volume of 20 μl, sense and anti-sense primer concentrations of 500 nM and 0.8 μl of template cDNA using the following general protocol: initial denaturation at 98°C for 10 seconds, a 3-step cycle including an denaturation at 98°C for 1 second, annealing for 5 seconds at a primer set specific temperature (58–61°C) and extension at 72°C during a predicted product size appropriate time (5–40 seconds) for 40 cycles and a final extension at 72°C for 1 minute. PCR products were separated by electrophoresis in 1% agarose gel. Amplification products were excised from gel and cleaned with the GRS PCR & Gel Band Purification Kit (GRiSP, Portugal) and sequenced at STABVIDA (Portugal). The resulting full ORF nucleotide sequences were deposited in GenBank: *Cpt1a* (KF570112) and *Cpt1c* (KF570111).

## Results

### Phylogenetic analysis of the Cpt1 gene family reveals three ancestral clades

**Fig 1:**
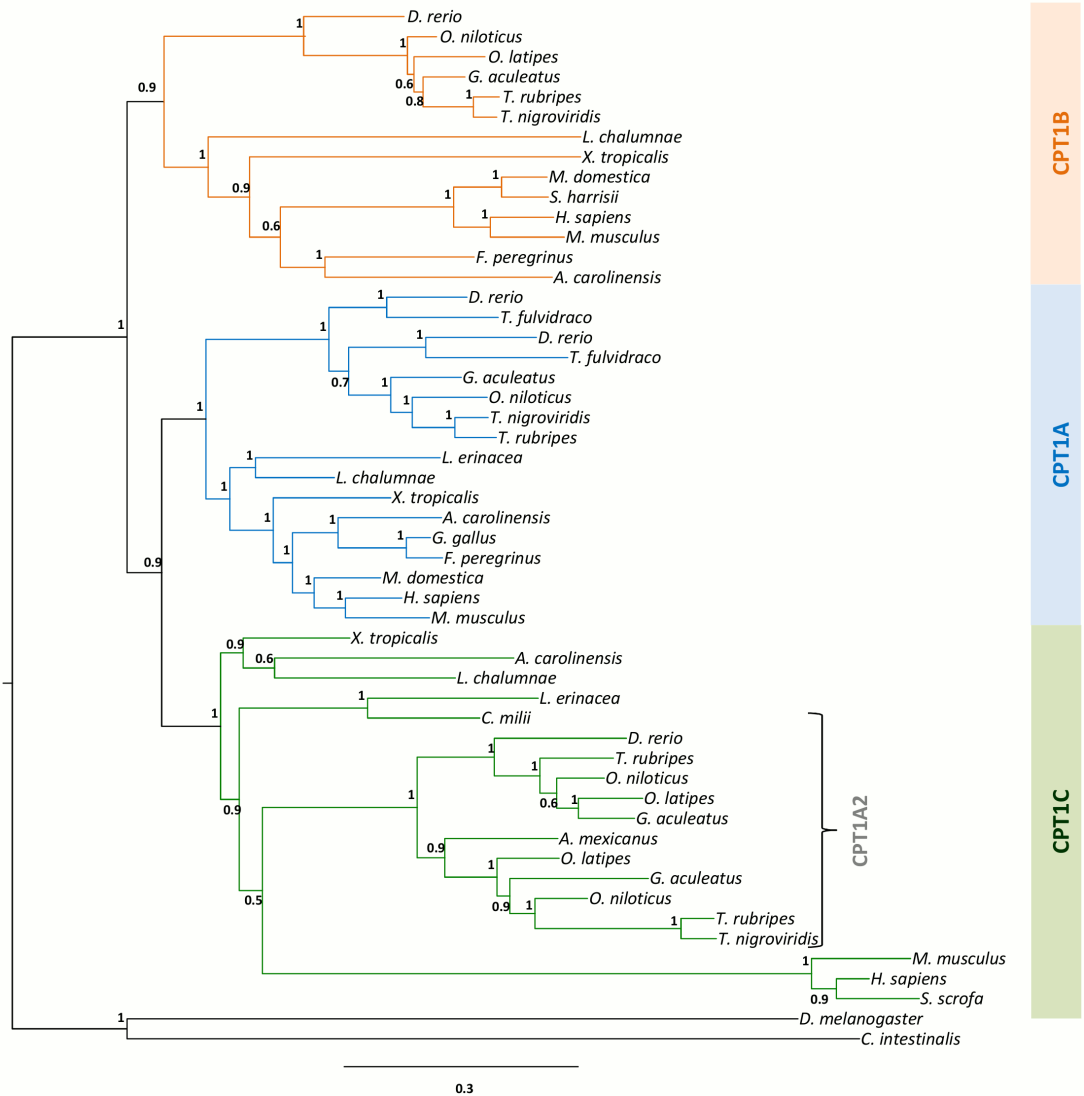
Molecular phylogenetic analysis of the *Cpt1* genes by Maximum Likelihood. Node values represent branch support using the aBayes algorithm. Accession numbers for all sequences are provided in the S1 Table.

We began by retrieving annotated and non-annotated CPT1-like protein sequences from genome databases of species representing all major vertebrate lineages (S1 Table). We next performed molecular phylogenetic analysis to address the overall evolutionary relationships of *Cpt1* genes. Phylogenetic analyses performed with both sequence alignments one containing gaps (S1 Fig) and the second without gaps (S2 Fig) rendered trees with similar overall topology (Fig 1 and S3A Fig). The inferred ML trees place invertebrate *Cpt1* genes outside a monophyletic group containing all vertebrate sequences (Fig 1). The later were divided into three well-supported groups encompassing *Cpt1a*, *Cpt1b*, and *Cpt1a2*/*Cpt1c* sequences respectively (Fig 1). The *Cpt1a* and *Cpt1b* clades were found to include sequences from teleosts, amphibians, coelacanth, birds and reptiles, and mammals. Contrary to previous findings, *Cpt1a2* is not unique to teleosts. Orthologues were identified in the *X*. *tropicalis*, *A*. *carolinensis*, and *L*. *chalumnae* (Fig 1). Surprisingly, the mammalian *Cpt1c* sequences robustly group with the *Cpt1a2* clade (see below). Additionally, mammalian *Cpt1c* orthologues are also apparently the least conserved, as indicated by their longer branch-lengths in the tree (Fig 1). Despite our searches, we were unable to locate an orthologue of *Cpt1c* in the available avian genomes, also confirmed by others in recent release of various avian genomes [22]. The *C*. *milii Cpt1* gene that is currently annotated as *Cpt1a* [10], robustly clusters with the *Cpt1c* group in our analysis (Fig 1).

### Synteny conservation of *Cpt1a* and *Cpt1b* loci

**Fig 2:**
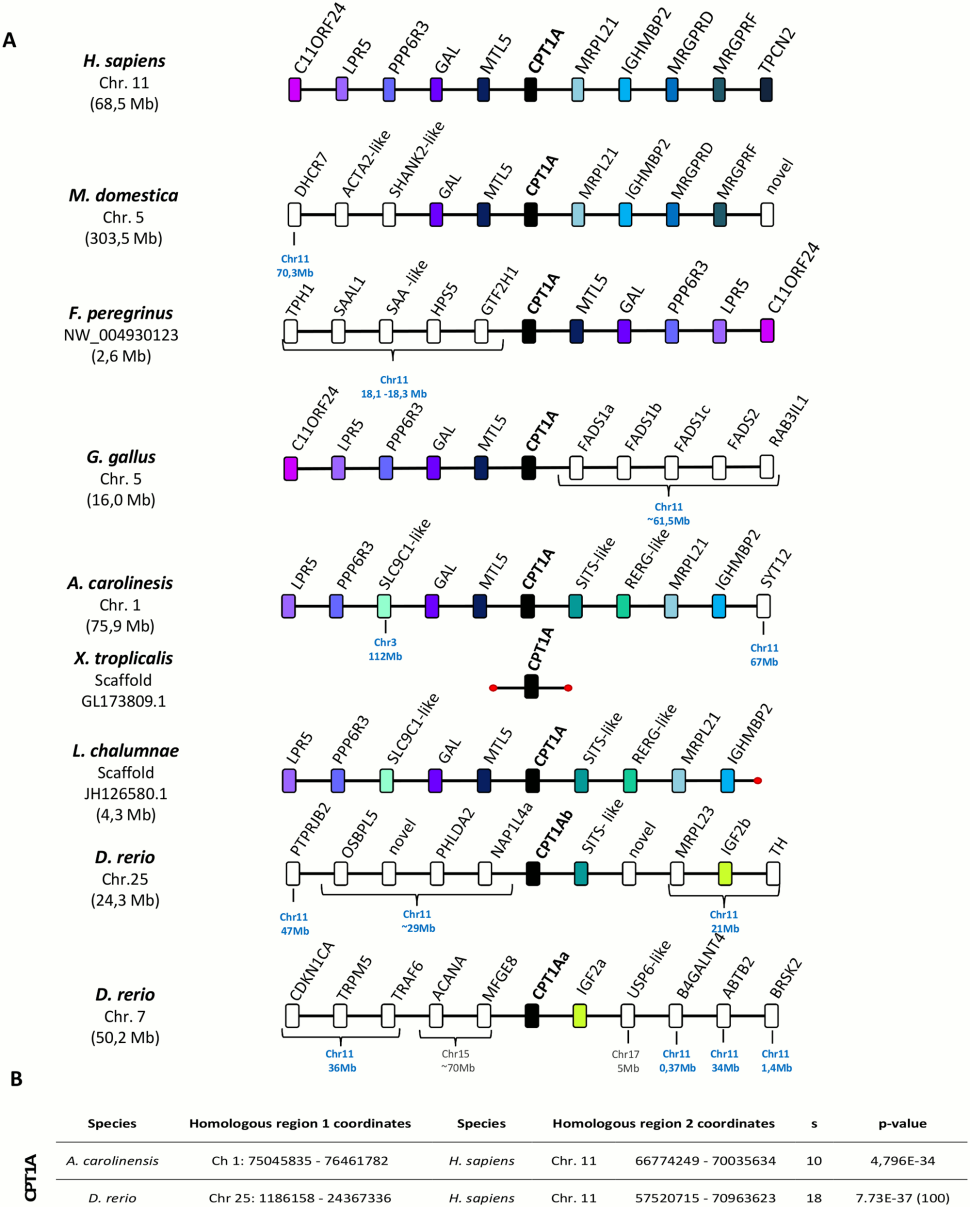
**A. Synteny maps of *Cpt1a* gene *loci* in selected vertebrate genomes**. Chromosome (Chr.) and location in mega base pairs (Mb) is given for the gene of interest in each species. The location of the *H*. *sapiens* orthologue is also given for non-conserved neighbouring genes in the other species analysed. Colour code denotes orthology relationships. Red dots indicate end of the chromosome or scaffold. **B. Statistical synteny analysis**. Reported p-values indicate the probability of identifying non homologous chromosomal segments, and S indicates the size of the chromosomal segment identified.

**Fig 3:**
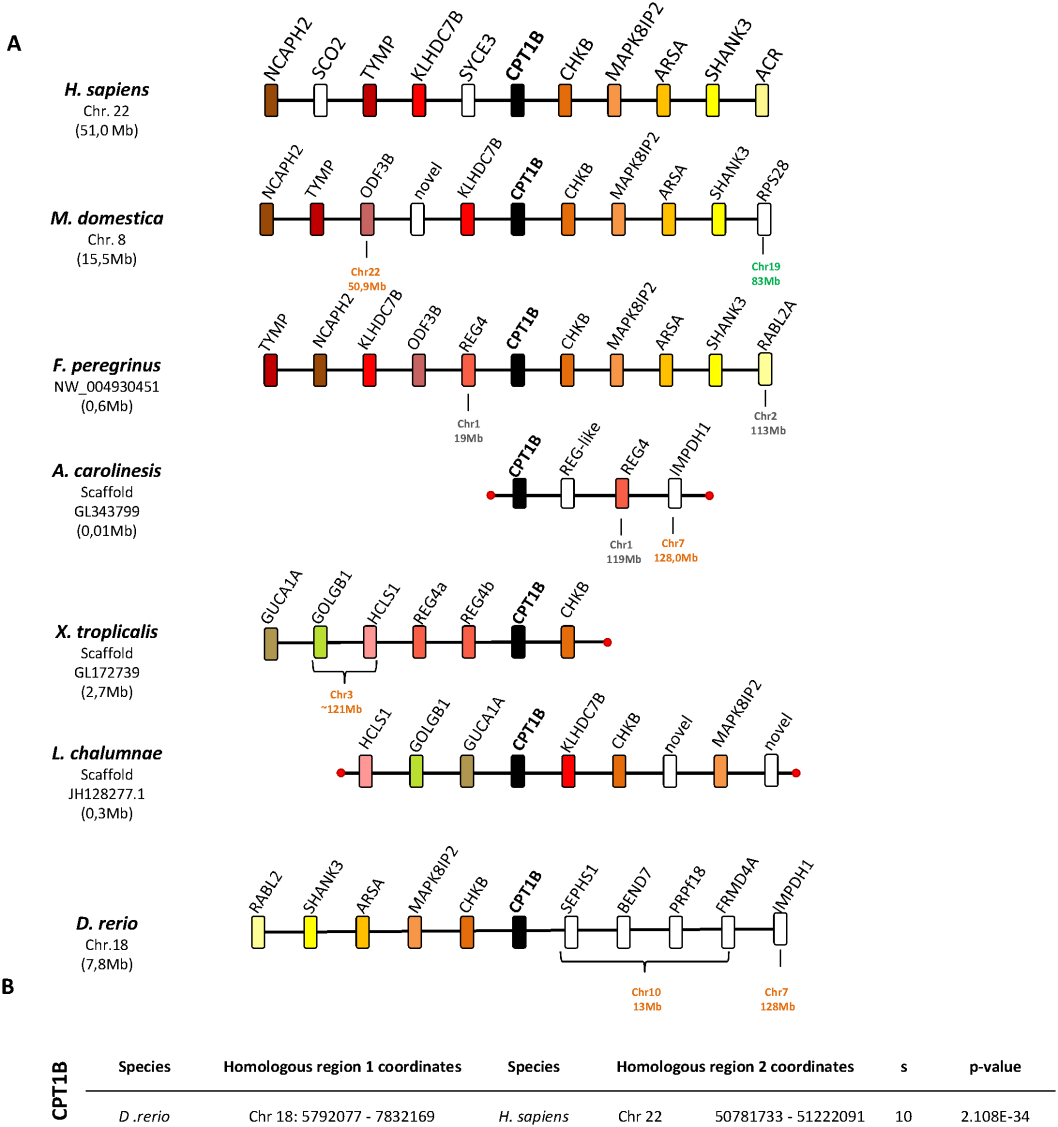
**A. Synteny maps of *Cpt1b* in selected vertebrate genomes**. Chromosome (Chr.) and position in mega base pairs (Mb) locations are given for the gene of interest in each species. The location of the *H*. *sapiens* orthologue is also given for non-conserved neighbouring genes in the other species analysed. Red dots indicate end of the chromosome or scaffold. **B. Statistical synteny analysis**. Reported p-values indicate the probability of identifying non homologous chromosomal segments, and S indicates the size of the chromosomal segment identified.

To further clarify the orthology/paralogy relationships of the *Cpt1* gene repertoire of the different lineages, we next examined the gene families adjacent to each *Cpt1* gene *locus* in a variety of species (Figs 2 and 3). The *Cpt1a locus* displays a high degree of synteny conservation. For example, *Mtl5* flanks *Cpt1a* in all of the examined Sarcopterygii species (Fig 2A), with the exception of *X*. *tropicalis* whose genome assembly at this *locus* is still very poor. In the paralogous *Cpt1aa* and *Cpt1ab loci* of *D*. *rerio*, the gene conservation is less evident, with the vast majority of genes having their *H*. *sapiens* orthologues mapping to chromosome 11 but at a distinct genomic region. However, adjacent to the fish *Cpt1ab* we found a novel gene family which although absent from mammals flanks the *L*. *chalumnae* and *A*. *carolinensis Cpt1a* orthologue (SIST-binding protein like) (Fig 2A). Comparative synteny statistical analysis was performed for *H*. *sapiens* vs *A*. *carolinesis* and *H*. *sapiens* vs *D*. *rerio* (Fig 2B). In both cases we find that the analysed chromosomal segments are orthologous to the corresponding *locus* in *H*. *sapiens*. In *D*. *rerio* the analysed chromosomal segment was expanded (gaps< = 100) to accommodate the highly rearranged nature of this *locus* in *D*. *rerio*. However the minimal number of genes was also proportionally increased, to maintain the statistical sensitivity. The analysis was not performed for *X*. *tropicalis* given that *Cpt1a* gene in this species is placed in an independent unplaced scaffold with no information on the neighbouring genes. Additionally, phylogenetic analysis of neighbouring genes *Mtl5* and *Sits-like*, supports the orthology of these sequences across different species (S4 Fig) and thus the common origin of this *locus*.

The gene composition of the *Cpt1b locus* also displays some degree of conservation in both Sarcopterygii and Actinopterygii species, except in *A*. *carolinensis* and *X*. *tropicalis* (Fig 3A). Even though the gene order is not exact, *Chkb*, *Arsa* and *Shank3* are all found in the proximity of *Cpt1b* in many of the examined species, providing strong support for their common origin (Fig 3A). Statistical analysis between *H*. *sapiens* vs *D*. *rerio Cpt1b locus* again indicates that these chromosomal segments are syntenic (Fig 3B). Here, statistical analysis was not performed for *A*. *carolinesis* and *X*. *tropicalis* given that *Cpt1b* gene is placed in a small scaffold in *A*. *carolinesis* or at the edge of the scaffold in *X*. *tropicalis*; in both cases lacking the minimal information regarding neighbouring genes, not allowing a confident statistical analysis of synteny. Additionally phylogenetic analysis of the neighbouring genes *Chkb* and *Arsa*, support that this genomic *locus* shares a common origin in the analysed species (S5 Fig).

### Locus composition supports the idea that *Cpt1a2* and *Cpt1c* genes are highly divergent orthologues

**Fig 4:**
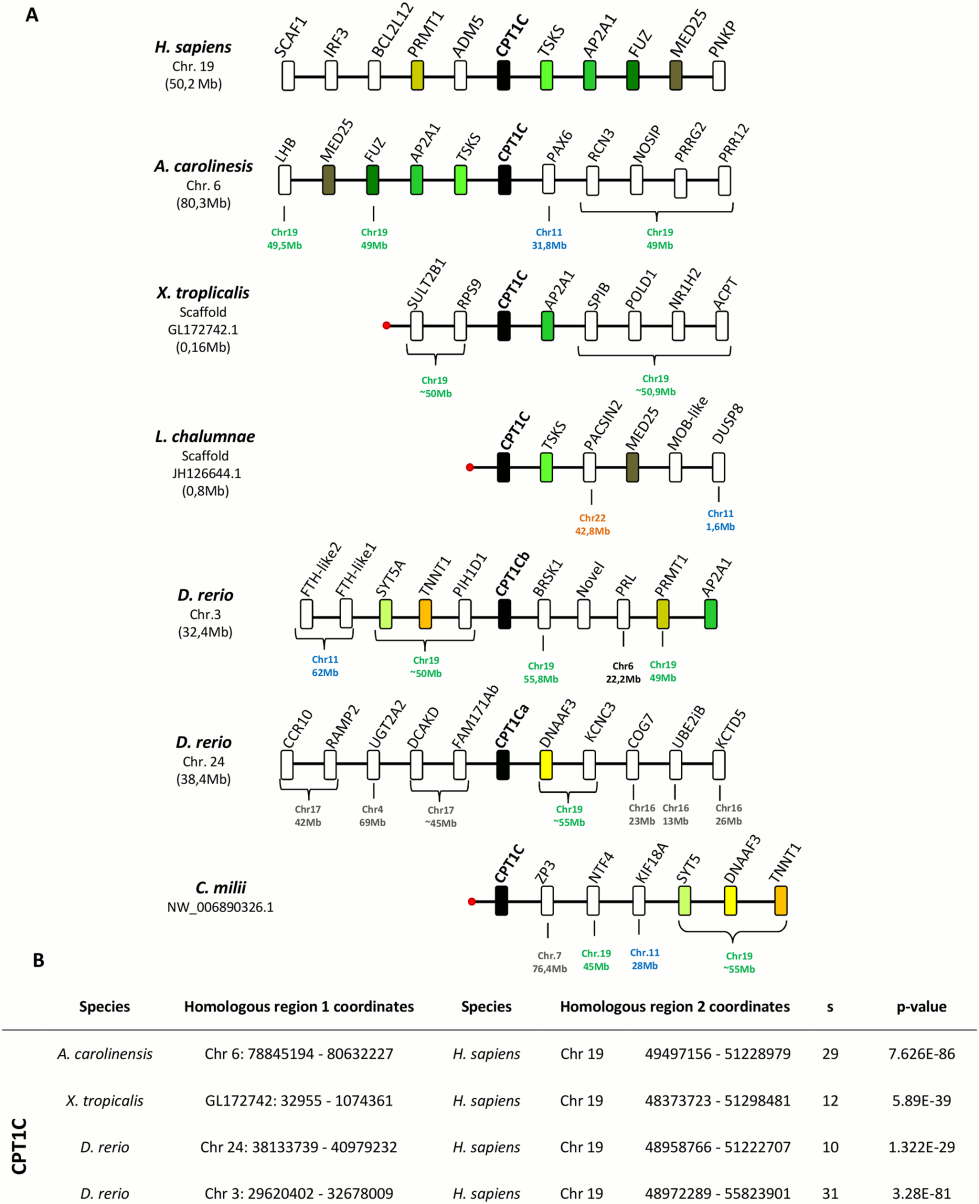
Synteny maps of *Cpt1c* in selected vertebrate genomes. Chromosome (Chr.) and position in mega base pairs (Mb) locations are given for the gene of interest in each species. The location of the *H*. *sapiens* orthologue is also given for non-conserved neighbouring genes in the other species analysed. Red dots indicate end of the chromosome or scaffold. Mapping data from the *C*. *milii* derived from [10]. B- **Statistical synteny analysis**. Reported p-values indicate the probability of identifying non homologous chromosomal segments, and S indicates the size of the chromosomal segment identified.

Previous reports described a new *Cpt1* gene, *Cpt1a2*, present uniquely in teleost species [5, 9] and suggested *Cpt1a2* to result from a duplication event in the teleost ancestor [9]. However, we have found orthologues, on the basis of phylogenetics, in all examined gnathostome species, except birds (Fig 1). To shed light into its evolutionary origin, we proceeded to investigate the *Cpt1a2* gene *loci* composition (Fig 4A). The *A*. *carolinensis* gene is flanked by *Tsks* similarly to *L*. *chalumnae*, while *Ap2a1* is also found close to *Cpt1a2* in all examined species, with the exception of *D*. *rerio Cpt1ca* and *C*. *milii Cpt1c* (Fig 4A). Nonetheless, we find that neighbouring genes such as *Dnaaf3*, *Kcnc3* (in *D*. *rerio Cpt1ca) and Ntf4* (in *C*. *milii*) have their human orthologues localizing to the *CPT1C locus*, establishing a conserved synteny within the analysed species (Fig 4A). In effect, detailed analysis shows that *Cpt1c* and *Cpt1a2* share a similar *locus* (Fig 4A), irrespective of the species where they occur. Additionally statistical analysis of *Cpt1c locus* synteny calculated for *H*. *sapiens* vs *A*. *carolinesis*, *X*. *tropicalis* and *D*. *rerio*, resulted in highly significant p-values in all cases (Fig 4B), indicating that these chromosomal segments are orthologous in the analysed species (Fig 4B). Both the phylogenetic and synteny analyses indicate that *Cpt1c* and *Cpt1a2* are most likely highly divergent orthologues. Thus, we propose that *Cpt1a2* from non-mammalian species should be renamed to *Cpt1c*. The occurrence of two genes in teleosts most likely results from the 3R teleost specific genome duplication [23]. In effect, *Fam171A2b* which flanks the *D*. *rerio Cpt1ca* has a teleost specific paralogue localizing to chromosome 3, the *locus* of origin of *Cpt1cb* (not shown).

### Cartilaginous fish have *Cpt1a* and *Cpt1c* orthologues

A *Cpt1a* gene has been recently described in a basal gnathostome, the *C*. *milii* [10]. However, both our phylogenetic and synteny analyses suggest that this is a *Cpt1c* gene (Figs 1 and 4). To clarify the complement of *Cpt1* genes in basal vertebrate lineages, we examined the repertoire of *Cpt1* genes in the *L*. *erinacea* and *C*. *milii*. Blast searches to the genome sequence and the transcriptome of both species identified two complete/incomplete *Cpt1*-like genes in both species. Phylogenetic analysis indicates that *L*. *erinacea* has *Cpt1a* and *Cpt1c* orthologues (Fig 1). These findings are also confirmed by the analysis of the *Cpt1c locus* composition in *C*. *milii* (Fig 4A) [10]. Careful inspection shows that the gene occurs at a similar genomic location to the *H*. *sapiens Cpt1c* (Fig 4A). Additionally phylogenetic analysis of neighbouring genes *Tnnt1* and *Dnaaf3* (S6 Fig) supports previous statistical analysis indicating that this chromosomal segment is orthologous between *H*. *sapiens* and *D*. *rerio* and allows us to extend this conclusion to the *C*. *milii Cpt1c locus*. Despite intensive searches to the genome sequence of the *C*. *milii*, as well as, with degenerate primer PCR in *L*. *erinacea*, we failed to isolate *Cpt1b* orthologues (not shown).

## Discussion

**Fig 5:**
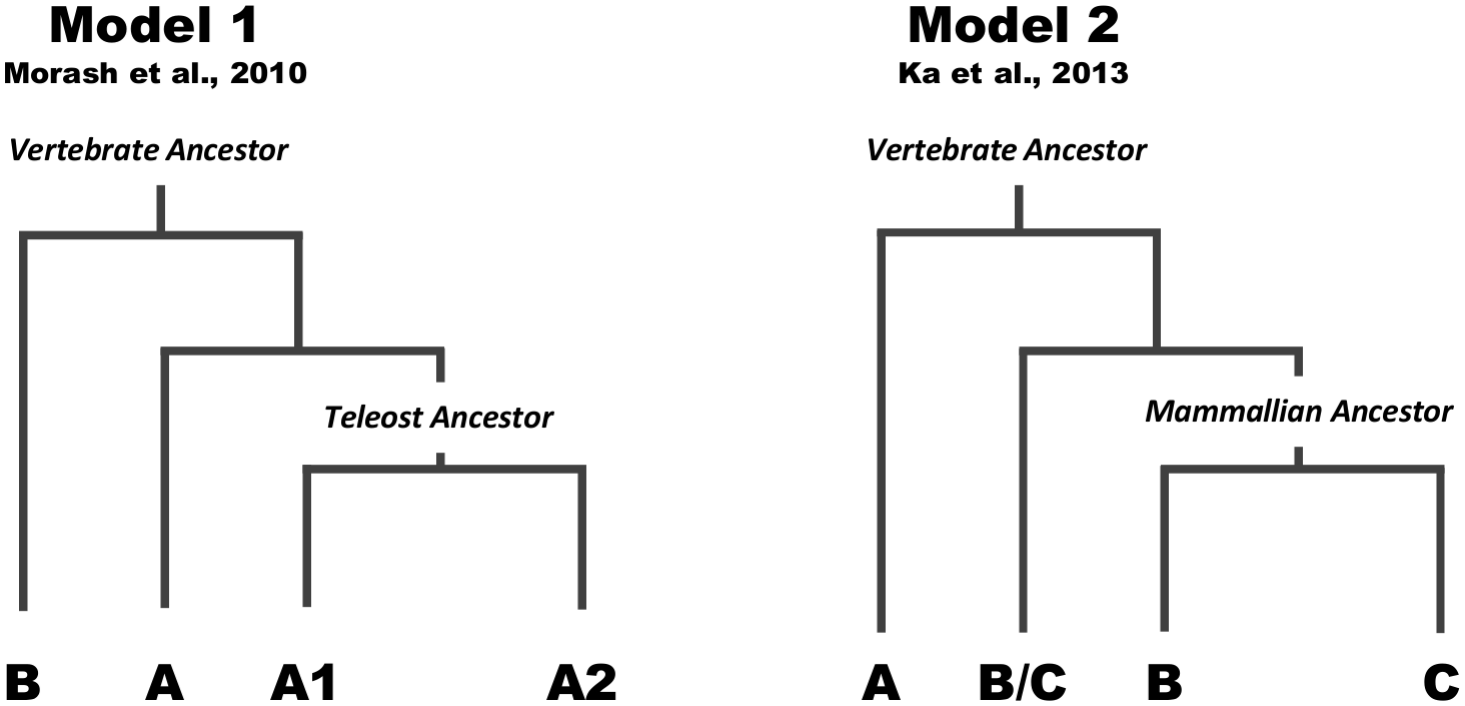
Schematic representation of two evolutionary scenarios of *Cpt1* genes. Model 1 derived from Morash et al. 2010 [9], and Model 2 derived from Ka et al. 2013 [8].

The conversion of long chain fatty acids into acylcarnitines, a fundamental step in the transport of long chain fatty acids to the mitochondria for β-oxidation, is catalyzed by CPT1. Thus, this enzyme plays an essential role in energy homeostasis, since it regulates fatty acid import for subsequent oxidation. Here, we set out to reassess the evolutionary history of *Cpt1* genes in vertebrate history, paying special attention to a basal gnathostome lineage, the chondrichthyans. These are known to have an unusual energetic metabolism without fatty acid oxidation in both skeletal and cardiac muscle [24]. Additionally, *Cpt1c* a so-called mammalian specific gene has an unclear origin and function. Several evolutionary models have been put forward to account for the reported *Cpt1* gene diversity in vertebrate lineages (Fig 5). Morash and co-workers (2010) proposed that a duplication in the ancestor of both fish and mammals gave rise to the *Cpt1a* and *Cpt1b* isoforms [9], (Fig 5 model 1), with a subsequent duplication generating *Cpt1a1* and *Cpt1a2* isoforms after the divergence of teleost fish; in an alternative scenario the *1a2* isoform was secondarily lost in mammals while retained in teleosts [9]. Extra specific genome duplications that took place in teleosts (e.g. 3R and 4R) would be responsible for the higher number of *Cpt1* genes in fish species (e.g. *Cpt1a1a* and *Cpt1a1b*) [9]. Nevertheless, this proposal did not address the origin and evolution of the puzzling *Cpt1c* gene, nor did it provide clear insight into the duplication history of *Cpt1a1* and *Cpt1a2* genes. So far, *Cpt1c* has been largely recognized as a mammalian novelty with no orthologues identified in non-mammalian genomes [7, 25]. On the basis of phylogenetics and chromosomal mapping of the *G*. *gallus Cpt1b* gene, it was proposed that *Cpt1c* and *Cpt1b* emerged in mammalian ancestry from the duplication of a *Cpt1b/c* gene after the divergence of sauropsids (Fig 5 model 2) [8]. Thus, the sauropsid *Cpt1b* would be pro-orthologous of mammalian *Cpt1b* and *Cpt1c* [8].

We have put these evolutionary scenarios to the test by comprehensively mining the genomes of extant species, representative of major vertebrate lineages, for phylogenetic and synteny analyses, in particular the chondrichthyans for which no information was available. We began by undertaking molecular phylogenetics and the recovered tree topology identified three well-supported groups (*Cpt1a*, *Cpt1b*, and *Cpt1c*/*Cpt1a2*) in contrast to previous reports [5, 8, 9]. We also found that *Cpt1a* was present in all of the examined lineages, with its origin dating to the vertebrate ancestor. Interestingly, the gene previously designated as *Cpt1a* in the *C*. *milii* fails to group here, and instead is part of the *Cpt1c*/*1a2* group. In addition, we were able to identify a *Cpt1a* orthologue in a cartilaginous species.

We found that, in phylogenetic trees, mammalian *Cpt1c* genes branch together with the previously designated *Cpt1a2* genes, but with longer branch lengths. Thus, *Cpt1c* could be a highly divergent *Cpt1* gene without any counterpart in non-mammalian species, or a divergent orthologue of a described *Cpt1* gene. To test these possibilities we examined the synteny of *Cpt1* gene *loci*. *Cpt1a* and *Cpt1b loci* are conserved across the tested species, a clear indication that they emerged early in vertebrate evolution. Strikingly, we also found that mammalian *Cpt1c* and non-mammalian *Cpt1a2* have a similar *loci* composition, again suggesting that they are highly divergent orthologues. Our analysis allowed the simultaneous clarification of the origin of both mammalian *Cpt1c* and non-mammalian *Cpt1a2*.

**Fig 6:**
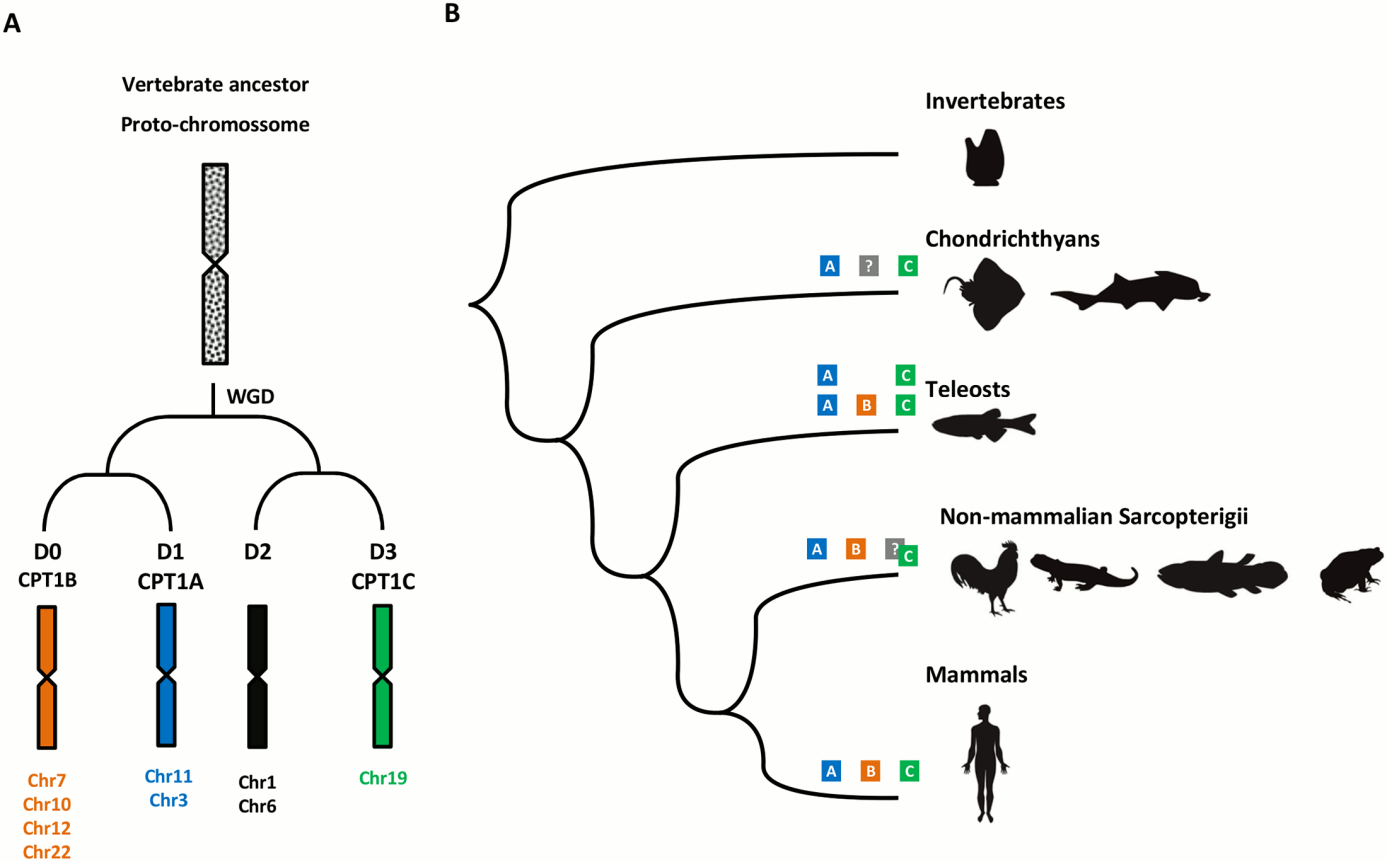
Linkage group of Cpt1 genes upon genome duplications of the ancestral proto-chromosome D (details from Nakatani et al. (19) (A), and the proposed evolutionary model of the Cpt1 gene family in vertebrates (B). WGD–whole genome duplication.

*Cpt1a* and *Cpt1b* are located in genomic regions related by genome duplications in vertebrate ancestry, the so-called 2R WGD (Fig 6A) [26, 27]. Interestingly, the genomic region harbouring *Cpt1c* is part of the same linkage group [10, 26]. In this context, we put forward a model that includes the duplication of a single copy *Cpt1* gene in the ancestor of vertebrates as a result of 2R, with the retention of 3 genes and the loss of a fourth paralogue (Fig 6B). This gene complement expanded in teleosts with the lineage independent genome duplications, 3R and 4R. Based on the present data we suggest that after the divergence of sauropsids, *Cpt1c* underwent an accelerated rate of evolution and functional divergence in mammals (Fig 6B). Consequently, despite the common origin, mammalian *Cpt1c* has most likely acquired a novel function after the divergence of sauropsids. In effect, the information currently available indicates that the mammalian CPT1C function and biology is rather unique. In contrast to other CPT1 enzymes it does not localize to mitochondria but to the ER [28]. Regardless of its function, it is clear that mammalian CPT1C does not mediate mitochondrial transport of long chain fatty acids. In fact, given its oxygen demand, generation of toxic oxidative by-products and slow rate of ATP production, the brain does not rely on mitochondrial fatty acid β-oxidation, favouring glucose and liver-derived ketone bodies as source of energy [29]. Given the striking divergence of mammalian CPT1C, in both its N-terminal domain, suggested to determine protein localization and regulate activity, and C-terminal catalytic domain [30, 31], further studies are needed to elucidate their molecular function.

Our results also strongly suggest that *Cpt1* gene retention after 2R varied in different lineages, similar to what has been described for other gene families [26, 32, 33]. For example, we were unable to find an orthologue of *Cpt1c* in birds and *Cpt1b* in chondrichthyans. Strikingly, the absence of *Cpt1b*, the “*muscle isoform*”, directly correlates with the use of ketone bodies and not fatty acids as oxidative fuels in muscle of elasmobranches [24]. If confirmed, the uncommon muscle energy metabolism elasmobranch fishes would be linked to a single event of gene loss.

## Conclusion

Our approach has provided additional clarification on the evolution of *Cpt1* genes and shows that the mammalian *Cpt1c* is probably a rapidly evolving orthologue of *Cpt1a2* in non-mammalian vertebrates. We propose that *Cpt1a*, *Cpt1b* and *Cpt1c* emerged in vertebrate ancestry as the result of genome duplications. *Cpt1c* is not a mammalian innovation (though its function probably is) since synteny and phylogenetics shows that divergent orthologues can be found in other classes. We suggest that differential loss, extra lineage-specific duplications, and an accelerated rate of sequence divergence have all modelled the history of the *Cpt1* gene family in vertebrates, with consequences in energy metabolism.

## Supporting Information

S1 Fig MAFFT Sequence alignment with gaps. (PDF)

S2 Fig MAFFT Sequence alignment without gaps. (PDF)

S3 Fig Alternative phylogenetic analysis supporting main phylogenetic analysis. —Maximum likelihood phylogeny (gap alignment) using aBayes for branch support (Figure A), Maximum likelihood phylogeny with 1000 bootstraps replicates (Figure B), Bayesian Phylogenetic analysis (Figure C) and Phylogenetic analysis using Neighbor-Joining method (Figure D).
(PDF)

S4 Fig Supporting Phylogenetic analysis supporting *Cpt1a* comparative synteny maps. (PDF)

S5 Fig Supporting Phylogenetic analysis supporting *Cpt1b* comparative synteny maps. (PDF)

S6 Fig Supporting Phylogenetic analysis supporting *Cpt1c* comparative synteny maps. (PDF)

S1 Table List of sequences used for the molecular phylogenetic analysis and respective accession numbers (GenBank or Ensembl). (PDF)
