# Supplementary material for: The Origin and Diversity of Cpt1 Genes in Vertebrate Species
Source: PLoS One. 2015 Sep 30;10(9):e0138447. doi: 10.1371/journal.pone.0138447 (PMC4589379; doi:10.1371/journal.pone.0138447)
Supplement: S1 Fig — (PDF) [file pone.0138447.s001.pdf]

Supporting information 1 Fig: MAFFT Sequence alignment with gaps

```
Hsa1C      MAEAHQAVGF RPSLTSDGAE VELSAPVLQE IYLSGLRSWK RHLSRFWNDF LTGVFPASPL SWLFLFSAIQ L-AWFLQLDP SLGLMEKIKE LLPDW-----  94
SSc1C      .......... ..P.....G. ....P.L... .......... .......... .I........ .......... .-.C...... .......... .....-----  94
Mmu1C      .......SSL LS..S..... ....S..W.. ...CA..... ...W.V.... .A..V..T.. .......T.. .-.CL..... .......... .....-----  94
Hsa1A      ........A. QFTV.P..ID LR..HEA.RQ ......H... KKFI..K.GI I...Y....S ...IVVVGVM T-TMYAKI.. ...IIA..NR T.ETA-----  94
Mmu1A      ........A. QFTV.P..ID LR..HEA.KQ .C....H... KKFI..K.GI I........S ...IVVVGVI S-SMHTKV.. ...MIA..NR T.DTT-----  94
Mdo1A      ........A. QFTV.P..ID LR..HEA.KQ ......H... KKFI..K.GI I...Y....S ...IVVVGVM S-TMYAKV.. ...IIA..NQ T.DMT-----  94
Gga1A      ........A. QFTV.P..ID LRM.HEA.KQ .....VH... KKFI..K.GI I...Y....S ...IVVVGVM S-TMYAKI.. ...IIA..NR T.DTT-----  94
Fpe1A      ........A. QFTV.P..ID LRM.HEA.KQ .....VH... KKFI..K.GI I...Y....S ...IVVVGVM S-TMYAKI.. ...IIA..NR T.DTT-----  94
Aca1A      ........A. QFTV.P..ID LRM.HEA.RQ ......Y... KKFI..K.GI I...Y....S ...IVVVGVM S-TMYAKI.. ....IA..NR T.DIT-----  94
Xtr1A      ........A. QFTV.P..ID LR..HEA.RQ ......H... KKFI..K.GI ......S..S ...IVVVGVT SASMYTKV.. .F.II...NN A.SAT-----  95
Lch1A      ........A. QFTV.P..ID LR..HEA.RQ ......H... KKFI..K.GI I...Y....S ....VVVGVV S-TMYTRV.. ...MIAR.SQ R..VT-----  94
Ler1A      ........A. QFTV.P..ID LQ..HEA.KQ V......... KRFR.YK.GI ....Y....S ....VVV.VI A-TMYARV.. ...MI...RC H..TN-----  94
Xtr1C      ........A. QFTV.PE.ID LR..HEA.KQ V......... KKFA.VK.S. I...Y....S ....VVI..M G-TLYARV.. .M.MI..... H..AS-----  94
Ler1C      ........A. QFTV.PE.ID LQ..RHA.K. .....I.... KKCN.IK.N. M...Y....S ..I.VVLTVV G-TMYTRV.. .M..IAMLR. HI.LR-----  94
Cmi1C      ........A. QFTV.PE.ID LQ..HQA.K. .....V.A.. KRCA.IR.NI V...Y....S ....VALTVV G-TMYTRV.. .M.MIG.... H..VK-----  94
Aca1C      ........A. QFTVSPE.ID LQ..HVAFK. .....V.... KKCNQLR.G. V...Y....S ....MVT..L V-TQYSR... .M.MID.... H..VS-----  94
Lch1C      ........A. QFTV.PE.ID LK..HEA.KQ VF...Q.... KR.A.LK.S. I..MY....S .....VI..M A-TLYARV.. .M.MID.... H..VKVQEFY  99
Tni1A      ........A. QFTV.P..ID LQ.CHEA.RQ V.....H... KRFI..K.GV M...Y.G..A GFMIVVGSYM SYNKYNR... .M..VV.LGQ YI.IG-----  95
Tru1A      ........A. QFTVSP..ID LQ.CHEA.RQ ......H... KRFI..K.GV M...Y.G..G GFMIVVGSYM SYNKYN.... .M.FVV.LGQ YI.IS-----  95
Oni1A      ........A. QFTV.PE.ID LH..HEA.RQ V....IH... KRFV..K.GI M...Y.G..A GFSIVVGSYM AYNK.K.... ....FT.LGQ HI.IS-----  95
Gac1A      ........A. QFTVSP..ID LQ..HEA.RQ ......H... KRFI..K.GV M...Y.G..G GFMVVVVSYM SYNKYQ.... ....IA.LGQ HM.IS-----  95
Dre1Aa     ........A. QFTV.P..ID LH.CHEA.RQ .....IH... KRFI..K.GV M...Y.G..S GLVVVLVGYM SSTKYAKI.. ...ILT.LST H..VS-----  95
Tfu1A2     ........A. QFTV.P..ID LQ.CHEA.RQ ....A.H... KRFI..K.GV M...Y.G..T GL.GVVGGYL IFTKYANI.. T..VVA.LAP H..VC-----  95
Dre1Ab     ........A. QFTVGP..ID LQ..HEA.RQ V....IH... KKFI..K.GI .N..Y.G.AP GFVLVLAGYL GR.QY.KV.. ....LF.LGN YV.IS-----  95
Tfu1A1     ........A. QFTVSP..ID LQ..HEA.RQ V.....H... KKFI..K.GI M...Y.G.AP GLMLVLAGYM GR.KYA.V.. ....VFR.GK YV.IS-----  95
Ola1Ca     ........A. QFTV.PE.ID LR..HQA.S. .....V.... KRII.IK.SV I...Y....S ....VVI..L A-TMYTRS.. .M.IIA..Q. H..VS-----  94
Oni1Cb     ........A. QFTV.P..ID LQ..HQA.T. .....M.... KRIV.VK.SV I...Y....S ....VVI..L A-TMYTRS.. .M..IA..Q. H..VS-----  94
Ame1C      ........A. QFTI.PE.ID LQ..HEA.RQ V......... KRIV.LK.NV I...Y....S ....VVI..L A-TMYTRS.. .M..IA..Q. H..AS-----  94
Gac1C      ........A. QFTVSP..ID LH..HQA.T. .....V.... KRVIGLK.SV V...Y...LS ....VVI..L A-TMYTRS.. .M..IA..Q. H..RP-----  94
Tru1C      .......... EFTVIPE..D AQ..HQA.T. ....AVN... KCII.IK.SV IR..Y....F ....VVI..L A-TMYTRS.. .M..IA..Q. N..VS-----  94
Tni1C      .......... EFTVIPE.TD AQ..HQA.T. ....AVH... KCII.IK.SV IK..Y....F ....VVI..L A-TMYTRS.. .M..IA..Q. N..VS-----  94
Ola1Cb     ........A. QFTI.PE.ID LQ..YQA.NQ .....V.... KRV..MR.RV IK..Y....S ....VAIG.L A-TIYM.S.. .M..IAE.QQ R..LS-----  94
Oni1Ca     ........A. QFTI.PE.ID LQ..YQA.NQ .....V.... KRV..VR.SL IK..Y....S ....VAIG.L A-TMYM.S.. .M..IT..QQ H..LS-----  94
Gac1C      ........A. QFTI.PE.ID LQ..HQA.NQ .F...V.... KRV..MR.RV IK..Y....S ....VVI..L A-TMYMRS.. .M..IKM.QQ H..LR-----  94
Tri1C      ........A. QFTI.PE.ID LQ..YQA.NQ .....V.... KRV..VR.SL IK..Y....S ....VSI..L A-TMYMRS.. .M..IT..QH H..LS-----  94
Dre1C      ........A. QFTISPE.IN LH..YQA.NQ .......... KRI..IK.RI IK.AY....S ....IVI..L A-TMYM.S.. .M..IA..Q. H..LS-----  94
Dre1B      ........A. QFTV.P..ID LQ..RE..KH ..I..VT... KRAI..K.SV ....Y....S ....VVI..M S-TMYARI.. .M.TID...T S..VS-----  94
Oni1B      .......... QFTVRP..VD FK..QE.IKN .....VTA.. KKAIQ.K.GV .A..Y....S ...IVVI.MM S-SLYIHI.. ...M.DA... N..YR-----  94
Gac1B      .......... QFTVRP..VD LK..QE.IKT .....VTA.. KRAIQ.K.GV .A..Y....S ...IVVI.MM S-SLYIRI.. ...MI.ALQ. N..HR-----  94
Ola1B      .......... QFTVRP..V. LK..QE.IKN ......TV.. KKAIQ.K.SV ....Y....S ...IVVI.MM S-SLYTNT.L ...MIDA... N..HR-----  94
Tru1B      .......... QFTVRP..V. LK..QE.IKN ......TA.. KRAIQ.K.GV .A..Y....S ...IVVI.MM S-SLYTRV.. ...MI.AM.. N..YR-----  94
Tni1B      .......... QFTVRP..V. LK..QE.IKN ......TA.R KRAIQ.K.GV .A..Y....S ...IVVI.MM S-SLYI.V.L ...MI.AM.. N..YR-----  94
Hsa1B      ........A. QFTV.P..VD FR..REA.KH V....IN... KR.I.IK.GI .R..Y.G..T ...VVIM.TV G-SS.CNV.I ....VSC.QR C..QGCG---  96
Mmu1B      ........A. QFTV.P..VD FR..REA.RH .....IN... KR.I.IK.GI .R..Y.G..T ...VVVM.TV G-SNYCKV.I .M..VDC.QR C..ERYG---  96
Mdo1B      ........A. QFTV.P..VD FQ..REA.KH V....IN... KR.I.IK.GI .R..Y.G..T ...VVVMTTM G-SSYCNV.L .M.MICC.RK YI.EGCC---  96
Sha1B      ........A. QFTV.P..VD FQ..REA.KH V....IN... KRFI.IK.GI .R..Y.G..T ...VVVM.TM G-SSYCNV.I .M.MICH.RK YI.EGCS---  96
Aca1B      ........A. QFTV.PE.VA FQ..REA.KQ .....VS... KR.A.LK.SI ......GT.S G..AVVAVTV G-.TYFGI.V .V..IFR.QK R..NSCR---  96
Fpe1B      ........A. QFTV.PE.LD FH..REAVRQ L..A.IS... KR.V.AK.S. ....Y....S ..MVVVM.TA G-SFYC.V.. ...MIAR.RH C..ES-----  94
Xtr1B      ........A. QFTV.P..ID LQ..HEA.RH LW....VACR KR.ITLKSSV .S..Y....S T.FAVVAMTL G-SLYGK... ....IT..NS I..GK-----  94
LchB       ---------- ---------- ---------- -----.MN.L H--VFVQ.SL ....Y....S ...VVVV.TI G-TRYVKM.. ...IIDY.RS AI.RS-----  57
Dm         .....A..A. SFAI.HE.FD INYDHE..NL VWN..V.... KR.A.AR.GV RN..Y..HIQ .LWLISAIAL G-LH.AGYQA PFN.TNR.LV H..SN-----  94
ci         .....A..A. SF.V.QE.LN .QI.HEAM.A V.F..V...R KR.T..R.RV .S....VKYT .LVG.TAIVL .-.S..SY.I TW.FKNNRTN II.PR-----  94


Hsa1C      ---GG----- -QHHGLRGVL AAALFASCLW GALIFTLHVA LRLLLSYHGW LLEPH---GA MSSPTKTWL- -ALVRIF--- ------SG-R H-PMLFSYQR  169
SSc1C      ---.A----- -...R...I. .......... .......... .......... ...T.---.V .........- -.......-- -------.-. .-........  169
Mmu1C      ---..----- -...Q.Q.F. S..V...... .......... ......H... .....---.. .........- -.......-- -------.-. .-.R...F..  169
Hsa1A      ---NC----- -MSSQTKN.V SGV..GTG.. V...V.MRYS .KV....... MFTE.----.K ..RA..I.M- -GM.K..--- -------.-. K-...Y.F.T  169
Mmu1A      ---.R----- -MSSQTKNIV SGV..GTG.. V.I.M.MRYS .KV....... MFAE.----.K ..RS.RI.M- -.M.KV.--- -------.-. K-...Y.F.T  169
Mdo1A      ---.S----- -MSSQTKNIV SGI..GTG.. VT..VAMRYS .KM....... MFAEY---.K L.RG.RI.M- -GM.K..--- -------.-. K-...Y.F.T  169
Gga1A      ---.Y----- -MSNQTQNIV SGI..GTG.. V...V.MRYS .KM....... MFAE.----.K L.AG..L.M- -T..KL.--- -------.-. K-...Y.F.T  169
Fpe1A      ---.Y----- -MSNQTQNIV SGV..GTG.. V...V.MRYS .KM....... MFAE.----.K L.AG..F.M- -...KL.--- -------.-. K-...Y.F.T  169
Aca1A      ---.Y----- -MSNQTQNIV SGV..GTG.. V....SMRYT .KM....... MFAE.----.K L.TG..I.M- -T..KL.--- -------.-. K-...Y.F.T  169
Xtr1A      ---.Y----- -MTPQTQNIV SGV..GTG.. VS..A.MRYS .KK....... MF.E.----.K L.AS.RI.M- -GM.KLL--- -------.-. K-...Y.F.T  170
Lch1A      ---TH----- -LSSQSQNIV SGV..GTG.. V..F..MRYS .KM....... MFIE.----.K VPFG.RI.I- -R..KL.--- -------V-C K-...Y.F.T  169
Ler1A      ---.Y----- -LSNQSESI. SGL..STG.. I..V..MRQT .K........ MFV..----.K TPTSV.L.M- -LI.K..--- -------.-. K-.LTY.F.T  169
Xtr1C      ---SY----- -LS.QGQSIV S.L..STG.. F...M.MRFI .KQ....... MY.Q.----.K ..AT..L..- -...K..--- -------.-. N-...Y...A  169
Ler1C      ---RY----- -MS.HGQSMV S.M...TG.. L....VMRQI .KT....... MF.E.----.K V..T..V.F- -T..K..--- -------.-. K-...Y.F.N  169
Cmi1C      ---RC----- -LS.QGQNIM S.L...TV.. L...YMMRQI .KT....... MF.E.----.K A.NM.RI.F- -TM.K..--- -------.-. K-...Y.F.A  169
Aca1C      ---.Y----- -LSDQGMNI. S..T.STV.. L...M.MRSI .KM..C.... MY.E.----.K ..NT..I..- -...KM.--- ------A.-. K-...Y...A  169
Lch1C      QTARTKTNPT QITDKRINL. CTN.YQCGIY SEILL----- ---------- MF.Q.---NR V.LT..I.I- -T..K..--- -------.-. K-...Y...A  168
Tni1A      ---RY----- -LSTDTQKIV GGV.VGTS.. VTI.MIMRNV .KS...W... MH.R.---.S V.WTSRA.M- -L..KV.--- -------.-. K-...Y.F.N  170
Tru1A      ---RY----- -LSTDTQRIV GGV.VGTG.. VTI.MIMRNV .KS...W... MY.R.---.S I.WTSRA.M- -L..KV.--- -------.-. K-...Y.F.N  170
Oni1A      ---RY----- -MSTDSQKIV GGI.VGTS.. VTI.MIMRTV .KS...W... MQ.R.---.S L.WSSRI.M- -V..KV.--- -------.-. K-...Y.F.N  170
Gac1A      ---RY----- -MSTDSQKIV GGV.VGTG.. VSI.MIMRSV .KS...W... MYTS.---.S VAWS.RL.M- -V..KV.--- -------.-. K-...Y.F.N  170
Dre1Aa     ---KY----- -ITEDGQRIV GGV.VGTG.. I.VT.VMRN. .KY...W... MFNQ.---.T L.LK..I..- -V..KL.--- --------.-P K-...Y.F.S  170
Tfu1A2     ---KY----- -ITEDTQQIV GGI.VGTGV. T.V..FMRNM .KC...W... MYNR.---.T ..IR..I..- -...KL.--- --------.-P K-...Y.F.S  170
Dre1Ab     ---RY----- -MSEQKQMLV GGVMVGTG.. I.I..GMRTV .KG...W... MFAS.---.R .TWKIRL..- -VF.KV.--- --------.MQ T-...Y.F.N  171
Tfu1A1     ---KY----- -MSLDNQSRV GGI.VGTG.. VVI...MRSI .KG...W... MR.R.---.S LTWK.QI..- -V..KV.--- --------.-M KT.N.Y.F.T  171
Ola1Ca     ---QS----- -MSSQCQA.V S.V..STM.. LL....MRLC .KQ.....R. MF.Q.---.K ..TT..I.V- -.......-- -------.-. K-.L.Y...G  169
Oni1Cb     ---QS----- -MSTQCQA.V S.V..STM.. LM....MRLC .KQ.....R. MF.Q.---.K ..TT..I.V- -.......-- -------.-. K-.L.Y...G  169
Ame1C      ---.S----- -MSMQCQT.V S.V..STL.. LS....MKLC .KQ.....R. MF.Q.---.N V.TT..V.V- -M.......-- -------.-. Q-.L.Y...G  169
Gac1C      ---------- ------YVM. S.S.AVTM.. LL....MRMC .KQ.....R. MF.K.---.K ..NT..V.V- -VS.ASHRSL FVTVNQPS-. K-.L.Y...G  171
```

```
Tru1C         ---QS----- -LSTQCKVL. S.VI.STM.. LL....MRLC .KQ.....R. MF...---.K ..TT..V.V- -......--- ------..-. K-.R.Y...A  169
Tni1C         ---QS----- -LSTQCKVL. S.VI.STM.. LL....MRLC .KQ.....R. MF...---.K ..TT..V.V- -......--- ------..-. K-.R.Y...G  169
Ola1Cb        ---LHVS--- -LSAQGQTMV S.LV.STL.. LS..LA.RFC .K......Q. MF.Q.---.R V.NT..V.V- -T.L.LL--- ------..-. K-.L.Y...T  171
Oni1Ca        ---FF----- -LFTQGQTMV S.LV.STL.. LS..LA.RFC .K......Q. MF.K.---.R I.NT..V.V- -P...LL--- ------.S-. K-.L.Y...S  169
Gac1C         ---QPF---- -LSTQGQTM. S.LV.STL.. LS..LA.RFC .K......Q. MF.Q.---.R V.NT..V.V- -T.L.LL--- ------.S-. K-.L.Y...T  170
Tri1C         ---LHMS--- -LSAQGQTM. S.LV.STL.. LS..LA.RFC .K......R. MF.Q.---.R V.NF..V.V- -T.LGLL--- ------.S-. K-.L.Y...T  171
Dre1C         ---LS----- -LSPQGQTM. S.L..STL.. MS..L..RFC .K......R. MF.Q.---.H ..TK..V.A- -T..KLL--- ------.S-. K-.L.Y...T  169
Dre1B         ---EF----- -MTVQTQT.. S.I...TG.. LSV..L.RYL .KA.....A. IF.S.---.K ..YS..V..- -S..KLL--- ------..-. R-.L.Y.F.G  169
Oni1B         ---DC----- -MSVQT.A.. S.I...TG.. LF..YL.RYT .KA....... IF.S.---.K ..TS..V..- -C..KM.--- ------..-. R-.L.Y.F.A  169
Gac1B         ---DY----- -LSVQS.A.. S.I...TG.. LF..YL.RYM .KA....... IF.S.---.K ..RS..V..- -S..KM.--- ------..-. R-.L.Y.F.A  169
Ola1B         ---.Y----- -MSAQT.A.. S.I..GTG.. LF..YL.RYT .KA....... IF.S.---.K ...S..L..- -Y..KM.--- ------..-. R-.L.Y.F.A  169
Tru1B         ---DC----- -MSVQT.A.. S.I...TG.. LF..YL.RYT .KA....... IF.S.---.K ..TS..V..- -T..KM.--- ------..-. R-.L.Y.F.A  169
Tni1B         ---D.----- -MSVQT.A.V S.I...TG.. LF..YL.RYT .KA....... IF.S.---.K ..TS..V..L QT..KM.--- ------..-. R-.L.Y.F.A  171
Hsa1B         ---PY----- -.TPQT.AL. SM.I.STGV. VTG..FFRQT .K...C.... MF.M.---.K T.NL.RI.A- -MCI.LL--- ------.S-. .-...Y.F.T  171
Mmu1B         ---HF----- -GTPQTEAL. SMVI.STGV. ATG..FFRQT .K........ MF.M.---SK T.HA..I.A- -IC..LL--- ------.S-. R-...Y.F.T  171
Mdo1B         ---RY----- -LTLQT.TLI SVGI.STGV. VTG..LFRQT .K........ MF.L.---.Q T.RT..I.A- -IC..LL--- ------.N-. R-...Y.F.T  171
Sha1B         ---NY----- -LTLQT.TLI SVGI.STGV. VTG..LFRQT .K........ MF.M.---.Q T.RI..I.A- -IC..LL--- ------.N-. R-...YTF.T  171
Aca1B         ---RC----- -LGVRS.SL. S.LI.S.GA. MLGVLLRRQ. .......... MF...---.K TRPS..I.A- -SM..VM--- ------..-. .-...Y.F.T  171
Fpe1B         ---RL----- -LSYES.TMV STVI.STGA. LSAVLLFRQ. .K........ MF...---.K ..RS.RI.V- -..MKVL--- ------.I-. K-.L.Y.F.T  169
Xtr1B         ---SI----- -LAPVS.T.. S.VI.S.GV. VSG.LIYRQT ..I....... MF...---KK T.MK..I.A- -GCMK.M--- ------.S-. Q-...Y.F.M  169
LchB          ----C----- -MSFRS.TL. S.T...TGV. VTG.LAVRYS ..A..C.... MF...---.S TRIR.RI.A- -T..K..--- ------..-. .-...Y.F.T  131
Dm            ---TI----- -----NWQ.T .CF.A.LVV. LSIC..MRYT .K...M.K.. MY.SRAPGSR V.L..ML.V- -.V..VL--- ------.SWN K-.G.Y.F.G  169
ci            ---K.----- -DSKTRN--T SYLVSSTL.. LLAVLVIRYL .K...C.Q.. MF..R---.K ..LK..L.AV -SYLQLL--- ------CYFT Q-NVM.LLVC  169


Hsa1C         SLPRQPVPSV QDTVRKYLES VRPILSDEDF DWTAVLAQEF LRLQASLLQW YLRLKSWWAS NYVSDWWEEF VYLRSRNPLM VNSNYYMMDF LYVTPTPLQA  269
SSc1C         ........A. .......... ...V.CE... E.ISA..R.. .K........ ..Q..Y.... .......... ......S... .......... .N.....V..  269
Mmu1C         A........A .E........ ...V.G.DA. .RATA..ND. ...H.PR..L ..Q....CT. .......... ......GS.- I..T...... ..........  268
Hsa1A         ....L...A. K...NR..Q. ...LMKE... KRMTA...D. AVGLGPR... ..K......T .........Y I...G.G... ......A..L ..IL..HI..  269
Mmu1A         ....L...A. K...SR.... ...LMKEG.. QRMTA...D. AVNLGPK... ..K......T .........Y I...G.G.I. ......A.EM ..I...HI..  269
Mdo1A         ....L...A. K...NR.... .Q.L.NKDN. QRMKG..ED. STNLGPR... ..K......T .........Y I...G.S.I. ......A..L ..IL..TI..  269
Gga1A         ....L...A. K...NR.... ...LMN..E. KRMEG..KD. AFNLGPR... ..K......T .........Y I...G.G.I. .....FA... .HLS..TI..  269
Fpe1A         ....L...A. N...NR.... ...LMD..E. RRMEG..KD. AFNLGPR... ..K......T .........Y I...G.G.I. .....FA... ..LS..T...  269
Aca1A         ....L..... KN..NR.... .H.LMNE.Q. KRMEA.GKD. ATNLGPK... ..K......A .........Y I...G.G.I. .....FA... ..F...SV..  269
Xtr1A         ....L...P. K...KR..D. .K.LMDK.K. ERMEG..KD. ANNLGPR... ..K......T .........Y I...G.G.I. ......A... ...L..HI..  270
Lch1A         ....L...A. K..MTR.... ...LMD..Q. RRMTK..KD. ELKLGRR... ..K......T .........Y I...G.G.I. ......A..L ..........  269
Ler1A         ....L...T. K..M...... ...L.N.KE. QRMQA..KD. ELKVGPR... ..K......T .........Y I...G.G.I. ......A..Y ..IV...V..  269
Xtr1C         V...L...G. KE..QR..D. ...LMN..EY KRMTG..KD. EVNL.PR... ..K......A .........Y I...G.G.I. ......A... .......V..  269
Ler1C         ....L...P. ...L.RF... ...LMN.... RR.KA..KD. E.NL.PR... ..I....... .........Y ...QG.E.I. ......A... ......TS..  269
Cmi1C         ....L...TI ...MQR.... ...LMN..E. HRMEA..KD. EVKLGPR... ..K......T .........Y ....G.E.I. ......A... .......I..  269
Aca1C         ....L...AL K..MQ..... I..LTT.AE. QRM.A..RD. EQTLGPR... ..K....... .........Y ....G.G... ......A... I......V..  269
Lch1C         C...L...PI AK.MQR.... .H.LMD..K. KRMTA..KD. EVNL.PR... ..K....... .........Y I...G.G.I. ......G... I.AS..YI.T  268
Tni1A         ....L...DI S..C.RH... ..ALMD..Q. ERMTA.TKD. EKNLGPR... ..K....... .........Y I...G.G.I. ......A... ...I..SI..  270
Tru1A         ....L...A. S..C.R.... .H.LMDE.R. ERMTA..KD. EKNLGPR... ..K....... .........Y I...G.S.I. ......A... ...F..SI..  270
Oni1A         ....L....I K..CER.... ...LMD.QQ. ERMKG.T.D. EKNLGPR... ..K....... .........Y I...G.G.I. ......A... ...F..SI..  270
Gac1A         ....L....I K..CKR.... ...LME..QY QRMEG.TKD. EKNLGPR... ..K....... .........Y I...G.G.I. ......A... ...F..SI..  270
Dre1Aa        ....L...P. K....R.... A..LMD..QY KRMEG..KD. EKNLGPK... ..K.....T. .........Y I...G.S.I. ......A... ...N..S...  270
Tfu1A2        ....L...P. EH..KR.... ...LMD..QY KRMEA..KD. QSNLGPK... ..K..AF... ........DY ..V.G.G.I. ......A... ...F..H..V  270
Dre1Ab        ...HLF.... KE.T.R.... .Q.L....EH QRMQR..LD. E.NLGPK... ..K......T .........Y I...G.G.I. ......V... ..AF..NI..  271
Tfu1A1        ...NL.L... K..MKR.... ...L.D.TEY KKMEE..SD. QKTL.PK.H. ..K......T .........Y I.......I. ......A... .H.L..H...  271
Ola1Ca        ...NL...AI K...KR.... ...LMN.GEY ERMTK..T.. ESSLGNR... ..K..AL... .........Y ....G.S.I. ......G... .......I..  269
Oni1Cb        ...NL...TI K...KR.... ...LMD.KEY ERMTK..A.. ESSLGNR... ..K..AL... .........Y ....G.G.I. ......G... .......I..  269
Ame1C         ...NL...AI K...KR.... ...LK..SE. ERMTN..K.. EDSLG.R... ..K..AL... .........Y I...G.G.I. ......G... .......I..  269
Gac1C         ...NL...LI K...NRH... ...LMD.TEY ERMTS.SED. ESGLGKR... ..K..AL..T .........Y ....G.S.I. ......G... M......I..  271
Tru1C         ...NL...A. K...KR.... ...LMD.AQY EHVTK..A.. ESSLGNR... ..K..AL.VT .........Y ....G...I. ......V... .......I..  269
Tni1C         ...NL...A. K...KR.... ...LMD.AQY ERV.K..A.. ESSLGNR... ..K..AL.VT .........Y ....G.S.I. ......V... .......I..  269
Ola1Cb        ...HL...A. R..LTR.... ...L.T.PEY KRMTD..N.. ESSLGNR..R ..K..AL..T .........Y I...G.G.I. ......G... ......SV..  271
Oni1Ca        ...HL...AI ....SR.... ...L.T.LE. KRMTD..N.. ESNLGNR..R ..K..AL..T .........Y ......G.I. ......G... ......SV..  269
Gac1C         ...HL...PI K...SR..T. A..L.T.PE. ERMTK..GQ. EANLGNR..R .....AL..T .........Y I...G.G.I. ......G... .......V..  270
Tri1C         ...HL...AI K..LSR..R. ...L.N.LEY KRMSE..SD. EKNLGNR..R ..K..AL..T .........Y I...G.G.I. ......G... ......SV..  271
Dre1C         ...HL...PI K..LER.... .K.L.DLDG. QRMRR.TS.. EKSLGNR..R .....AL..T .........Y ......S.I. ......G... .......N..  269
Dre1B         ...HL....I D..I.R.... ...L.D..QY KQMETV.ND. KKDP.PK..K H.K......T .........Y I...G.D.I. ....F.T..L ...I..YR..  269
Oni1B         ....L..... D..IHR.... ...L.DN.QY NKMEL..SD. KENK.AQ..R C.I......T .........Y I...G.S.I. ....F.I..L ......HR..  269
Gac1B         ....L..... D..IHR.... .H.L.NSDQY .QMER..ND. KDSK.AQ..R ..I.....GT .........Y I...G.S.I. ....F.I..L ..I...HR..  269
Ola1B         ....L...R. D..I.R.... ...L.DK.QY SQMET..ND. KESK..Q..R ..I......T .......... I...G.G.I. ....F.I..L ..I...HR..  269
Tru1B         ....L..... D..IHR.... ...L.VSDEY .QMVT..K.. KDSK.AQ..R ..I......T .........Y I.....S.I. ....F.I..L ..I...HR..  269
Tni1B         ....L..... D..IHR.... ...L.VSGEY NQMVA..N.. KDSE.AQ..R ..I......T .........Y I.....S.I. ....F.I..L ......HR..  271
Hsa1B         ...KL...R. SA.IQR.... ...L.D..EY YRMEL..K.. QDKT.PR..K ..V....... .........Y I...G.S... ......V..L VLIKN.DV..  271
Mmu1B         ...KL..... PA.IHR..D. ...L.D..AY YRMET..K.. QDKT.PR..K ..V......T .........Y ......S... ......A... VLIKN.NV..  271
Mdo1B         ...KL...K. AA..HR.... ...L.D..QY YRMEM..KD. QEKT.PR..K ..V......T .........Y I...G.S..V ......V... VFTQH.NI..  271
Sha1B         ...KL...K. SA.IKR.... ...L.D..EY YRMEM..KD. QERT.PR..K ..V......T .........Y I.....G.I. ......V... VLTPH.EV..  271
Aca1B         ...KL...R. A..IQR.... ...L.DE.R. LDMEA..LD. QQRL-IR..K ..L......T .........Y I...G.S.I. ......V... ..T...H...  270
Fpe1B         ...KL...P. EA.ITR.... ...LMD..KY SKMEA..K.. KEKT.PR..K ..I.....TT ........QY I..HG.S... ......A... .....SHI..  269
Xtr1B         ...KL...PL E..IER..Q. ...L.D.DK. SEMKI..E.. QKDLGRK..K ..H......L .........Y I...G.G.I. ......A..Y .....STN..  269
LchB          C..HL..... E..LHR.... ...L.N.LQY KRMEA.TIQ. KHQT.PR..K ..L......T .........Y .......T.I. ......A..L ..II.SSV..  231
Dm            ....L.L... K..MTR..R. ...L.D..NY TRMER..K.. EQTIGKK... ..I.....ST .........Y ....G.S..C ....F.GT.A IFMNL.DK..  269
ci            LITKTNIIK. EGLGI..YKE LKRFIYEKSY EKNVLRSLH. TSN------K L.ITNPDFI. IHS.NR..QY ...AG.G.I. ......G..L ..HN...V..  263


Hsa1C         ARAGNAVHAL LLYRHRLNRQ EIPPT-L--L MG-MRPLCSA QYEKIFNTTR IPGVQK--DY IRHLHDSQHV AVFHRGRFFR MGTHSRNSLL SPRALEQQFQ  363
SSc1C         .......... .......... ..F..-.--. ..-....... .......... ....HR--.H .H..R..R.. .......... V....QSG.. ..........  363
Mmu1C         ........T. .....L.... ..S..-.--. ..-....... ...RM..... ....E.--.H L...Q..R.. .......... V....P.G.. ..........  362
Hsa1A         ......I..I ....RK.D.E ..K.I-R--. L.STI..... .W.RM...S. ...EET--.T .Q.MR..K.I V.Y....Y.K VWLYHDGR.. K..EM...M.  364
Mmu1A         .....TI..I ....RTVD.E .LK.I-R--. L.STI..... .W.RL...S. ...EET--.T .Q.VK..R.I V.Y....Y.K VWLYHDGR.. R..E....M.  364
Mdo1A         ......I.SI ....KK.D.E .LK.I-.--. L.STV..... .W.RM...S. ...EET--.T .Q.VK..R.I V.Y....Y.K VWLYHDGR.. K..EI...M.  364
Gga1A         .....II..I ....KK.D.. ..K.I-.--. ..STV..... .W.RM...S. ...EES--.T LQ.VK..K.I V.Y.K..Y.K VWLYHDGR.. K..EI...I.  364
Fpe1A         .....VI..I ....KK.D.. ..K.I-.--. ..STV..... .W.RM...S. ....ES--.V LQ.VK..K.I V.Y.K..Y.K VWLYHDGR.. K..EI...I.  364
```

```
Aca1A      ......I..I ....RK.D.. Q.Q.I-.--. ..STV..... .W.RM...S. ...EET--.T .Q.IK..K.I V.Y.K.C.YK VWLYYDGR.. K..EI...M.  364
Xtr1A      ......I..I ....RK.D.E ..K.I-Q--. ..STV..... .W.RMY..S. ...EET--.T .Q.VK..K.I V.Y.K..Y.K VWLYHDGR.. K..EI.H.M.  365
Lch1A      .....TI..I ....RK.D.E ..K.L-M--I QN-TI.M..S ...RM...S. ....ET--.T LQ.MR..K.I V.Y.K..Y.K VWLYHDGR.. K..EI...M.  363
Ler1A      .....TI..M ....RK.D.E ..H.L-M--- -.-QI.M..S ...RM..SS. ...LET--.T MQ..K..K.. V.Y.K..Y.K VWLYHSGR.. ..CEIQL.M.  361
Xtr1C      ...A.T...I ....RKVT.E .LK.L-M--I QD-CL.M... ...HM..... ....DT--.T .Q.YA..K.I V.Y.K..... VWVYQGGR.. N.KE..L...  363
Ler1C      .....L.... ....R..A.E QVQ.S-T--. P.FPI..... .W.RM..S.. L..EET--.R LQ.QA..K.. M.Y.K..Y.K VWLYQSGR.Q ..SE.QK...  364
Cmi1C      .....LT... ....RK.T.E ..K.S-T--. P.LPV....S .W.RM..S.. T..QET--.H LQ.QT..K.I V.Y.K..Y.K VWVYQGGR.. R..E..V...  364
Aca1C      .....L.Y.M MM..RK.VQE ..K.M-.--I Q.-CL.M... .W.RM..... ...MEG--.S .Q..Q..R.I ..Y.A..... VLLYHNGRM. R..E.QA...  363
Lch1C      ......IY.C .Q..RKVT.E .LK.L-.--I QD-CL.M... .H.HM...S. ...IET--.T LQ..T..K.I V.Y.K..... VWVYHGGRM. K..E..I.IE  362
Tni1A      ......I..I M...RK.D.A Q.K.L-M--. QN-TI.M... ...RM..... V...ET--.T LQ.TNETK.I V.Y.K....K VWMFYDGR.. L..EI...ME  364
Tru1A      ......I..I M...RK.D.A Q.K.IY.--. AN-KV..... .W.RM..... V...ET--.T LQ.TIETK.I V.Y.K....K VWVFYDGR.. L..EI...ME  365
Oni1A      ......I.SI M...RK.D.A Q.K.L-M--. LH-TI.M... ...RM..... V...ET--.T LQ.VNE.K.I V.Y.K..Y.K VWMFYDGR.. L..EI...ME  364
Gac1A      ......I..I M...RK.D.A Q.K.IY.--. AN-KV..... .W.PM..... V..LET--.I LQ.VN..K.I ..Y.K...K VWMFYDGR.. L..EI...MA  365
Dre1Aa     .....SI..M MM..RK.D.A Q.K.L-M--V LN-TI.M..S ...RM...S. V...ET--.V LQ.VNE.K.I ..Y.K...YK VWMFYDGR.. L..EI...ME  364
Tfu1A2     ......I.SI M...RK.D.A Q.K.L-M--V QN-TI.M..S ...RM..... V...EE--.F .K.VNE.K.I V.Y......K VWMFYDGR.. L..EI...ME  364
Dre1Ab     .....VI..I M...RK.D.A Q.K.L-M--. QN-TI.M..S ...RM...S. ...IET--.S VQ.VS..R.I V.Y....Y.K VWMFYDGR.. L..EI...ME  365
Tfu1A1     .....TI.SI M...RK.D.A Q.K.L-.--. QN-TI.M..S ...RM...S. ...IET--.T LQ.VS..R.I V.Y.K..Y.K VSMFYDGR.. L..EI...IE  365
Ola1Ca     .....SI..F F...RK..KE ..K.S-R--I P.TVI...A. .C.R...... ...EET--.T VQ.WK..DY. ..Y.K..Y.. LRVYQAGR.. ...EI.F.I.  364
Oni1Cb     .....SI.SF F...RK..KE .LK.W-.--. RS-AV.C..Y .F.RM.D.C. ...ILTAK.T VQ.WQ..DYI V.Y.K..Y.. LRVYQAGR.. ...EI.F.I.  365
Ame1C      .....TL..V .M..R...KE ..K.S-R--I P.TFI...A. .C.R...... ...EET--.T VV.WQ..EY. ..Y....Y.. LWLYQAGRM. ...EI.Y.I.  364
Gac1C      .....SI..Y F...RK..KE .LK..-R--I P.TVI..... .C.RM..... ...EETGK.T VQ.WQ..DYI ..Y....Y.. LRMYHAGR.. ...EI.S.I.  368
Tru1C      .....TIY.M ....CK..KE ..K.P-AQW. LRSAV.C..Y .F.RM...C. ...TLT--.T .Q.WK..DFI V.Y.K..Y.Q LYVYQDGR.. C..EI.F.I.  366
Tni1C      .....TIY.M ....SK..KE ..K.P-AQW. LRSAV.C..Y .F.RM...C. ...TLT--.T VH.WN..ECI V.Y.K....Q LCVYQEGR.. C..EI.F.I.  366
Ola1Cb     .....TIT.. ....RMV..E .LT.S-R--V P.TVI...A. .C.RM..... T...ET--.V LQ.WQ..EF. ..YS...YY. LWVYRAGR.. .A.EI.H.I.  366
Oni1Ca     .....TIT.. ....RKV..E .LK.S-R--V P.TVI...A. .C.RM..... T...ET--.V LQ.WL..EF. ..Y....Y.. LWVYRAGR.. ...EI.Y.I.  364
Gac1C      .....TIT.. ....RKV..E .LK.S-R--V P.TVI...A. .C.RM..... T...ET--.V LQ.WQ..EF. ..Y....... LWVYLAGR.. ...E..H.I.  365
Tri1C      .....TIT.. ....RKV..E .LK.L-W--C ICTVI...A. .C.RM..... T..EET--.V LQ.WQ..EF. ..Y.K..... LWVYRAGR.M ...E..Y.I.  366
Dre1C      .....TIT.. F...RKV..E .LN.S-R--I P.TVI...A. .C.RM..... T..EET--.V LQ.WQ..EF. ..Y....Y.. LWVYRAGR.. ...EIQF.I.  364
Dre1B      .....V...M .Q..RK.E.G .LT.L-R--A L.-IV.M..F ...RM..... ...IET--.F VQ..K.RK.L V.Y.K..L.K VWLYYGGRH. W.SE..L...  363
Oni1B      .....V...M .Q..RK.E.G .LA.L-R--A L.-TV.M..T .M.RM..... ...IET--.F VQ..T.RK.L V...K....Q VWLYTGGRH. L.SE..T...  363
Gac1B      .....V...M .Q..RK.E.G .HA.L-R--A L.-TV.M..T .M.R...... ...IET--.I VQ..T.RK.L V.Y.K....L LWLYTGGRH. L.SE..T...  363
Ola1B      .....I...M .Q..RK.E.G .HA.L-R--A L.-TV.M..T .M.RM..... ...IET--.V VQ..S.RK.L I.Y.K....Q VWLYTGGRH. L.SE..M...  363
Tru1B      .....M...M .Q..RK.E.G .HA.L-R--A L.-TV.M..T .M.RM..... L..IET--.A VL..T.RK.L I.Y.K....Q VWLYTGGRH. L.SE..L...  363
Tni1B      .....T...M .Q..RK.E.G .HA.L-R--A L.-TV.M..T .M.RM..... ...IET--.V VQ..T.RK.L I.Y.K....Q VWLYTGGRH. L.SE..L...  365
Hsa1B      ...L..II..M IM..RK.D.E ..K.V-M--A L.-IV.M..Y .M.RM..... ...KDT--.V LQ..S..R.. ..Y.K....K LWLYEGAR.. K.QD..M...  365
Mmu1B      ...L......M IM..RK.D.E ..K.V-M--A L.-.V.M..Y .M.RM..... ...KET--.L LQ..SE.R.. ..Y.K....K VWLYEGSR.. K..D..M...  365
Mdo1B      ...L.SV...M IM..RK.D.E ..K.V-M--A L.-IV.M..Y .M.RM..... L..KDT--.V LQ..L..R.. ..Y.K...YK LWLYQGSQ.. K..D..M...  365
Sha1B      ...L..V...M IM..RK.D.E ..K.V-M--A L.-IV.M..Y .M.RM..... ...KES--.V LQ..V..R.. ..Y.K...YK VWLYQGTQ.. K..D..M...  365
Aca1B      ........SI ....R..D.E DLA.V-M--A L.-VV....Y .M.R...... ...K.A--.R LL..S..R.L ....K....K VWLYHAGK.. P..D..M...  364
Fpe1B      .....M...I .M..RK.D.G ....M-M--A L.-IV.M..Y .S.RM..... ...KET--.T LL..V..K.L ..Y.K...YK VWLYYGGQ.. Q.CD..L...  363
Xtr1B      .....VI..M ....RK.E.G L...V-M--A L.-IV.M..N .MVRM..... V...ET--.C LQ..VE.R.. C.Y.K..YY. LALYENGN.. T..Q.QA.I.  363
LchB       .....T...M ....RK.D.E ..K.M-M--A LK-LV.M..N .V.RM..... ...LET--GT VK---Q.AL. YIYNKAV.VD CMLY.LS--. VKLDIL.KQK  320
Dm         ...A.VISL. .NF.RLIEH. .LQ.I-M--V Q.-.I....W ...RT...A. V..LET--.R .I.YR..N.I V.L.K.CYYK .LIYYKGRI. R.CE.QV.IE  363
ci         S..A.I...M FAF.ST.DKE L.K.I-K--V N.-VV..... ...RV..SC. T...EA--.R .C.WS.CR.I ..Y.Q..W.K .TCYKNGV.. E.SEM.I.IE  357

Hsa1C      RILDDPSPAC PHEEHLAALT AAP----R-- GTWAQVRTSL KTQA--AEAL EAVEGAAFFV SLDAEPAGLT -------RED PAASLDAYAH ALLAGRGHDR  448
SSc1C      .......... .......... ..----.-- DM.....K.. ....--E... .......... ...S....DA AGDTPEPSG. S......... ..........  455
Mmu1C      D......... .L........ ..----.-- SM.....E.V ..H.--.T.. .......... ...S...... -------... .......... ..........  447
Hsa1A      ....NT.EPQ .G.AR..... .GD----.-- VP..RC.QAY FGRGKNKQS. D...K..... T..ETEE.YR -------S.. .DT.M.S..K S..H..CY..  451
Mmu1A      Q....T.EPQ .G.AK..... ..D----.-- VP..KC.QTY FARGKNKQS. D...K..... T..ESEQ.YR -------E.. .E..I.S..K S..H..CF..  451
Mdo1A      .......EPQ AG..K..... .GD----.-- VP..KA.QTY FSRGKNKQS. D...K..... TM.DTEQ.YS -------KK. .LT.M.S..K S..H.KCY..  451
Gga1A      .....D.EPQ AG..K..... .GD----.-- VP..KA.QAY FSRGKNKQS. D...K..... T..DDEQ.YS -------K.. .VS......K S.IH..CY..  451
Fpe1A      ...N.D.EPQ AG..K..... .GD----.-- VP..KA.QAY FSRGKNKQS. D...K..... T..DIEQ.YR -------KD. .VK......K S.IH..CY..  451
Aca1A      W....K.KPQ .G..K..... .GD----.-- VP..KA.QTY FARGKNKQS. D.I.K..... T..DTAQ.YR -------E.. .VTTMET..K S..H.KCY..  451
Xtr1A      K.I..T.SPQ .G..K..... .GD----.-- VP..KA.KAY FANGKNKQSM D...K..... T..ETEQ.YN -------K.. .VN...S..K S..H.KCY..  452
Lch1A      .....D.KPQ .G..K..... .GD----.-- VP..KA..TY FCRGKNKLS. D...K..... T..DTEQ.FR -------K.. .VT...R..K S..H.KCY..  450
Ler1A      K....Q.LPQ TG..K..... .GD----.-- VP..KA.Q.Y FS.GRNKLS. ..I.K..... T..DTEQ.FR -------K.E .IS....N..K S..H.KCY..  448
Xtr1C      N..N....PQ .G..K..... .GE----.-- TA..KA.KTY FRSGKNLQ.. DL...R..... T.QDDEE..R -------T.. .VN.....GK S..H.KCY..  450
Ler1C      Y....A.IPQ .G.KY..... .GK----.-- IP..K..K.Y FSSGKNKT.M DS...K..... T..EDTPE.F -------VDN QVK...Q..K S..H.KCY..  451
Cmi1C      ...A.T.LPQ .G........ .GN----.-- IP.GKA.K.F FSNGKNRSS. DC...K....L T..GDKP..Q -------V.. .VK......K L..H.KCY..  451
Aca1C      S..G..T.PS .G..K.P... .GE----.-- DP..RA.NAF FQTGQNEQS. SI...K..... T..TSEQ..R -------EPN .GQ......K S..H..CC..  450
Lch1C      K..A.K.SPL .G....P... .GD----.-- VP..KA.RDY FQSGLNRQS. DL...K..... ...ESEQ..K -------TD. ..K......K L..H.KCY..  449
Tni1A      K..A.Q.APQ .G..K..... .GTG---.-- TP..NA.DTY FSRGKNKQ.. D.I.K..... T..DTEQRYD -------TNN .VV...S..K C..H.KCY..  452
Tru1A      ...A.Q.EPL .G..R..... .GD----.RG TP..NA.DTY FSRGKNKQS. D.I.K..... T..DTEQCYD -------TNN .VT...S..K S..H.KCY..  454
Oni1A      ...A.K.EPL .G..R..... .GD----.-- TP..KA.E.F FSRGKNKQS. D...K....L T..DTEQRYD -------TKN .VK...I..K S..H.KCY..  451
Gac1A      ...A.TTEPM .G..K..... .GD----.-- TP..NA.ETY FSRGKNKQS. D.I.K...C. T..DTEQRFE -------SDN .DQ..VS..K S..H.KCY..  452
Dre1Aa     ...A.K.EPQ .G..F..... .GD----.-- VP..KA.SQF FIRGKNKQS. D...K..... T..DSEQRYE -------PDN .IQ....S.GK S..H.KCY..  451
Tfu1A2     ...A.T.MPQ .G..T..... .GD----.-- VP..KA..EF FSTGKNKKS. D...R..... T..DTEQRYE -------PAN .VQ...S..K S..H.KCY..  451
Dre1Ab     ...A.T.EPQ .G..T..... .GDSVCQ.-- VP..CA.NAY LRHGTNKKS. DS...K..... T..DTEQRFD -------QKN .VE...R..K S..H.KCY..  456
Tfu1A1     ...A.T.EPQ .G..K..... .GD----.-- VP..CA.DAY LR.GKNRQS. D...K..... T..DTEQRHN -------SDS ..E..RSFGK S..H.KCY..  452
Ola1Ca     ......A.PS KG.AK.G... ..D----.-- VS..EA.VKY FSSGINKRS. DVI.R..... T..D.EQ.TM -------.D. QP....N..K S..H.KCY..  451
Oni1Cb     ........PS KG.AK.G... .GD----.-- IP..KA..KY FSSGVNKRS. DCI.K..... T..D.EQ.MM -------GD. .......R..K S..H.KCY..  452
Ame1C      ........PA .G..K.G.F. .GD----.-- IP..KA.KEF FSSGVNKRS. DCI.K..... T..DDEQ.MM -------GD. ...NV.R..K S..H.KCY..  451
Gac1C      K.......PS KG.AK.G... .GD----.-- IP..KA.AKY FSSGVNKRS. DFI.K..... T..DDEQ.GV -------AD. .-TRI.S..K S..H.KCY..  454
Tru1C      .........S KG.AR.G... .GD----.-- IP..KA.AKH FNSGINKKS. DCI.K..... T..D.EQSIV -------GDN LGE...C.IK S..H.KCYN.  453
Tni1C      .......APS KG.AK.G... .GD----.-- TP..RA.AKY FSSGVNKKS. DCI.K..... T..D.EQ.IM -------GDN LRE...H.IK S..H.KCYK.  453
Ola1Cb     W.......PL .G..K.G... .GD----.-- VP...I.KEH FSSGVNKRS. DII.K..... T..D.AQ.MK -------GD. .TGN..R..K S..H.KCY..  453
Oni1Ca     .........L .G..K.G... .GD----.-- IP...M.KQY FSSGVNKRS. D.I.R..... T..D.EQ.MR -------GD. .EGN..S..K S..H.KCY..  451
Gac1C      ........PQ .G..K.G... .GESFRP.-- VP.F.M.ERH FSSGINKRS. DCI.R..... T..D.EQ.MR -------G.. ..GN..R..K S..H.KCY..  456
Tri1C      K.......PQ .G..K.G... .GDR---.-- IP.....KQY FSSGVNKRS. DVI.K....I T..D.EQ.MR -------G.. ..GN..R..K S..H.KCY..  454
Dre1C      ........PS .G..K.G... .GN----.-- TP..R..KQF FSSGVNKQS. DCI.K..... T..DQAE.MK -------G.N .SEN..R..K S..H.KCY..  451
Dre1B      .....K.EPQ .G.LK.PS.. .GN----.-- VP..RA.LKY FGEGVNRAS. ..I.T....L T..D.AH.YD -------P.N I-R...L..K S..H.KCY..  449
Oni1B      ...N.T.EPQ .G.LK..... .GY----.-- IP...A.IKY FS.GINKVS. D.I.S....L T..D..Q.YD -------PAK S-N...S..K S..H.KCY..  449
Gac1B      ...N.T.EPQ .G.LK..... .GH----.-- VP...S.IKY FG.GVNKVS. D.I.S....L T..D..Q.YD -------PAK A-K...S..K S..H.KCY..  449
Ola1B      ...N.TTEPQ .G.LK..... .GN----.-- VP...A.IKH FSHGVNKTS. D.I.S....L T..D.SQ.YD -------GVK K-N...S..K S..H.KCY..  449
Tru1B      ...N.T.EPQ QG.LK..... .GN----.Q- VP..RA.SKY FS.GLNKVS. D.I.S....L T..D..Q.YD -------QAR .-R...S..K S..H.KCY..  450
Tni1B      ...S.T.EPQ QG.LK..... .GN----.-- VP...A.AKY F..GLNKAS. D.I.S....L T..D..Q.YD -------HAR S-R...S..K S..H.KCY..  451
Hsa1B      ........PQ .G..K..... .GG----.-- VE...A.QAF FSSGKNKA.. ..I.R..... A..E.SYSYD -------P.. E-...SL.GK ...H.NCYN.  451
```

```
Mmu1B       ........PQ .G..K..... .GG----.-- VE..EA.QTF FSSGKNKMS. D.I.R..... T..EDSHCYN -------PD. E-T..SL.GK ...H.NCYN.  451
Mdo1B       ........PQ .G..K...F. .GG----.-- VQ..EA.QTY FNTGKNKAS. ..I.K..... T..E.SH..D -------P.N E-...SL.GK S..H.NCYN.  451
Sha1B       .....TC.IQ .G..K..... .GG----.-- VQ..EA.QTY FNTGKNKAS. ..I.K..... T..E.SH.YD -------P.. E-...SL.GK ...H.NCYN.  451
Aca1B       ........PE TG..R..... .GE----.-- LP..EA.EKY FSRGKNKAS. DC..R..... T..E.EH.FD -------PDK E-D...R.SK S..H.QCC..  450
Fpe1B       ........PQ .G..R..... .GE----.-- VP..EA.ARF FSHGKNKVS. D.I.R....L T..E.EH.YV -------AGK E-GCM.T..K S..H.QCY..  449
Xtr1B       Y....S..PQ .G..K..... .GN----.-- VH...A..NF FSNGINRT.. SC..R.V..I ...E.E..YN -------E.. K-S..S..SK ...H.NCYN.  449
LchB        ..KIIT.YIK YERKGSVRIS LSC----.-- IP..KA.SEF FSHGKNKIS. T.I.R....M T..D.EQAYD -------K.N .-TT..S..K S..H.KCF..  406
Dm          E..KGKATPV EG........ .WN----.-- SK..EA.NTF FSWGVNQTS. RTI.S...VL ...D..FEFD -------LAR .-EL..NFGK K..H.N.YN.  449
ci          S..N.T..P. EG........ .GE----.-- IP..KA.NTY FVDGVNKKS. H.I.K...IL V..D.EHVVS -------D.. S-...SK.GR S..H.KCYN.  443


Hsa1C       WFDKSFTLIV FSNGKLGLSV EHSWADCPIS GHMWEFTLAT ECFQLGYSTD GHCKGHPDPT LPQPQRLQWD LPDQIHSSIS ----LALRGA KILSENVDCH  544
SSc1C       ......S..I .......... ..A....... .......... ........A. .........S .......H.. ...K..L... ----...... QA.A..I...  551
Mmu1C       .......... .......... ........V. ..L....... .......A.. .......... .......... ..E..QP... ----...... .T..G.I...  543
Hsa1A       .......FV. .K...M..NA ......A..V A.L..YVMSI DSL....AE. .....DIN.N I.Y.T..... I.GECQEV.E ----TS.NT. NL.AND..F.  547
Mmu1A       .....I.FV. .K.S.I.INA ......A..V ..L..YVM.. DV......E. .....DKN.N I.K.T..... I.GECQEV.E ----TS.SS. SF.AND..L.  547
Mdo1A       ....T..F.. .K...M..NA ......A..V ..L..YVM.. D......TE. .....DTN.N I.Y.T....E I.EECQDV.E ----ES.SL. ST.AND..F.  547
Gga1A       ....T...V. .K...M..NA ......A..V ..L..NVM.. .YLE...LE. .....DTNQN I.I.TK...E I.EECQDV.E ----RS.ST. RA.ADD..FY  547
Fpe1A       ....T..... .K..RI..NA ......A..V ..L..NVM.. .YLE....E. .....DINQN I.I.TK...E I.AECQEV.E ----RS.ST. IA.ADD..FY  547
Aca1A       .......... .K...M..NT ......A..V ..L..NVMFS D.LE...TE. .....ESSSG ILM.S....E ILEECQEV.E ----RS.AV. RP.ADD..F.  547
Xtr1A       ....TMSFV. .K...M.MN. ......A..V ..L..YVM.. DKME...NE. .....DVNGN I.P.S..... I.EECQNVVE ----ES.TV. .A.ADD..F.  548
Lch1A       .....LSFV. .K...M..NS ......A..V ..L..YV... DS.....TEE .....E.K.S I.F....R.E I.EECQEV.E ----IS.KV. .A.ADD..F.  546
Ler1A       .....LSF.I .K...I..NA ......A..I ..L..YV... DQ.....TDE .N...E.N.Q IQP....... IEEPCQEV.H ----QS.SV. QQ.ADD..F.  544
Xtr1C       .......F.. .K...I..NA ......A..V ......V... D......NEE .N...QV.SN ..V......E ISEECQEV.Q ----SS.AV. QA.ADD..F.  546
Ler1C       .......F.. .A...V..NA ......A..I ..L..YA... DT.....KE. .N...D.A.N V.L....... I.KECQEV.M ----SS.KV. QT.AND..FY  547
Cmi1C       .......... .E...V..NA ......A..I ..L..YV... DS.....NDQ .....DAES. VLS....... I.EACQEV.. ----GS.KV. QS.AND..F.  547
Aca1C       .......... YR...S..NA ......A..V ..L..YC... DA.T...DAY .N...DM..N V.P..K...E I.PECEAV.M ----QSF.V. YN.ASDI.F.  546
Lch1C       .......V.. .K......NA ......A..I ..L..YV... DT..I..KP. .....EA.S. ILP....... I.AECREV.K ----MS.AV. QT.AND..F.  545
Tni1A       ......N... .K..TM..NA ......A..V ..L..QV.SM DPKN...TE. ...R.A.H.N ..G....... ISTECQQV.Q ----SS.TV. QK.ADD..S.  548
Tru1A       ......N... .K..TM..NA ......A..V ..L..HV.SM DPNN...TEE ...R.V.H.N ..G..K.... I.AECQQV.Q ----NS.TV. QN.ADD..S.  550
Oni1A       .....LNM.. YK..TM..NA ......A..V ..L..HV.SM DP-K...TE. ...V.K.H.N ..G......T I.AECQEA.E ----SS.TV. RA.ADD....  546
Gac1A       ......N..I YK..TM..NA ......A..V ..L..HV.SM DPIK...TEA .....E.H.N ..G..K.S.. I.AECQEV.Q ----SS.KV. RT.ADD..S.  548
Dre1Aa      .....LN... .K..TM..NA ......A..V ..L..QV.SS DPVR...TEE .....N.H.N M.G....... I.EECQTV.. ----SS.KV. NT.ADD..M.  547
Tfu1A2      ......N..I .K..TM..NA ..T...A..V ..L..HV.SM DPIT...TE. ...R.K.H.N ..G.L..... ISVECQ.V.R ----SS.KV. NA.ADD..M.  547
Dre1Ab      .....IN..I .K..TM..NA ......A..V ..L..QV.SM DPVK...TE. .....E.HAN ..G......N I.TECQTM.T ----NS.SV. EA.ADD..S.  552
Tfu1A1      ......N..I YK.ATI..NA ......A..I ..L..NV.S. DAIK...TD. ...A.QTHRN ..G......N I.PECQTM.A ----NS.SV. QA.ADD..MV  548
Ola1Ca      ......SV.Y YK...S.ING ......A.VV A.V..YV... DS.....NEE .....EV.AS .....K.N.E ISPECEEQ.. ----RS.AV. QA.ADD..F.  547
Oni1Cb      .......VVY YK...N.INA ......A.VL A.V..Y...N DS.....NAE .....DV..S ..R.VK.S.E I.PECEEQ.A ----QS.AV. QA.ADD..F.  548
Ame1C       ......SVVI .K...N..NA ......A..V S....YA... DS.....NEE .....DVN.S .......T.. I.K.CQEQVA ----QC.AV. QP.ADDI.F.  547
Gac1C       ......SVVY .K...M..NG ......A.VL S.A.QYV.T. D......NAE .....EV.SS ..G..K.N.E I.PECEEQ.. ----GS.AV. QA.ADD..V.  550
Tru1C       ......SVVF YK...S..NG ....G.A.VL T.L..Y.... .......NAE .....EV.AS .AE..K.N.E ISSECEEQ.. ----QS.EV. QA.AND..M.  549
Tni1C       ......SVVF YK...S..NG ....G.A.VL S.L..Y.... .......NAE .....EV.AS ..K..K.N.E I.SECEEQ.C ----QS.AL. QA.AND..M.  549
Ola1Cb      ......SIVI YK...S..NA ......A.TV A.L..Y.... DA.....TE. .....EV.RS ..P.H..S.E I.SEVQDQ.F ----SS.TL. .A.ADD....  549
Oni1Ca      ......SVVI YK...S..NA ......A.TV A.L..Y.... DA.....TE. .....EVE.S .......V.N I.AEVQAQV. ----SS.AV. QA.ADD....  547
Gac1C       ......SIVI YK...S..NA ......A.TV A.L..Y.... DA.....TE. .....DV.RS ..P....A.. I.SEVQAQA. ----SS.VV. QA.ADD....  552
Tri1C       ......SIVI YK...N..NA ......A.TV A.L..Y.... DA.H...TE. .....EVE.L ..H....L.. I.LECNTQVQ AQQCSS.AV. QA.ADD....  554
Dre1C       ......SVV. YK...N..NA ......A..V A.L....... DT.H...NS. .N.R.DV.HS ..H....S.. I.FEVQTQ.. ----ES.AV. QA.ADE....  547
Dre1B       ......N... YK...M.VNT ......S..I .....YV... D..H...TAE .....DVNK. .AP.T..... I.KACQEI.E ----GSY.I. .GIADD..F.  545
Oni1B       .........S YP...M.VN. ......A..V .....YI... D..H...TEE .....DVNKN ..H.T....Q I.NECQNV.E ----TSYLS. .QIADD..F.  545
Gac1B       .........S YP...V.VNA ......A..V .....YV... D..H...TEE .....DVNKG ..H.S....Q I.NECQEV.E ----TSYLS. .LIADD..F.  545
Ola1B       .........S YP...M.VN. ......A..V .....YV... D..H.C..EE .....DANRG ..F.T....Q ISKECQDV.E ----ASYLS. .KIADD..F.  545
Tru1B       .........S YP...M.IN. ......A..V .....YV.S. D..H...TEE .....DVNKG ..Y.S....Q I.VECK.I.E ----ASYVS. .RIADD..FY  546
Tni1B       .........S YP...M.IN. ......A.VV ......V... D..H...TEE .....DVNKG ..Y.S....Q I.VECQ.I.E ----ASYVS. .QIADD..F.  547
Hsa1B       .........S .K..Q...NA ..A...A..I ..L...V.G. DS.H...TET ...L.K.N.A .AP.T..... I.K.CQAV.E ----SSYQV. .A.ADD.ELY  547
Mmu1B       .........S CK..L...NT ......A..I ..L...V.G. DT.H...TET ...V.E.NT. ..P....P.. I.E.CREA.E ----NSYQV. .A.ADD.ELY  547
Mdo1B       ......N..S .K.A....NT ......A..V ..L...V... DA.H.D.TDA ...Q.K.NHS .AP....L.. I.EECQEL.E ----SSYQV. .T.ADD.ELY  547
Sha1B       ........VA .K......NT .A...A.VV ..L...V... DA.H.D.NES ...Q.K.NHS .AP......E I.EECQKI.E ----SSYEV. .A.ADD.ELY  547
Aca1B       ......S.V. YR.....ANA ......A..I ..L...M... DH.....CS. ...H.V.NTA ..P....T.. I.EECCNV.D ----ASYAV. RA.ADDI.F.  546
Fpe1B       ........V. YK.....ANA ......A..I ..L...A... .K.....TDR ...R.E.NTQ .AP....... I.QECRDT.E ----SSY.L. .A.ADD..FC  545
Xtr1B       ......S.V. .R......NA ......A..I ..L....... D..E...TE. .N.R.DAGSP ..P.Y..... I.PKCREV.E ----RSYVT. .AIADD..F.  545
LchB        .......F.. .K.....INT ......A.VI ..L...V... D..E.S.TES .....EMNKK ..P....... ..EECKEM.Q ----QSYKV. .A.ADD.NFC  502
Dm          ....C..VC. GT..RV.FNA ..T.S.AA.A S....NLIVD DLVSD..DET .NT..T.AFQ P.T.T..T.. .KP-CLAQ.E ----E.TIDV TK.INE.NLR  544
ci          ....T.NC.. .K..RW.INA ......A..M SYVV.EA.GF .YQS...TQ. .RV..R.TVQ PIT.H....Q .TPECQEV.E ----TS.SV. NN.ADD.HLN  539


Hsa1C       VVPFSLFGKS FIRRCHLSSD SFIQIALQLA HFRDRGQFCL TYESAMTRLF LEGRTETVRS CTREACNFVR AMEDK---EK TDPQCLALFR VAVDKHQALL  641
SSc1C       .F...H.... ..K....... ....T..... ......R... .......... .......... ......S... ...HQ---.. .......... L.........  648
Mmu1C       .F...H.... ..KC..V... ....LV.... .......... .......... .......... ......Q... ..DN.----E ..QH...... ..........  639
Hsa1A       SF..VA...G I.KK.RT.P. A.V.L..... .YK.M.K... ...AS..... R......... ..T.S.D... ..V.P---AQ .VE.R.K..K L.SE...HMY  644
Mmu1A       SF..DT...G L.KK.RT.P. A...L..... .YK.M.K... ...AS..... R......... ..T.S....L ..M.P---TT .AE.RFK..K I.CE...H.Y  644
Mdo1A       SF..DA...E L.KKSRT.P. A.V.L..... .YK.M.K... ...AS..... R......... ..M.S....L ..VNP---TE SVENK.K.L. I.AE...HMY  644
Gga1A       SFY.DV...G L.KKAKT.P. A...L..... .Y..M.K.S. ...AS..... R......... ..I.S....Q T..NP---SE SNENKMKS.. L.AT...H.Y  644
Fpe1A       SFF.DA...G L.KKAKT.P. A.V.L..... .Y..M.K.S. ...AS..... R......... ..V.S..... T...P---TE SSENK.KF.. I.AA...H.Y  644
Aca1A       SF..DT...G LMKKAKT.P. A.V.L..... .Y..M.K... ...AS..... R......... ..TQS.K..K ..D.P---SE .LEKKI...K A.ATH..L.Y  644
Xtr1A       SF..NS...G L.KKSRT.P. A.V.LS.... .Y..KEK... ...AS..... R......... ..I.S.D..L ..S.P---SQ .NEKR.Q..K E.AE...QMY  645
Lch1A       SF..DS...G L.KK.RT.P. A...L..... .YW.K.K.Y. ...AS..... R......... ..V.S....L S.V.P---KQ .NE.R.K..K I.SE...NMY  643
Ler1A       SF..DK...G V.KK.WI.P. A.V.M..... .Y..K.K... ...AS..... R......... ..SQSTQ..L ..VNS---SQ .NEEK.K... L.A....SMY  641
Xtr1C       TF..KE...G L.KK.RT.P. A......... .Y..K.K... ...AS..... R......... ..V.SSQ..K ..MNP---SQ .NEER.K... L.AE...M.Y  643
Ler1C       AF..TR...G V.KK.RT.P. A......... NY..K.T... ....S..... R......... ..V.T....T .LDG.---KH SNEE..N... I.AKN..HIS  644
Cmi1C       TF..TK...G L.KK.RT.P. A...L..... N...K.K... ....S..... R......... ..IQSS.... ....H----L SASE..K..H L.AKT..HIS  643
Aca1C       AFT.KD...G L.KK.RT.P. G...L..... ....KKK... ...AS..... R......... ..I.S....K ..M.P---TQ D.SAR.H... ..AE...N.Y  643
Lch1C       .FT.KD...G L.KK.RT.P. A......... .Y..K.K... ...AS..... R......... ..S.S.A..K ..V.A---GY .NAAR.K..K ..AE...QMY  642
Tni1A       II...E...G K.KK.RT.P. A......... .Y..K.K... ...AS..... R......... ..V.S.A... S.IRD----E .TEER.R.LK K.AE...N.Y  644
Tru1A       II...E...G K.KK.RT.P. A......... .Y..K.K... ...AS..... R......... ..M.S.A... S.IRD----E .TEER.R.LK K.AE...NMY  646
Oni1A       II..TD...G L.KK.RT.P. A......... .Y..K.K... ...AS...M. R......... ..I.T.A... ..VGD----E .REER.R.LK L.AE...N.Y  642
Gac1A       IM...D...G L.KK.RT.P. A......... .Y..KKK... ...AS..... R......... ..S.S.A... ..IKG----E .RDE..R.LK Q.AE...NMY  644
Dre1Aa      IF..ND...G L.KK.KT.P. G...L..... ....K.K... ...AS..... R......... ..N.T.A..H ..M.E---KA .REER.K.LK A.TE...N.Y  644
```

```
Tfu1A2   IF..NN...G L.KK.KT.P. G......... ....KKK... ...AS..... R......... ..M.T.D... ..M.E---KQ .REEK.R.LK L.AE...E.Y  644
Dre1Ab   II...D...G L.KK.RT.P. A...L..... .Y..K.K... ...AS..... R......... ..T.S.A... ..NSN----H .RE.K.Q.LK N.AE...QMY  648
Tfu1A1   II..QE...G L.KK.KI.P. A......... N.K.K.K... ...AS..... R......... ..I.TSA..K ..VNN----E .REKK.S.L. H.AGN..HMY  644
Ola1Ca   .LS.RD...G Q.KK.KV.P. A...MT.... YY.E..T... ...AS..... R......... ..N.SSA... .L.NGSPPSQ PADV.RR... G.SE...Q.Y  647
Oni1Cb   .FS.QE...G KVKK.RV.P. G...M..... Y..E..T... ...AS..... R......... ..N.SSA.I. .L.GG----E ATDV.KC... ..SE...L.Y  644
Ame1C    .FA.RD...G S.KKIKM.P. G...L..... FY....M... ...AS..... R......... .SS.S.A.IK .F.NG----E DVEV.RR.I. N.SE...M.Y  643
Gac1C    .FS.EE...G K.KK.RV.P. A...LT.... .Y.NQ.R... ...AS..... K......... ..N.S.A... .L.GG----E DADV.RR..H E.SE...Q.Y  646
Tru1C    ..V.RD...A KVKK.RVNP. A......... YY..QKR.S. ....S..... R......... ..N.S.A.I. .L.GG----E .TDV.RR... T.SE...R.C  645
Tni1C    ..V.RD...A KVKK.RVNP. A......... .Y..QKR.S. .......... R......... ..N.S.A.I. .L.GG----E .TDV.RR... T.CE...R.C  645
Ola1Cb   .F..RD...G K.KKLRV.P. A....S.... Y.....S... ...AS..... R......... .SNQS.A..K .L.GG----E GAD..KR... Q.SE...N.Y  645
Oni1Ca   .F..RE...G R.KK.RI.P. A....S.... YY...SG... ...AS..... R......... .SN.S.A.IK .L.SG----E .EEE.RR..Q L.SE...N.Y  643
Gac1C    .F..RD...G R.KKLRV.P. A....G.... Y.....G... ...AS..... R......... .SN.SSA... .L.T.----E AVD..RH... L.SER..N.Y  648
Tri1C    I.H.RD...G R.KKLRV.P. A.V..S.... YY....S... ...AS..... R......... ..N.SAA..K .L.NG----E DEES.RR... L.SET..N.Y  650
Dre1C    .F..RK...G L.KKMK..P. ..V.L..... YY....T... ...AS..... R......... .SN.S.A..L .L.GG----E DRE..RK.L. KTAE...N.Y  643
Dre1B    GCL.NE...G L.KK.RT.P. A...L..... QY..K.E... ....S...M. R......... ..C.STA... ....D---TT .NE.R....K Q.AE...NMY  642
Oni1B    GYL.AE...G L.KK.RT.P. A...L..... Q...QRV... ....S...M. RD........ ..S..VA... ....A---GA .NA.R..... K.AE...NMY  642
Gac1B    GYL.HE...G L.KK.RC.P. A...L..... Q...Q.V... ....S...M. RD........ ..T..VA... ....E---GA .KA.R..... K.AE...NMY  642
Ola1B    GHL.TE...G L.KK.RT.P. A...L..... Q...Q.V... ....S...M. RD........ ..S..VA... ....V---SA .NA.R.S..Q K.AE...NMY  642
Tru1B    GCL.HE...G L.KK.RT.P. A...M..... Q...Q.L... ....S...M. RD........ ..S..VA..K ...SA---DS .NA.R..... K.A....NMY  643
Tni1B    GCL.HE...G L.KK.RT.P. A...M..... Q...Q.L... ....S...M. RD........ ..S..VE... ...SA---DA .NA.R..... K.A....NMY  644
Hsa1B    CFQ.LP...G L.KK.RT.P. A.V....... ......K... ...AS...M. R......... ..S.STA..Q ..MEG---SH .KADLRD..Q K.AK...NMY  644
Mmu1B    CFQ.LP...G L.KK.RT.P. A.V....... ....K.K... ...AS...M. R......... ..N.SAA..Q ..MKG---SH KKQDLQD... K.SE...NMY  644
Mdo1B    CFH.AT...G L.KK.RT.P. A.V....... ....K.K... ...AS...M. RD........ ..T..TA..Q ..M.S---GY MK.DLQD... K.AE...L.Y  644
Sha1B    CFQ.LP...G L.KK.RS.P. A.V....... ....K.N... ...AS...M. RD........ ..A.TTA... ..T.S---GY MK.DLQD... K.AE...H.Y  644
Aca1B    SFR.VE...G R.KK.RT.P. .......... ....K.R... ...AS..... R......... ..S..TA... S.A.P---SC SSSER.E..H ..AE...H.Y  643
Fpe1B    CFQ..E...G L.KK.RT.P. A....S.... ....K.C... ...AS..... R......... ..A.STA... S.G.A---R. .VTERQR..K L.A....HMY  642
Xtr1B    CLC..D...G L.KK.RS.P. A.F....... .Y.EK.H... ...AS..... RD........ ..TQTSD..K ....P---TQ SQEKR...Y. A.AEH..LMY  642
LchB     CF..MS...G M.K.FKT.P. A......... ....K.A... ....S..... RD........ ..N.T.A..Q ..L.T---H. ..KER.S... K.SE...Y.Y  599
Dm       ILVHQDY..G .MKK.RI.P. AY..M..... YY..A.R.S. ...AS..... R........P ..I.SSAW.K ..QNP---NT .NDERVKMMQ A.C.R..LGY  641
ci       .SA.KH...G LVKKFKM.P. A...A...I. .L..K.R.S. ...AS..... R......... ..S.M.A.AK S...D---SF .NKDRY..LK K...R.INGY  636


Hsa1C    KAAMSGQGVD RHLFALYIVS RFLHLQSPFL TQ-------- ---------- ---------- --VHSEQWQL STSQIPVQQM HLFDVHNYPD YVSSGGGFGP  711
SSc1C    .......... .......... Q..R...... D.-------- ---------- ---------- --........ A......... ....I..... ..........  718
Mmu1C    ........I. ........M. .L..M..... ..-------- ---------- ---------- --.Q.Q..L. ....V....T ..I....... ..........  709
Hsa1A    RL..T.S.I. ....C..V.. KY.AVE.... KE-------- ---------- ---------- --.L..P.R. ....T.Q..V E...LE.N.E ..........  714
Mmu1A    RL..T.A.I. ....C..V.. KY.AVD.... KE-------- ---------- ---------- --.L..P.R. ....T.Q..V E...FEK... ...C......  714
Mdo1A    RL..T.A.I. ....C..V.. KY.AVD.... KE-------- ---------- ---------- --.L.DP.R. ....T.Q..V E..NLERN.E ..........  714
Gga1A    RL..T.A.I. ....C..V.. KY.SVD.... KE-------- ---------- ---------- --.L..P.R. ....T.Q.HI ---.LKKN.E ML........  711
Fpe1A    RL..T.A.I. ....C..V.. KY.AVD.... KE-------- ---------- ---------- --.L..P.R. ....T.Q.HI ---.LKKN.E ML.C......  711
Aca1A    RL..T.N.I. ....C..V.. KY.AVE.... KE-------- ---------- ---------- --.L..P.R. ....T.Q.HI ---.LNKN.G ME........  711
Xtr1A    RL..T.S.I. ....C..V.. KY.GVD.... KE-------- ---------- ---------- --.L..P.R. ....T.Q..V ...QLEKF.E N.........  715
Lch1A    RL..T.A.I. ....C..V.. KY.GVD.... KE-------- ---------- ---------- --.L..P.R. ....T.Q..V E...LN.K.E ..........  713
Ler1A    RH..T.E.I. ....C..V.. KY.GMD.... KE-------- ---------- ---------- --.L..P.K. ....T..... N.H.----.K ..........  707
Xtr1C    RH..T.G.I. ....C..V.. KY.GVD.... KE-------- ---------- ---------- --.L..P.R. ....T.I..V E...LV.H.E ...C......  713
Ler1C    RL....C.I. ....C..V.. KY.GVS.... QE-------- ---------- ---------- --.L..P.C. ....T.I..I E...LV.H.E .I.I......  714
Cmi1C    RLS.A.C.I. ....C..V.. KY.GVH.... QE-------- ---------- ---------- --.L..P.S. ....T.L..V E...LL.H.E .I.C......  713
Aca1C    RQ..T.A.I. ....C..V.. KY.G.D.... RE-------- ---------- ---------- --.L..P.R. ....T.I..L E...LQ.H.. ...C......  713
Lch1C    RL..T.A.I. ....C..V.. KY.GVD.... NE-------- ---------- ---------- --.L..P.R. ....T.I..A E...LV.H.E ...C......  712
Tni1A    RL..T...I. ....C..V.. KY.GED.A.. KE-------- ---------- ---------- --.L..P.R. ....T.L..L E...LAKH.E ..T.......  714
Tru1A    RL..T.E.I. ....C..V.. KY.GEE.A.. KE-------- ---------- ---------- --.L..P.R. ....T....V E...LVKH.E ..........  716
Oni1A    RL..T...I. ....C..V.. KY.GED.... KE-------- ---------- ---------- --.L..P.K. ....T.L..V E...LVRH.E ..........  712
Gac1A    RL..I.E.I. ....C..V.. KY.GED.... KE-------- ---------- ---------- --.L..P.R. ....T.L..V E...LVRH.E ...A......  714
Dre1Aa   .L..T.K.I. ....C..L.. KY.GED.... KE-------- ---------- ---------- --.L..P.R. ....T.L... E...LKKH.E ..T.......  714
Tfu1A2   RM..T.K.I. ..I.C..V.. KY.GDD.A.. KE-------- ---------- ---------- --.L..P.R. ....T.L..I E...LKKH.E ..T.......  714
Dre1Ab   RL..T.H.I. ....C..V.L KY.GQD.... KE-------- ---------- ---------- --.L..P.R. ....T.L..G E...LVKN.E ..T.......  718
Tfu1A1   QM..T.K.I. ....C..V.. QY.QQD.... KK-------- ---------- ---------- --.L..P.R. ....T.L..P E...LL.H.E ..........  714
Ola1Ca   RM..T.A.I. ..I.C..V.. KY.G.E.... KE-------- ---------- ---------- --.LA.P.R. ....TSI..V E...LA.H.E .I.C......  717
Oni1Cb   RL..T.A.I. ....C..V.. KY.GVE.... KE-------- ---------- ---------- --.L..P.R. ....T.I..V E...IE.H.E ...C......  714
Ame1C    RL..T.A.I. ....C..V.. KY.GIE.... KE-------- ---------- ---------- --AL..P.R. ....T.F..L E...FV.H.. .ITC......  713
Gac1C    RM..T.A.I. ....C..V.. KY.QVE.... KE-------- ---------- ---------- --.L..P.R. ....T.L-.V EM..LV.H.E ...C......  715
Tru1C    RM.ST.A.I. ....C..V.. KC.GVE.... KE-------- ---------- ---------- --LE..A.R. .S.H..Y.MI D...TV.H.E .LCY......  715
Tni1C    RM.ST.A.F. ....C..V.. KY.GVE.... KE-------- ---------- ---------- --.L..A... AS.HV.H.MI D...TV.H.E .LCY......  715
Ola1Cb   RM..T.A.I. ....C..V.. KY.GVD.... KE-------- ---------- ---------- --.L..P.R. ....T..... E...LK.H.. F..L......  715
Oni1Ca   RM..T.A.I. ....C..V.. KY.GVE.... KE-------- ---------- ---------- --.L..P.R. ....T..... N...LK.H.. FI.L......  713
Gac1C    RM..T.A.I. ....C..V.. KY.GVD.... KE-------- ---------- ---------- --.L..P.R. ....T..... E...LK.H.. FI.L......  718
Tri1C    RM..T.A.I. ....C..V.. KY.GVD.... KE-------- ---------- ---------- --.L..P.R. ....T.I..I E...LK.H.. ...L......  720
Dre1C    RL..T.S.I. ....C..V.. KY.GVE.... KE-------- ---------- ---------- --.L..P.R. ....T..... E...LV.H.E FI.L......  713
Dre1B    RL..T.A.I. ....C..... KVMGID.... K.-------- ---------- ---------- --.L..P.R. ....T.Q..L N.I.TQKF.K ...A......  712
Oni1B    RL..T.S.I. ....C..... KY.GVD.... .K-------- ---------- ---------- --.L..P.K. ....T.Q..L N.V.INKF.K ..GG......  712
Gac1B    RL..T.S.I. ....C..L.. KY.GVD.... KK-------- ---------- ---------- --.L..P.R. ....T.Q..L N.V.INKF.K ...A......  712
Ola1B    RL..T.S.I. ....C..... KY.GAD.... NK-------- ---------- ---------- --.L..P.R. ....T.Q..L N.V.INKF.K ..GA......  712
Tru1B    RL..T.S.I. ...LC...I. KY.NVD.... KKVRTTPELF PFFARFLYIF VKRLCCFLCS LQ.L..P.R. ....T.Q..L N.V.IKKF.K ..GA......  743
Tni1B    RL..T.S.I. ...LC..... KY.GVD.L.. KK-------- ---------- ---------- --.L..P.R. ....T.Q..L N.V.ISKF.K ..GA......  714
Hsa1B    RL..T.A.I. ....C..L.. KY.GVS.... AE-------- ---------- ---------- --.L..P.R. ......QS.I RM..PEQH.N HLGA......  714
Mmu1B    RL..T.A.I. ....C..... KY.GVS.... AE-------- ---------- ---------- --.L..P.S. ......QF.I CM..PKQ..N HLGA......  714
Mdo1B    RL..T.A.I. ....C..V.. KY.G.H.... A.-------- ---------- ---------- --.L..P.R. .....AQF.I .M..PEK..N HIAA......  714
Sha1B    RL..T.A.I. ....C..V.. KY.G.H.... A.-------- ---------- ---------- --.L..P.R. ....TAQF.I RM..PEK..N HLAA......  714
Aca1B    RL..T.S.L. ....C..V.. .Y.GVE.... DK-------- ---------- ---------- --.L..P.R. ....T.Q..I KM..LEAH.E CA........  713
Fpe1B    RL..T.A.I. ....C..V.. .Y.GI..... A.-------- ---------- ---------- --.L..P.R. ....T.Q..L KM..LNK... H.........  712
Xtr1B    RW..T.K.I. ....C..... KY.GTD.A.. QK-------- ---------- ---------- --.L..P.R. ....T.Q..L K...LDKF.. H..A......  712
LchB     RL..T.A.I. ....C..V.. K..GVH.... N.-------- ---------- ---------- --.LA.P.A. ....T.Q..T S..NLSK... ..........  669
Dm       QD..C.R.I. ....C..V.. KY.EVD.... NE-------- ---------- ---------- --.L..P.R. ....T.HG.T PKM.LKKH.N CI.A......  711
ci       .E..T...I. ..I.C..V.. KY.K.E.... QK-------- ---------- ---------- --.LQ.P.R. ....T.H..A MNV.LNKH.N FL.G......  706


Hsa1C    ---------- ---------- ---------- ----ADDHGY GVSYIFMGDG MITFHISSKK SSTKTDSHRL GQHIEDALLD VASLFQAGQH FKRRFRGS--  775
```

```
SSc1C      ---------- ---------- ---------- ----..E... ......T.ED T......... ...R...... ....R..... ..A...E... L...A-----  779
Mmu1C      ---------- ---------- ---------- ----.H.... .I......EN A......... ...E...... .....N.... .....RV... ...Q...---  772
Hsa1A      ---------- ---------- ---------- ----VA.D.. .....LV.EN L.N......F .CPE.----- ---------- --GIISQ.PS SDT-------  756
Mmu1A      ---------- ---------- ---------- ----VA.D.. .....IV.EN F.H......F ..PE.....F .K.LRQ.MM. IIT..GLTAN S.K-------  773
Mdo1A      ---------- ---------- ---------- ----VA.D.. .....IV.EN L.N..V...F ..PE.....F .N.LKQ.MI. IIT..GLQTN DQKH------  774
Gga1A      ---------- ---------- ---------- ----VA.D.. .....ILDEN S.H..V...F .CSE.....F .KN.QK..V. IMG...PTKN CTK-------  770
Fpe1A      ---------- ---------- ---------- ----VA.D.. .....ILDEN S.H..V...I .CSE.....F .KN.QK.MV. IMG..NLSKN CTK-------  770
Aca1A      ---------- ---------- ---------- ----VA.D.. .....IV..N L.N..V...Y .CPE.N...F .KN.KR..C. IRDM.GI.KN STK-------  770
Xtr1A      ---------- ---------- ---------- ----VA.D.. .....IV.EN L.N......F ..PE.....F .K..KQ.MI. ILA..NISTN NSNKGKK---  778
Lch1A      ---------- ---------- ---------- ----VA.D.. .....LV.EN L.NL.V...L ..LE.....F .K..RQ.MQ. ILA..NLNNK SSK-------  772
Ler1A      ---------- ---------- ---------- ----VA.D.. .....MV.EN L.NM.....F ..PE.....F .NYFQQ.M.. ILA..DLDKR TSK-------  766
Xtr1C      ---------- ---------- ---------- ----VA.D.. .....IV.EN L.N......F ..HE.....F .K..QG..R. ILA..KLH-- ----------  767
Ler1C      ---------- ---------- ---------- ----VA.D.. .....IV..H L.N....C.V ..PL..A..F .EN.SQ.M.. IIT..NLDGK KA--------  772
Cmi1C      ---------- ---------- ---------- ----VA.D.. ....FIV.EN L.N....C.V ..QY..A..F .K..VE..M. ILA..KLDEK SHK-------  772
Aca1C      ---------- ---------- ---------- ----V..N.. .....IV.ED L.N..V.C.V ..PE..A..F .LN.RN.MT. I.L..K.NKK .AA.------  773
Lch1C      ---------- ---------- ---------- ----VA.D.. .....IV.ED L.N....G.I .GLD..T..F .H..RK.... IL...AVAKK N---------  769
Tni1A      ---------- ---------- ---------- ----VA.D.. .....IL.EN L.N......R ..PE.....F .TN.KR.MV. MLN..ELDRK T.--------  772
Tru1A      VGNPFICIMV SNI------- ------LKFL SSLKVA.D.. .....IV.EN L.N......R ..PE.....F .TN.KQ.MV. MLN...LEKK A.--------  795
Oni1A      ---------- ---------- ---------- ----VA.D.. .....IL.EN H.N......R ..PE.....F .TN.RQ.M.. ILG..ELNNK AAK-------  771
Gac1A      ---------- ---------- ---------- ----VA.D.. .....IL.EN L.N......H ..PE.....F SG..RQ.M.. IKG...LEKK TQ--------  772
Dre1Aa     ---------- ---------- ---------- ----VA.D.. .....IL.ED L.N......H ..HE.....F .SN.KQ.M.. ILD...LDIK PNKK------  774
Tfu1A2     ---------- ---------- ---------- ----VA.D.. .....IL.ED L.N......Y ..IE.....F .N..KQ.M.. ILA..ELDKK TVK-------  773
Dre1Ab     ---------- ---------- ---------- ----VA.D.. ..A.VIV.EK L.N......R ..PE.....F .NN.RR.MI. MLD...LDKK DPK-------  777
Tfu1A1     ---------- ---------- ---------- ----VA.D.. ..A..IV.EN L.N....C.Y ..PEA.AQ.F .NN.KQ.M.. MLE...LETK ALK-------  773
Ola1Ca     ---------- ---------- ---------- ----VA.D.. ....N.I.EN V.N....C.H .CPS..A.KF .DQ.RK.VN. LLQ.LSPN.K REWN------  777
Oni1Cb     ---------- ---------- ---------- ----VA.D.. ....CVL.EN ..N....C.H .CPD..A.KF .TQ.RQ..R. LLK.LSPN.T EPS-------  773
Ame1C      ---------- ---------- ---------- ----VA.D.. ....SLI.EK I.S..V...H .CPD....KF .TQ.RK..H. LLQ.MTIN.S DAAGSEQ---  776
Gac1C      ---------- ---------- ---------- ----VA.D.. ....CAL.EK .LS...TC.H .CPN..ANTF .GE.RK..R. LLQ.LSPE.A EP.KAAA---  778
Tru1C      VSASLMPLPQ HC-------- ---------L CVFQVT.D.. ..C.LML.GD VL.L...C.N .CPD..ARKF .AR.RA..H. LMQ.LSPNPK EP--------  790
Tni1C      ---------- ---------- ---------- ----VT.G.. ..C.LML.GD VL.L.V.C.N .CPA..ARKF .AQ.RT..H. LIQ.LSPNPK EPLK------  775
Ola1Cb     ---------- ---------- ---------- ----VA.D.. .....IV.ED ..N..V...H .CGE.....F .AQ.AK..Q. IMAVLS.DTA SSSKG--P--  777
Oni1Ca     ---------- ---------- ---------- ----VA.D.. .....IV.ED .VN..V...Y .CSQ.....F .IQ.SK.MQ. IMA.LS.DPK TSSSSK.---  776
Gac1C      ---------- ---------- ---------- ----VA.D.. .....IV.ED ..N..V...H .CSE.....F .VQ.SK..Q. IMN.LA---- ----------  770
Tri1C      VSMKYLSLCV HYVCFLQVAF LVSVMLFYDL CSYKVA.D.. .....IT.ED ..N..V...H .CNQ.....F .AQ.SK..K. IMTVLSSSPK VQ--------  812
Dre1C      ---------- ---------- ---------- ----VA.D.. .....I..ED ..N..V.C.H .CKE....KF .CQVSQ.MV. LMT.LNPDFR DTTEANS---  776
Dre1B      ---------- ---------- ---------- ----VA.D.. .....IV.EN L........F ..PE...F.F ..N.RQ.MQ. IRA..NQKEK KM--------  770
Oni1B      ---------- ---------- ---------- ----VA.D.. .....IV.EN L........F ..PD...Y.F ....RK.M.. IQA..KPEND KTAQNAKY--  776
Gac1B      ---------- ---------- ---------- ----VA.D.. .....IV.EN L........F ..PD.N.C.F ....C..M.. IQA..KPEND KTTVE-----  773
Ola1B      ---------- ---------- ---------- ----VA.D.. .....IV.EN L........F ..PN...Y.F ..N.QR.MI. IQT..TLEHD K.TPD-----  773
Tru1B      ---------- ---------- ---------- ----VA.D.. .....IV.EN L........F .CPH.VQP.E RN.------P FT.V.CYRSN LSLPSCRTRT  803
Tni1B      ---------- ---------- ---------- ----VA.D.. .....II.EN L........F .CPH.....F ....RK.M.. IQA..K.DD. TRTME-----  775
Hsa1B      ---------- ---------- ---------- ----VA.D.. ....MIA.EN T.F......F ..SE.NAQ.F .N..RK.... I.D...VPKA YS--------  772
Mmu1B      ---------- ---------- ---------- ----VA.D.. ....MIA.EN TMF......Y ..SE.NAQ.F .N..RQ.... I.E..KISKT DS--------  772
Mdo1B      ---------- ---------- ---------- ----VA.D.. ....MIA.EN T.F..V...F ..SE.NAQ.F .N..RQ..Q. I.A..EISVP KTES------  774
Sha1B      ---------- ---------- ---------- ----VA.D.. ....MIA.EN T.F......F ..SE.NAQ.F .N..HQ..E. I.A..ELPSP KPES------  774
Aca1B      ---------- ---------- ---------- ----VA.D.. .....IA.EN LV...V...F ..PE...K.F .RN.HR.M.. I.A..D.SAK RMS-------  772
Fpe1B      ---------- ---------- ---------- ----VA.D.. .....IA.EN L....V...F ..SE.GLITR .P-------- ---------- ----------  750
Xtr1B      ---------- ---------- ---------- ----VA.D.. ......A.EN L..L.....F ..PE.N...F ..R.CQSMR. L.Q.LSPPVT VRP-------  771
LchB       ---------- ---------- ---------- ----VAED.. .....IV.ED L........Y ..PE.....F AKN.QQ.MM. LKT.QESSRV KPKN------  729
Dm         ---------- ---------- ---------- ----VA.D.. .....IA.EN L.F....A.T TCQQ..V..F A.N.SQ..S. IR.M.EQHMK DHPKPAK---  774
ci         ---------- ---------- ---------- ----VA.D.. .....ICHEN L.M..V...Y ..SE...D.F AGN..K.M.. LRN.CESIIL YS--------  764
```

```
Hsa1C      ---------- ---------- --GK--ENSR HRCGFLSRQT GASKASMTST D-F 803
SSc1C      ---------- ---------- ---------- ---------- ---------- --- 779
Mmu1C      ---------- ---------- ------...D Y.YN...CK. VDPNTPTS.. N-L 798
Hsa1A      ---------- ---------- ---------- ---------- ---------- --- 756
Mmu1A      ---------- ---------- ---------- ---------- ---------- --- 773
Mdo1A      ---------- ---------- ---------- ---------- ---------- --- 774
Gga1A      ---------- ---------- ---------- ---------- ---------- --- 770
Fpe1A      ---------- ---------- ---------- ---------- ---------- --- 770
Aca1A      ---------- ---------- ---------- ---------- ---------- --- 770
Xtr1A      ---------- ---------- ---------- ---------- ---------- --- 778
Lch1A      ---------- ---------- ---------- ---------- ---------- --- 772
Ler1A      ---------- ---------- ---------- ---------- ---------- --- 766
Xtr1C      ---------- ---------- ---------- ---------- ---------- --- 767
Ler1C      ---------- ---------- ---------- ---------- ---------- --- 772
Cmi1C      ---------- ---------- ---------- ---------- ---------- --- 772
Aca1C      ---------- ---------- ---------- ---------- ---------- --- 773
Lch1C      ---------- ---------- ---------- ---------- ---------- --- 769
Tni1A      ---------- ---------- ---------- ---------- ---------- --- 772
Tru1A      ---------- ---------- ---------- ---------- ---------- --- 795
Oni1A      ---------- ---------- ---------- ---------- ---------- --- 771
Gac1A      ---------- ---------- ---------- ---------- ---------- --- 772
Dre1Aa     ---------- ---------- ---------- ---------- ---------- --- 774
Tfu1A2     ---------- ---------- ---------- ---------- ---------- --- 773
Dre1Ab     ---------- ---------- ---------- ---------- ---------- --- 777
Tfu1A1     ---------- ---------- ---------- ---------- ---------- --- 773
Ola1Ca     ---------- ---------- ---------- ---------- ---------- --- 777
Oni1Cb     ---------- ---------- ---------- ---------- ---------- --- 773
Ame1C      ---------- ---------- ------NH.S KTQNKKE--- ---------- --L 788
Gac1C      ---------- ---------- ------QRPE LKKD------ ---------- --- 786
Tru1C      ---------- ---------- ---------- ---------- ---------- --- 790
Tni1C      ---------- ---------- ---------- ---------- ---------- --- 775
Ola1Cb     ---------- ---------- ---------- ---------- ---------- --- 777
Oni1Ca     ---------- ---------- ------RGH- ---------- ---------- --- 779
Gac1C      ---------- ---------- ---------- ---------- ---------- --- 770
Tri1C      ---------- ---------- ---------- ---------- ---------- --- 812
```

```
Dre1C       ---------- ---------- ------DEQT ---------- ---------- --- 780
Dre1B       ---------- ---------- ---------- ---------- ---------- --- 770
Oni1B       ---------- ---------- -----VHLEN GKKHI----- ---------- --- 786
Gac1B       ---------- ---------- ------QLGN GKKHM----- ---------- --- 782
Ola1B       ---------- ---------- ------CLQ- ---------- ---------- --- 776
Tru1B       ASASTFRRPC WTSRRSSQKR SK.RRWRTEN T.RWKME.S. YERYCGWNRQ KYG 856
Tni1B       ---------- ---------- ---------N GKHA------ ---------- --- 780
Hsa1B       ---------- ---------- ---------- ---------- ---------- --- 772
Mmu1B       ---------- ---------- ---------- ---------- ---------- --- 772
Mdo1B       ---------- ---------- ---------- ---------- ---------- --- 774
Sha1B       ---------- ---------- ---------- ---------- ---------- --- 774
Aca1B       ---------- ---------- ---------- ---------- ---------- --- 772
Fpe1B       ---------- ---------- ---------- ---------- ---------- --- 750
Xtr1B       ---------- ---------- ---------- ---------- ---------- --- 771
LchB        ---------- ---------- ---------- ---------- ---------- --- 729
Dm          ---------- ---------- ------SLTN GKST------ ---------- --- 782
ci          ---------- ---------- ---------- ---------- ---------- --- 764
```
