# Supplementary material for: The Origin and Diversity of Cpt1 Genes in Vertebrate Species
Source: PLoS One. 2015 Sep 30;10(9):e0138447. doi: 10.1371/journal.pone.0138447 (PMC4589379; doi:10.1371/journal.pone.0138447)
Supplement: S2 Fig — (PDF) [file pone.0138447.s002.pdf]

Supporting information 2 Fig: MAFFT Sequence alignment without gaps

```
Hsa1C    LRSWKRSRFW NDFLTGVFPA SPLSWLFLFS AIQLAWFLQL DPSLGLMEKI KELLPDWRLA AALFASCLWG ALIFLLEPHG AMSSPTKTWL ALVRIFSGRH  100
SSc1C    .......... ....I..... .......... .....C.... .......... .......... .......... .......T.. V......... ..........  100
Mmu1C    ......W.V. ....A..V.. T......... T....CL... .......... .......Q.S ..V....... .......... .......... ..........  100
Hsa1A    .H...KI..K .GII...Y.. ..S...IVVV GVMTTMYAKI .....IIA.. NRT.ETAKVS GV..GTG..V ...VMFTE.. K..RA..I.M GM.K.....K  100
Mmu1A    .H...KI..K .GII...... ..S...IVVV GVISSMHTKV .....MIA.. NRT.DTTKVS GV..GTG..V .I.MMFAE.. K..RS.RI.M .M.KV....K  100
Mdo1A    .H...KI..K .GII...Y.. ..S...IVVV GVMSTMYAKV .....IIA.. NQT.DMTKVS GI..GTG..V T..VMFAEY. KL.RG.RI.M GM.K.....K  100
Gga1A    VH...KI..K .GII...Y.. ..S...IVVV GVMSTMYAKI .....IIA.. NRT.DTTQVS GI..GTG..V ...VMFAE.. KL.AG..L.M T..KL....K  100
Fpe1A    VH...KI..K .GII...Y.. ..S...IVVV GVMSTMYAKI .....IIA.. NRT.DTTQVS GV..GTG..V ...VMFAE.. KL.AG..F.M ...KL....K  100
Aca1A    .Y...KI..K .GII...Y.. ..S...IVVV GVMSTMYAKI ......IA.. NRT.DITQVS GV..GTG..V ....MFAE.. KL.TG..I.M T..KL....K  100
Xtr1A    .H...KI..K .GI......S ..S...IVVV GVTSSMYTKV ...F.II... NNA.SATQVS GV..GTG..V S..AMF.E.. KL.AS.RI.M GM.KLL...K  100
Lch1A    .H...KI..K .GII...Y.. ..S....VVV GVVSTMYTRV .....MIAR. SQR..VTQVS GV..GTG..V ..F.MFIE.. KVPFG.RI.I R..KL..VCK  100
Ler1A    .....KR.YK .GI....Y.. ..S....VVV .VIATMYARV .....MI... RCH..TNE.S GL..STG..I ..V.MFV... KTPTSV.L.M LI.K.....K  100
Xtr1C    .....KA.VK .S.I...Y.. ..S....VVI ..MGTLYARV ...M.MI... ..H..ASQVS .L..STG..F ...MMY.Q.. K..AT..L.. ...K.....N  100
Ler1C    I....KN.IK .N.M...Y.. ..S..I.VVL TVVGTMYTRV ...M..IAML R.HI.LRQVS .M...TG..L ....MF.E.. KV..T..V.F T..K.....K  100
Cmi1C    V.A..KA.IR .NIV...Y.. ..S....VAL TVVGTMYTRV ...M.MIG.. ..H..VKQMS .L...TV..L ...YMF.E.. KA.NM.RI.F TM.K.....K  100
Aca1C    V....KNQLR .G.V...Y.. ..S....MVT ..LVTQYSR. ...M.MID.. ..H..VSM.S ..T.STV..L ...MMY.E.. K..NT..I.. ...KM.A..K  100
Lch1C    Q....KA.LK .S.I..MY.. ..S.....VI ..MATLYARV ...M.MID.. ..H..VKI.C TN.YQCGIYS EILLMF.Q.N RV.LT..I.I T..K.....K  100
Tni1A    .H...KI..K .GVM...Y.G ..AGFMIVVG SYMSNKYNR. ...M..VV.L GQYI.IGQVG GV.VGTS..V TI.MMH.R.. SV.WTSRA.M L..KV....K  100
Tru1A    .H...KI..K .GVM...Y.G ..GGFMIVVG SYMSNKYN.. ...M.FVV.L GQYI.ISQVG GV.VGTG..V TI.MMY.R.. SI.WTSRA.M L..KV....K  100
Oni1A    IH...KV..K .GIM...Y.G ..AGFSIVVG SYMANK.K.. ......FT.L GQHI.ISQVG GI.VGTS..V TI.MMQ.R.. SL.WSSRI.M V..KV....K  100
Gac1A    .H...KI..K .GVM...Y.G ..GGFMVVVV SYMSNKYQ.. ......IA.L GQHM.ISQVG GV.VGTG..V SI.MMYTS.. SVAWS.RL.M V..KV....K  100
Dre1Aa   IH...KI..K .GVM...Y.G ..SGLVVVLV GYMSTKYAKI .....ILT.L STH..VSQVG GV.VGTG..I .VT.MFNQ.. TL.LK..I.. V..KL...PK  100
Tfu1A2   .H...KI..K .GVM...Y.G ..TGL.GVVG GYLITKYANI ..T..VVA.L APH..VCQVG GI.VGTGV.T .V..MYNR.. T..IR..I.. ...KL...PK  100
Dre1Ab   IH...KI..K .GI.N..Y.G .APGFVLVLA GYLG.QY.KV ......LF.L GNYV.ISQVG GVMVGTG..I .I..MFAS.. R.TWKIRL.. VF.KV...QT  100
Tfu1A1   .H...KI..K .GIM...Y.G .APGLMLVLA GYMG.KYA.V ......VFR. GKYV.ISQVG GI.VGTG..V VI..MR.R.. SLTWK.QI.. V..KV...MK  100
Ola1Ca   V....KI.IK .SVI...Y.. ..S....VVI ..LATMYTRS ...M.IIA.. Q.H..VSQVS .V..STM..L L...MF.Q.. K..TT..I.V .........K  100
Oni1Cb   M....KV.VK .SVI...Y.. ..S....VVI ..LATMYTRS ...M..IA.. Q.H..VSQVS .V..STM..L M...MF.Q.. K..TT..I.V .........K  100
Ame1C    .....KV.LK .NVI...Y.. ..S....VVI ..LATMYTRS ...M..IA.. Q.H..ASQVS .V..STL..L S...MF.Q.. NV.TT..V.V M........Q  100
Gac1C    V....KIGLK .SVV...Y.. .LS....VVI ..LATMYTRS ...M..IA.. Q.H..RPY.S .S.AVTM..L L...MF.K.. K..NT..V.V VS.ASHPS.K  100
Tru1C    VN...KI.IK .SVIR..Y.. ..F....VVI ..LATMYTRS ...M..IA.. Q.N..VSK.S .VI.STM..L L...MF.... K..TT..V.V .........K  100
Tni1C    VH...KI.IK .SVIK..Y.. ..F....VVI ..LATMYTRS ...M..IA.. Q.N..VSK.S .VI.STM..L L...MF.... K..TT..V.V .........K  100
Ola1Cb   V....K..MR .RVIK..Y.. ..S....VAI G.LATIYM.S ...M..IAE. QQR..LSQVS .LV.STL..L S..LMF.Q.. RV.NT..V.V T.L.LL...K  100
Oni1Ca   V....K..VR .SLIK..Y.. ..S....VAI G.LATMYM.S ...M..IT.. QQH..LSQVS .LV.STL..L S..LMF.K.. RI.NT..V.V P...LL.S.K  100
Gac_0    V....K..MR .RVIK..Y.. ..S....VVI ..LATMYMRS ...M..IKM. QQH..LRQ.S .LV.STL..L S..LMF.Q.. RV.NT..V.V T.L.LL.S.K  100
Tri1C    V....K..VR .SLIK..Y.. ..S....VSI ..LATMYMRS ...M..IT.. QHH..LSQ.S .LV.STL..L S..LMF.Q.. RV.NF..V.V T.LGLL.S.K  100
Dre1C    .....K..IK .RIIK.AY.. ..S....IVI ..LATMYM.S ...M..IA.. Q.H..LSQ.S .L..STL..M S..LMF.Q.. H..TK..V.A T..KLL.S.K  100
Dre1B    VT...KI..K .SV....Y.. ..S....VVI ..MSTMYARI ...M.TID.. .TS..VSQ.S .I...TG..L SV..IF.S.. K..YS..V.. S..KLL...R  100
Oni1B    VTA..KIQ.K .GV.A..Y.. ..S...IVVI .MMSSLYIHI .....M.DA. ..N..YR..S .I...TG..L F..YIF.S.. K..TS..V.. C..KM....R  100
Gac1B    VTA..KIQ.K .GV.A..Y.. ..S...IVVI .MMSSLYIRI .....MI.AL Q.N..HR..S .I...TG..L F..YIF.S.. K..RS..V.. S..KM....R  100
Ola1B    .TV..KIQ.K .SV....Y.. ..S...IVVI .MMSSLYTNT .L...MIDA. ..N..HR..S .I..GTG..L F..YIF.S.. K...S..L.. Y..KM....R  100
Tru1B    .TA..KIQ.K .GV.A..Y.. ..S...IVVI .MMSSLYTRV .....MI.AM ..N..YR..S .I...TG..L F..YIF.S.. K..TS..V.. T..KM....R  100
Tni1B    .TA.RKIQ.K .GV.A..Y.. ..S...IVVI .MMSSLYI.V .L...MI.AM ..N..YR.VS .I...TG..L F..YIF.S.. K..TS..V.. T..KM....R  100
Hsa1B    IN...KI.IK .GI.R..Y.G ..T...VVIM .TVGSS.CNV .I....VSC. QRC..QG..S M.I.STGV.V TG..MF.M.. KT.NL.RI.A MCI.LL.S..  100
Mmu1B    IN...KI.IK .GI.R..Y.G ..T...VVVM .TVGSNYCKV .I.M..VDC. QRC..ERE.S MVI.STGV.A TG..MF.M.S KT.HA..I.A IC..LL.S.R  100
Mdo1B    IN...KI.IK .GI.R..Y.G ..T...VVVM TTMGSSYCNV .L.M.MICC. RKYI.EG.IS VGI.STGV.V TG..MF.L.. QT.RT..I.A IC..LL.N.R  100
Sha1B    IN...KI.IK .GI.R..Y.G ..T...VVVM .TMGSSYCNV .I.M.MICH. RKYI.EG.IS VGI.STGV.V TG..MF.M.. QT.RI..I.A IC..LL.N.R  100
Aca1B    VS...KA.LK .SI......G T.SG..AVVA VTVG.TYFGI .V.V..IFR. QKR..NS..S .LI.S.GA.M LGVLMF.... KTRPS..I.A SM..VM....  100
Fpe1B    IS...KV.AK .S.....Y.. ..S..MVVVM .TAGSFYC.V .....MIAR. RHC..ES.VS TVI.STGA.L SAVLMF.... K..RS.RI.V ..MKVL.I.K  100
Xtr1B    .VACRKITLK SSV.S..Y.. ..ST.FAVVA MTLGSLYGK. ......IT.. NSI..GK..S .VI.S.GV.V SG.LMF...K KT.MK..I.A GCMK.M.S.Q  100
LchB     .MN.LHVFVQ .SL....Y.. ..S...VVVV .TIGTRYVKM .....IIDY. RSAI.RS..S .T...TGV.V TG.LMF.... STRIR.RI.A T..K......  100
Dm       V....KA.AR .GVRN..Y.. HIQ.LWLISA IALGLH.AGY QAPFN.TNR. LVH..SNWT. CF.A.LVV.L SIC.MY.SRS RV.L..ML.V .V..VL.SNK  100
ci       V...RKT..R .RV.S....V KYT.LVG.TA IVL..S..SY .ITW.FKNNR TNII.PRNTS YLVSSTL..L LAVLMF..R. K..LK..L.A SYLQLLCYTQ  100

Hsa1C    PMLFSYQRSL PRQPVPSVQD TVRKYLESVR PILSDEDFDW TAVLAQEFLR LWYLRLKSWW ASNYVSDWWE EFVYLRSRNP LVNSNYYMMD FLYVTPTPLQ  200
SSc1C    .......... ......A... .......... .V.CE...E. ISA..R...K ....Q..Y.. .......... .........S .......... ..N.....V.  200
Mmu1C    .R...F..A. .......A.E .......... .V.G.DA..R ATA..ND... .L..Q....C T......... ........GS .I..T..... ..........  200
Hsa1A    ...Y.F.T.. ..L...A.K. ..NR..Q... .LMKE...KR MTA...D.AV G...K..... .T........ .YI...G.G. .......A.. L..IL..HI.  200
Mmu1A    ...Y.F.T.. ..L...A.K. ..SR...... .LMKEG..QR MTA...D.AV N...K..... .T........ .YI...G.G. I......A.E M..I...HI.  200
Mdo1A    ...Y.F.T.. ..L...A.K. ..NR.....Q .L.NKDN.QR MKG..ED.ST N...K..... .T........ .YI...G.S. I......A.. L..IL..TI.  200
Gga1A    ...Y.F.T.. ..L...A.K. ..NR...... .LMN..E.KR MEG..KD.AF N...K..... .T........ .YI...G.G. I.....FA.. ..HLS..TI.  200
Fpe1A    ...Y.F.T.. ..L...A.N. ..NR...... .LMD..E.RR MEG..KD.AF N...K..... .T........ .YI...G.G. I.....FA.. ...LS..T..  200
Aca1A    ...Y.F.T.. ..L.....KN ..NR.....H .LMNE.Q.KR MEA.GKD.AT N...K..... .A........ .YI...G.G. I.....FA.. ...F...SV.  200
Xtr1A    ...Y.F.T.. ..L...P.K. ..KR..D..K .LMDK.K.ER MEG..KD.AN N...K..... .T........ .YI...G.G. I......A.. ....L..HI.  200
Lch1A    ...Y.F.T.. ..L...A.K. .MTR...... .LMD..Q.RR MTK..KD.EL K...K..... .T........ .YI...G.G. I......A.. L.........  200
Ler1A    .LTY.F.T.. ..L...T.K. .M........ .L.N.KE.QR MQA..KD.EL K...K..... .T........ .YI...G.G. I......A.. Y..IV...V.  200
Xtr1C    ...Y...AV. ..L...G.KE ..QR..D... .LMN..EYKR MTG..KD.EV N...K..... .A........ .YI...G.G. I......A.. ........V.  200
Ler1C    ...Y.F.N.. ..L...P... .L.RF..... .LMN....RR .KA..KD.E. N...I..... .......... .Y...QG.E. I......A.. .......TS.  200
Cmi1C    ...Y.F.A.. ..L...TI.. .MQR...... .LMN..E.HR MEA..KD.EV K...K..... .T........ .Y....G.E. I......A.. ........I.  200
Aca1C    ...Y...A.. ..L...ALK. .MQ.....I. .LTT.AE.QR M.A..RD.EQ T...K..... .......... .Y....G.G. .......A.. .I......V.  200
Lch1C    ...Y...AC. ..L...PIAK .MQR.....H .LMD..K.KR MTA..KD.EV N...K..... .......... .YI...G.G. I......G.. .I.AS..YI.  200
Tni1A    ...Y.F.N.. ..L...DIS. .C.RH..... ALMD..Q.ER MTA.TKD.EK N...K..... .......... .YI...G.G. I......A.. ....I..SI.  200
Tru1A    ...Y.F.N.. ..L...A.S. .C.R.....H .LMDE.R.ER MTA..KD.EK N...K..... .......... .YI...G.S. I......A.. ....F..SI.  200
Oni1A    ...Y.F.N.. ..L....IK. .CER...... .LMD.QQ.ER MKG.T.D.EK N...K..... .......... .YI...G.G. I......A.. ....F..SI.  200
Gac1A    ...Y.F.N.. ..L....IK. .CKR...... .LME..QYQR MEG.TKD.EK N...K..... .......... .YI...G.G. I......A.. ....F..SI.  200
Dre1Aa   ...Y.F.S.. ..L...P.K. ...R....A. .LMD..QYKR MEG..KD.EK N...K..... T......... .YI...G.S. I......A.. ....N..S..  200
Tfu1A2   ...Y.F.S.. ..L...P.EH ..KR...... .LMD..QYKR MEA..KD.QS N...K..AF. .......... DY..V.G.G. I......A.. ....F..H..  200
Dre1Ab   ...Y.F.N.. .HLF....KE .T.R.....Q .L....EHQR MQR..LD.E. N...K..... .T........ .YI...G.G. I......V.. ...AF..NI.  200
Tfu1A1   .N.Y.F.T.. .NL.L...K. .MKR...... .L.D.TEYKK MEE..SD.QK T...K..... .T........ .YI....... I......A.. ..H.L..H..  200
Ola1Ca   .L.Y...G.. .NL...AIK. ..KR...... .LMN.GEYER MTK..T..ES S...K..AL. .......... .Y....G.S. I......G.. ........I.  200
Oni1Cb   .L.Y...G.. .NL...TIK. ..KR...... .LMD.KEYER MTK..A..ES S...K..AL. .......... .Y....G.G. I......G.. ........I.  200
Ame1C    .L.Y...G.. .NL...AIK. ..KR...... .LK..SE.ER MTN..K..ED S...K..AL. .......... .YI...G.G. I......G.. ........I.  200
Gac1C    .L.Y...G.. .NL...LIK. ..NRH..... .LMD.TEYER MTS.SED.ES G...K..AL. .T........ .Y....G.S. I......G.. .M......I.  200
Tru1C    .R.Y...A.. .NL...A.K. ..KR...... .LMD.AQYEH VTK..A..ES S...K..AL. VT........ .Y....G... I......V.. ........I.  200
Tni1C    .R.Y...G.. .NL...A.K. ..KR...... .LMD.AQYER V.K..A..ES S...K..AL. VT........ .Y....G.S. I......V.. ........I.  200
```

```
Ola1Cb    .L.Y...T.. .HL...A.R. .LTR...... .L.T.PEYKR MTD..N..ES SR..K..AL. .T........ .YI...G.G. I......G.. .......SV.  200
Oni1Ca    .L.Y...S.. .HL...AI.. ..SR...... .L.T.LE.KR MTD..N..ES NR..K..AL. .T........ .Y......G. I......G.. .......SV.  200
Gac_0     .L.Y...T.. .HL...PIK. ..SR..T.A. .L.T.PE.ER MTK..GQ.EA NR.....AL. .T........ .YI...G.G. I......G.. ........V.  200
Tri1C     .L.Y...T.. .HL...AIK. .LSR..R... .L.N.LEYKR MSE..SD.EK NR..K..AL. .T........ .YI...G.G. I......G.. .......SV.  200
Dre1C     .L.Y...T.. .HL...PIK. .LER.....K .L.DLDG.QR MRR.TS..EK SR.....AL. .T........ .Y......S. I......G.. ........N.  200
Dre1B     .L.Y.F.G.. .HL....ID. .I.R...... .L.D..QYKQ METV.ND.KK DKH.K..... .T........ .YI...G.D. I....F.T.. L...I..YR.  200
Oni1B     .L.Y.F.A.. ..L.....D. .IHR...... .L.DN.QYNK MEL..SD.KE NRC.I..... .T........ .YI...G.S. I....F.I.. L......HR.  200
Gac1B     .L.Y.F.A.. ..L.....D. .IHR.....H .L.NSDQY.Q MER..ND.KD SR..I..... GT........ .YI...G.S. I....F.I.. L..I...HR.  200
Ola1B     .L.Y.F.A.. ..L...R.D. .I.R...... .L.DK.QYSQ MET..ND.KE SR..I..... .T........ ..I...G.G. I....F.I.. L..I...HR.  200
Tru1B     .L.Y.F.A.. ..L.....D. .IHR...... .L.VSDEY.Q MVT..K..KD SR..I..... .T........ .YI.....S. I....F.I.. L..I...HR.  200
Tni1B     .L.Y.F.A.. ..L.....D. .IHR...... .L.VSGEYNQ MVA..N..KD SR..I..... .T........ .YI.....S. I....F.I.. L......HR.  200
Hsa1B     ...Y.F.T.. .KL...R.SA .IQR...... .L.D..EYYR MEL..K..QD KK..V..... .......... .YI...G.S. .......V.. LVLIKN.DV.  200
Mmu1B     ...Y.F.T.. .KL.....PA .IHR..D... .L.D..AYYR MET..K..QD KK..V..... .T........ .Y......S. .......A.. .VLIKN.NV.  200
Mdo1B     ...Y.F.T.. .KL...K.AA ..HR...... .L.D..QYYR MEM..KD.QE KK..V..... .T........ .YI...G.S. .......V.. .VFTQH.NI.  200
Sha1B     ...YTF.T.. .KL...K.SA .IKR...... .L.D..EYYR MEM..KD.QE RK..V..... .T........ .YI.....G. I......V.. .VLTPH.EV.  200
Aca1B     ...Y.F.T.. .KL...R.A. .IQR...... .L.DE.R.LD MEA..LD.QQ RK..L..... .T........ .YI...G.S. I......V.. ...T...H..  200
Fpe1B     .L.Y.F.T.. .KL...P.EA .ITR...... .LMD..KYSK MEA..K..KE KK..I..... TT........ QYI..HG.S. .......A.. ......SHI.  200
Xtr1B     ...Y.F.M.. .KL...PLE. .IER..Q... .L.D.DK.SE MKI..E..QK DK..H..... .L........ .YI...G.G. I......A.. Y.....STN.  200
LchB      ...Y.F.TC. .HL.....E. .LHR...... .L.N.LQYKR MEA.TIQ.KH QK..L..... .T........ .Y......T. I......A.. L..II.SSV.  200
Dm        .G.Y.F.G.. ..L.L...K. .MTR..R... .L.D..NYTR MER..K..EQ T...I..... ST........ .Y....G.S. .....F.GT. AIFMNL.DK.  200
ci        NVM.LLVCLI TKTNIIK.EG LGI..YKELK RFIYEKSYEK NVLRSLH.TS NKL.ITNPDF I.IHS.NR.. QY...AG.G. I......G.. L..HN...V.  200

Hsa1C     AARAGNAVHA LLLYRHRLNR QEIPPTLGMR PLCSAQYEKI FNTTRIPGVQ KDYIRDSQHV AVFHRGRFFR MGTHSRNLSP RALEQQFQRI LDDPSPACPH  300
SSc1C     .......... .......... ...F...... .......... .........H R.H.H..R.. .......... V....QS... .......... ..........  300
Mmu1C     .........T ......L... ...S...... ........RM .........E ..HL...R.. .......... V....P.... ........D. .........L  300
Hsa1A     .......I.. I....RK.D. E..K.IR.TI ......W.RM ...S....EE T.T.Q..K.I V.Y....Y.K VWLYHDG.K. .EM...M... ..NT.EPQ.G  300
Mmu1A     ......TI.. I....RTVD. E.LK.IR.TI ......W.RL ..S....EE T.T.Q..R.I V.Y....Y.K VWLYHDG.R. .E....M.Q. ...T.EPQ.G  300
Mdo1A     .......I.S I....KK.D. E.LK.I..TV ......W.RM ...S....EE T.T.Q..R.I V.Y....Y.K VWLYHDG.K. .EI...M... .....EPQAG  300
Gga1A     ......II.. I....KK.D. ...K.I..TV ......W.RM ...S....EE S.TLQ..K.I V.Y.K..Y.K VWLYHDG.K. .EI...I... ...D.EPQAG  300
Fpe1A     ......VI.. I....KK.D. ...K.I..TV ......W.RM ...S.....E S.VLQ..K.I V.Y.K..Y.K VWLYHDG.K. .EI...I... .N.D.EPQAG  300
Aca1A     .......I.. I....RK.D. .Q.Q.I..TV ......W.RM ...S....EE T.T.Q..K.I V.Y.K.C.YK VWLYYDG.K. .EI...M.W. ...K.KPQ.G  300
Xtr1A     .......I.. I....RK.D. E..K.IQ.TV ......W.RM Y..S....EE T.T.Q..K.I V.Y.K..Y.K VWLYHDG.K. .EI.H.M.K. I..T.SPQ.G  300
Lch1A     ......TI.. I....RK.D. E..K.LMNTI .M..S...RM ...S.....E T.TLQ..K.I V.Y.K..Y.K VWLYHDG.K. .EI...M... ...D.KPQ.G  300
Ler1A     ......TI.. M....RK.D. E..H.LM.QI .M..S...RM ..SS.....LE T.TMQ..K.. V.Y.K..Y.K VWLYHSG... CEIQL.M.K. ...Q.LPQTG  300
Xtr1C     ....A.T... I....RKVT. E.LK.LMDCL .M......HM .........D T.T.Q..K.I V.Y.K..... VWVYQGG.N. KE..L...N. .N....PQ.G  300
Ler1C     ......L... .....R..A. EQVQ.ST.PI ......W.RM ..S..L..EE T.RLQ..K.. M.Y.K..Y.K VWLYQSGQ.. SE.QK...Y. ...A.IPQ.G  300
Cmi1C     ......LT.. .....RK.T. E..K.ST.PV ....S.W.RM ..S..T..QE T.HLQ..K.I V.Y.K..Y.K VWVYQGG.R. .E..V..... .A.T.LPQ.G  300
Aca1C     ......L.Y. MMM..RK.VQ E..K.M..CL .M....W.RM ........ME G.S.Q..R.I ..Y.A..... VLLYHNG.R. .E.QA...S. .G..T.PS.G  300
Lch1C     T......IY. C.Q..RKVT. E.LK.L.DCL .M....H.HM ...S....IE T.TLQ..K.I V.Y.K..... VWVYHGG.K. .E..I.IEK. .A.K.SPL.G  300
Tni1A     .......I.. IM...RK.D. AQ.K.LMNTI .M......RM .....V...E T.TLQETK.I V.Y.K....K VWMFYDG.L. .EI...MEK. .A.Q.APQ.G  300
Tru1A     .......I.. IM...RK.D. AQ.K.I.NKV ......W.RM .....V...E T.TLQETK.I V.Y.K....K VWVFYDG.L. .EI...ME.. .A.Q.EPL.G  300
Oni1A     .......I.S IM...RK.D. AQ.K.LMHTI .M......RM .....V...E T.TLQE.K.I V.Y.K..Y.K VWMFYDG.L. .EI...ME.. .A.K.EPL.G  300
Gac1A     .......I.. IM...RK.D. AQ.K.I.NKV ......W.PM .....V..LE T.ILQ..K.I ..Y.K....K VWMFYDG.L. .EI...MA.. .A.TTEPM.G  300
Dre1Aa    ......SI.. MMM..RK.D. AQ.K.LMNTI .M..S...RM ...S.V...E T.VLQE.K.I V.Y.K...YK VWMFYDG.L. .EI...ME.. .A.K.EPQ.G  300
Tfu1A2    V......I.S IM...RK.D. AQ.K.LMNTI .M..S...RM .....V...E E.F.KE.K.I V.Y......K VWMFYDG.L. .EI...ME.. .A.T.MPQ.G  300
Dre1Ab    ......VI.. IM...RK.D. AQ.K.LMNTI .M..S...RM ...S....IE T.SVQ..R.I V.Y....Y.K VWMFYDG.L. .EI...ME.. .A.T.EPQ.G  300
Tfu1A1    ......TI.S IM...RK.D. AQ.K.L.NTI .M..S...RM ...S....IE T.T.Q..R.I V.Y.K..Y.K VSMFYDG.L. .EI...IE.. .A.T.EPQ.G  300
Ola1Ca    ......SI.. FF...RK..K E..K.SR.VI ...A..C.R. ........EE T.TVQ..DY. ..Y.K..Y.. LRVYQAG... .EI.F.I... ....A.PSKG  300
Oni1Cb    ......SI.S FF...RK..K E.LK.W.SAV .C..Y.F.RM .D.C....IL T.TVQ..DYI V.Y.K..Y.. LRVYQAG... .EI.F.I... ......PSKG  300
Ame1C     ......TL.. V.M..R...K E..K.SR.FI ...A..C.R. ........EE T.TVV..EY. ..Y....Y.. LWLYQAG... .EI.Y.I... ......PA.G  300
Gac1C     ......SI.. YF...RK..K E.LK..R.VI ......C.RM ........EE T.TVQ..DYI ..Y....Y.. LRMYHAG... .EI.S.I.K. ......PSKG  300
Tru1C     ......TIY. M....CK..K E..K.PARAV .C..Y.F.RM ...C....TL T.T.Q..DFI V.Y.K..Y.Q LYVYQDG.C. .EI.F.I... .......SKG  300
Tni1C     ......TIY. M....SK..K E..K.PARAV .C..Y.F.RM ...C....TL T.TVH..ECI V.Y.K....Q LCVYQEG.C. .EI.F.I... .....APSKG  300
Ola1Cb    ......TIT. .....RMV.. E.LT.SR.VI ...A..C.RM .....T...E T.VLQ..EF. ..YS...YY. LWVYRAG..A .EI.H.I.W. ......PL.G  300
Oni1Ca    ......TIT. .....RKV.. E.LK.SR.VI ...A..C.RM .....T...E T.VLQ..EF. ..Y....Y.. LWVYRAG... .EI.Y.I... .......L.G  300
Gac_0     ......TIT. .....RKV.. E.LK.SR.VI ...A..C.RM .....T...E T.VLQ..EF. ..Y....... LWVYLAG... .E..H.I... ......PQ.G  300
Tri1C     ......TIT. .....RKV.. E.LK.LWCVI ...A..C.RM .....T..EE T.VLQ..EF. ..Y.K..... LWVYRAGM.. .E..Y.I.K. ......PQ.G  300
Dre1C     ......TIT. .F...RKV.. E.LN.SR.VI ...A..C.RM .....T..EE T.VLQ..EF. ..Y....Y.. LWVYRAG... .EIQF.I... ......PS.G  300
Dre1B     ......V... M.Q..RK.E. G.LT.LR.IV .M..F...RM ........IE T.FVQ.RK.L V.Y....L.K VWLYYGG.W. SE..L..... ...K.EPQ.G  300
Oni1B     ......V... M.Q..RK.E. G.LA.LR.TV .M..T.M.RM ........IE T.FVQ.RK.L V..K....Q VWLYTGG.L. SE..T..... .N.T.EPQ.G  300
Gac1B     ......V... M.Q..RK.E. G.HA.LR.TV .M..T.M.R. ........IE T.IVQ.RK.L V.Y.K....L LWLYTGG.L. SE..T..... .N.T.EPQ.G  300
Ola1B     ......I... M.Q..RK.E. G.HA.LR.TV .M..T.M.RM ........IE T.VVQ.RK.L I.Y.K....Q VWLYTGG.L. SE..M..... .N.TTEPQ.G  300
Tru1B     ......M... M.Q..RK.E. G.HA.LR.TV .M..T.M.RM .....L..IE T.AVL.RK.L I.Y.K....Q VWLYTGG.L. SE..L..... .N.T.EPQQG  300
Tni1B     ......T... M.Q..RK.E. G.HA.LR.TV .M..T.M.RM ........IE T.VVQ.RK.L I.Y.K....Q VWLYTGG.L. SE..L..... .S.T.EPQQG  300
Hsa1B     ...L..II.. MIM..RK.D. E..K.VM.IV .M..Y.M.RM ........KD T.VLQ..R.. ..Y.K....K LWLYEGA.K. QD..M..... ......PQ.G  300
Mmu1B     ...L...... MIM..RK.D. E..K.VM..V .M..Y.M.RM ........KE T.LLQE.R.. ..Y.K....K VWLYEGS.K. .D..M..... ......PQ.G  300
Mdo1B     ...L.SV... MIM..RK.D. E..K.VM.IV .M..Y.M.RM .....L..KD T.VLQ..R.. ..Y.K...YK LWLYQGS.K. .D..M..... ......PQ.G  300
Sha1B     ...L..V... MIM..RK.D. E..K.VM.IV .M..Y.M.RM ........KE S.VLQ..R.. ..Y.K...YK VWLYQGT.K. .D..M..... ...TC.IQ.G  300
Aca1B     .........S I....R..D. EDLA.VM.VV ....Y.M.R. .........K. A.RLL..R.L ....K....K VWLYHAG.P. .D..M..... ......PETG  300
Fpe1B     ......M... I.M..RK.D. G....MM.IV .M..Y.S.RM ........KE T.TLL..K.L ..Y.K...YK VWLYYGG.Q. CD..L..... ......PQ.G  300
Xtr1B     ......VI.. M....RK.E. GL...VM.IV .M..N.MVRM .....V...E T.CLQE.R.. C.Y.K..YY. LALYENG.T. .Q.QA.I.Y. ...S..PQ.G  300
LchB      ......T... M....RK.D. E..K.MMKLV .M..N.V.RM ........LE TGTVKQ.AL. YIYNKAV.VD CMLY.LS.VK LDIL.KQK.. KIIT.YIKYE  300
Dm        ....A.VISL ..NF.RLIEH ..LQ.IM..I ....W...RT ...A.V..LE T.R.I..N.I V.L.K.CYYK .LIYYKG.R. CE.QV.IEE. .KGKATPVEG  300
ci        .S..A.I... MFAF.ST.DK EL.K.IK.VV ........RV ..SC.T...E A.R.C.CR.I ..Y.Q..W.K .TCYKNG.E. SEM.I.IES. .N.T..P.EG  300

Hsa1C     EEHLAALTAA PRGTWAQVRT SLKTQAAEAL EAVEGAAFFV SLDAEPAGLT REDPASLDAY AHALLAGRGH DRWFDKSFTL IVFSNGKLGL SVEHSWADCP  400
SSc1C     .......... ..DM.....K ......E... .......... ...S....DA SG.S...... .......... ........S. .I........ ....A.....  400
Mmu1C     .......... ..SM.....E .V..H..T.. .......... ...S...... .......... .......... .......... .......... ..........  400
Hsa1A     .AR......G D.VP..RC.Q AYFGRGKQS. D...K..... T..ETEE.YR S...T.M.S. .KS..H..CY .........F V..K...M.. NA......A.  400
Mmu1A     .AK....... D.VP..KC.Q TYFARGKQS. D...K..... T..ESEQ.YR E.....I.S. .KS..H..CF .......I.F V..K.S.I.I NA......A.  400
Mdo1A     ..K......G D.VP..KA.Q TYFSRGKQS. D...K..... TM.DTEQ.YS KK..T.M.S. .KS..H.KCY ......T..F ...K...M.. NA......A.  400
Gga1A     ..K......G D.VP..KA.Q AYFSRGKQS. D...K..... T..DDEQ.YS K...S..... .KS.IH..CY ......T... V..K...M.. NA......A.  400
Fpe1A     ..K......G D.VP..KA.Q AYFSRGKQS. D...K..... T..DIEQ.YR KD..K..... .KS.IH..CY ......T... ...K..RI.. NA......A.  400
Aca1A     ..K......G D.VP..KA.Q TYFARGKQS. D.I.K..... T..DTAQ.YR E...TTMET. .KS..H.KCY .......... ...K...M.. NT......A.  400
Xtr1A     ..K......G D.VP..KA.K AYFANGKQSM D...K..... T..ETEQ.YN K...N...S. .KS..H.KCY ......TMSF V..K...M.M N.......A.  400
Lch1A     ..K......G D.VP..KA.. TYFCRGKLS. D...K..... T..DTEQ.FR K...T...R. .KS..H.KCY .......LSF V..K...M.. NS......A.  400
Ler1A     ..K......G D.VP..KA.Q .YFS.GKLS. ..I.K..... T..DTEQ.FR K.E.S...N. .KS..H.KCY .......LSF .I.K...I.. NA......A.  400
Xtr1C     ..K......G E.TA..KA.K TYFRSGLQ.. DL..R..... T.QDDEE..R T...N..... GKS..H.KCY .........F ...K...I.. NA......A.  400
```

```
Ler1C      .KY.....G K.IP..K..K .YFSSGKT.M DS..K..... T..EDTPE.F VDNQK...Q. .KS..H.KCY .........F ...A...V.. NA......A.  400
Cmi1C      ........G N.IP.GKA.K .FFSNGRSS. DC..K....L T..GDKP..Q V...K..... .KL..H.KCY .......... ...E...V.. NA......A.  400
Aca1C      ..K.P....G E.DP..RA.N AFFQTGEQS. SI..K..... T..TSEQ..R EPN.Q..... .KS..H..CC .......... ..YR...S.. NA......A.  400
Lch1C      ....P....G D.VP..KA.R DYFQSGRQS. DL..K..... ...ESEQ..K TD..K..... .KL..H.KCY .......... V..K...... NA......A.  400
Tni1A      ..K......G T.TP..NA.D TYFSRGKQ.. D.I.K..... T..DTEQRYD TNN.V...S. .KC..H.KCY ........N. ...K..TM.. NA......A.  400
Tru1A      ..R......G D.TP..NA.D TYFSRGKQS. D.I.K..... T..DTEQCYD TNN.T...S. .KS..H.KCY ........N. ...K..TM.. NA......A.  400
Oni1A      ..R......G D.TP..KA.E .FFSRGKQS. D...K....L T..DTEQRYD TKN.K...I. .KS..H.KCY .......LNM ..YK..TM.. NA......A.  400
Gac1A      ..K......G D.TP..NA.E TYFSRGKQS. D.I.K...C. T..DTEQRFE SDN.Q..VS. .KS..H.KCY ........N. .IYK..TM.. NA......A.  400
Dre1Aa     ..F......G D.VP..KA.S QFFIRGKQS. D...K..... T..DSEQRYE PDN.Q...S. GKS..H.KCY .......LN. ...K..TM.. NA......A.  400
Tfu1A2     ..T......G D.VP..KA.. EFFSTGRKS. D...R..... T..DTEQRYE PDN.Q...S. .KS..H.KCY ........N. .I.K..TM.. NA..T...A.  400
Dre1Ab     ..T......G D.VP..CA.N AYLRHGKKS. DS..K..... T..DTEQRFD QKN.E...R. .KS..H.KCY .......IN. .I.K..TM.. NA......A.  400
Tfu1A1     ..K......G D.VP..CA.D AYLR.GRQS. D...K..... T..DTEQRHN SDS.E..RSF GKS..H.KCY ........N. .IYK.ATI.. NA......A.  400
Ola1Ca     .AK.G..... D.VS..EA.V KYFSSGKRS. DVI.R..... T..D.EQ.TM .D.Q....N. .KS..H.KCY ........SV .YYK...S.I NG......A.  400
Oni1Cb     .AK.G....G D.IP..KA.. KYFSSGKRS. DCI.K..... T..D.EQ.MM GD......R. .KS..H.KCY .........V VYYK...N.I NA......A.  400
Ame1C      ..K.G.F..G D.IP..KA.K EFFSSGKRS. DCI.K..... T..DDEQ.MM GD...NV.R. .KS..H.KCY ........SV VI.K...N.. NA......A.  400
Gac1C      .AK.G....G D.IP..KA.A KYFSSGKRS. DFI.K..... T..DDEQ.GV AD..TRI.S. .KS..H.KCY ........SV VY.K...M.. NG......A.  400
Tru1C      .AR.G....G D.IP..KA.A KHFNSGKKS. DCI.K..... T..D.EQSIV GDNLE...C. IKS..H.KCY N.......SV VFYK...S.. NG....G.A.  400
Tni1C      .AK.G....G D.TP..RA.A KYFSSGKKS. DCI.K..... T..D.EQ.IM GDNLE...H. IKS..H.KCY K.......SV VFYK...S.. NG....G.A.  400
Ola1Cb     ..K.G....G D.VP...I.K EHFSSGKRS. DII.K..... T..D.AQ.MK GD..GN..R. .KS..H.KCY ........SI VIYK...S.. NA......A.  400
Oni1Ca     ..K.G....G D.IP...M.K QYFSSGKRS. D.I.R..... T..D.EQ.MR GD..GN..S. .KS..H.KCY ........SV VIYK...S.. NA......A.  400
Gac_0      ..K.G....G E.VP.F.M.E RHFSSGKRS. DCI.R..... T..D.EQ.MR G...GN..R. .KS..H.KCY ........SI VIYK...S.. NA......A.  400
Tri1C      ..K.G....G D.IP.....K QYFSSGKRS. DVI.K....I T..D.EQ.MR G...GN..R. .KS..H.KCY ........SI VIYK...N.. NA......A.  400
Dre1C      ..K.G....G N.TP..R..K QFFSSGKQS. DCI.K..... T..DQAE.MK G.N.EN..R. .KS..H.KCY ........SV V.YK...N.. NA......A.  400
Dre1B      .LK.PS...G N.VP..RA.L KYFGEGRAS. ..I.T....L T..D.AH.YD P.NIR...L. .KS..H.KCY ........N. ..YK...M.V NT......S.  400
Oni1B      .LK......G Y.IP...A.I KYFS.GKVS. D.I.S....L T..D..Q.YD PAKSN...S. .KS..H.KCY .......... .SYP...M.V N.......A.  400
Gac1B      .LK......G H.VP...S.I KYFG.GKVS. D.I.S....L T..D..Q.YD PAKAK...S. .KS..H.KCY .......... .SYP...V.V NA......A.  400
Ola1B      .LK......G N.VP...A.I KHFSHGKTS. D.I.S....L T..D.SQ.YD GVKKN...S. .KS..H.KCY .......... .SYP...M.V N.......A.  400
Tru1B      .LK......G N.VP..RA.S KYFS.GKVS. D.I.S....L T..D..Q.YD QAR.R...S. .KS..H.KCY .......... .SYP...M.I N.......A.  400
Tni1B      .LK......G N.VP...A.A KYF..GKAS. D.I.S....L T..D..Q.YD HARSR...S. .KS..H.KCY .......... .SYP...M.I N.......A.  400
Hsa1B      ..K......G G.VE...A.Q AFFSSGKA.. ..I.R..... A..E.SYSYD P..E...SL. GK...H.NCY N......... .S.K..Q... NA..A...A.  400
Mmu1B      ..K......G G.VE..EA.Q TFFSSGKMS. D.I.R..... T..EDSHCYN PD.ET..SL. GK...H.NCY N......... .SCK..L... NT......A.  400
Mdo1B      ..K...F..G G.VQ..EA.Q TYFNTGKAS. ..I.K..... T..E.SH..D P.NE...SL. GKS..H.NCY N.......N. .S.K.A.... NT......A.  400
Sha1B      ..K......G G.VQ..EA.Q TYFNTGKAS. ..I.K..... T..E.SH.YD P..E...SL. GK...H.NCY N......... VA.K...... NT..A...A.  400
Aca1B      ..R......G E.LP..EA.E KYFSRGKAS. DC..R..... T..E.EH.FD PDKED...R. SKS..H.QCC ........S. V.YR.....A NA......A.  400
Fpe1B      ..R......G E.VP..EA.A RFFSHGKVS. D.I.R....L T..E.EH.YV AGKEGCM.T. .KS..H.QCY .......... V.YK.....A NA......A.  400
Xtr1B      ..K......G N.VH...A.. NFFSNGRT.. SC..R.V..I ...E.E..YN E..KS..S.. SK...H.NCY N.......S. V..R...... NA......A.  400
LchB       RKGSVRISLS C.IP..KA.S EFFSHGKIS. T.I.R....M T..D.EQAYD K.N.TT..S. .KS..H.KCF .........F ...K.....I NT......A.  400
Dm         ........W N.SK..EA.N TFFSWGQTS. RTI.S...VL ...D..FEFD LAR.EL..NF GKK..H.N.Y N.....C..V C.GT..RV.F NA..T.S.AA  400
ci         ........G E.IP..KA.N TYFVDGKKS. H.I.K...IL V..D.EHVVS D..S...SK. GRS..H.KCY N.....T.NC ...K..RW.I NA......A.  400

Hsa1C      ISGHMWEFTL ATECQLGYST DGHCKGHPDP TLPQPQRLQW DLPDIHSSIS LALRGAKILS ENVDCHVVPF SLFGKSFIRR CHLSSDSFIQ IALQLAHFRD  500
SSc1C      .......... .........A .......... S.......H. ......L... ......QA.A ..I....F.. .H......K. .......... T.........  500
Mmu1C      V...L..... ........A. .......... .......... ...E.QP... .......T.. G.I....F.. .H......KC ..V....... LV........  500
Hsa1A      .VA.L..YVM SIDS....AE ......DIN. NI.Y.T.... .I.GCQEV.E TS.NT.NL.A ND..F.SF.. VA...GI.KK .RT.P.A.V. L......YK.  500
Mmu1A      .V..L..YVM ..DV.....E ......DKN. NI.K.T.... .I.GCQEV.E TS.SS.SF.A ND..L.SF.. DT...GL.KK .RT.P.A... L......YK.  500
Mdo1A      .V..L..YVM ..D.....TE ......DTN. NI.Y.T.... EI.ECQDV.E ES.SL.ST.A ND..F.SF.. DA...EL.KK SRT.P.A.V. L......YK.  500
Gga1A      .V..L..NVM ...YE...LE ......DTNQ NI.I.TK... EI.ECQDV.E RS.ST.RA.A DD..FYSFY. DV...GL.KK AKT.P.A... L......Y..  500
Fpe1A      .V..L..NVM ...YE....E ......DINQ NI.I.TK... EI.ACQEV.E RS.ST.IA.A DD..FYSFF. DA...GL.KK AKT.P.A.V. L......Y..  500
Aca1A      .V..L..NVM FSD.E...TE ......ESSS GILM.S.... EILECQEV.E RS.AV.RP.A DD..F.SF.. DT...GLMKK AKT.P.A.V. L......Y..  500
Xtr1A      .V..L..YVM ..DKE...NE ......DVNG NI.P.S.... .I.ECQNVVE ES.TV..A.A DD..F.SF.. NS...GL.KK SRT.P.A.V. LS.....Y..  500
Lch1A      .V..L..YV. ..DS....TE E.....E.K. SI.F....R. EI.ECQEV.E IS.KV..A.A DD..F.SF.. DS...GL.KK .RT.P.A... L......YW.  500
Ler1A      .I..L..YV. ..DQ....TD E.N...E.N. QIQP...... .IEECQEV.H QS.SV.QQ.A DD..F.SF.. DK...GV.KK .WI.P.A.V. M......Y..  500
Xtr1C      .V......V. ..D.....NE E.N...QV.S N..V...... EISECQEV.Q SS.AV.QA.A DD..F.TF.. KE...GL.KK .RT.P.A... .......Y..  500
Ler1C      .I..L..YA. ..DT....KE ..N...D.A. NV.L...... .I.KCQEV.M SS.KV.QT.A ND..FYAF.. TR...GV.KK .RT.P.A... ......NY..  500
Cmi1C      .I..L..YV. ..DS....ND Q.....DAES .VLS...... .I.ECQEV.. GS.KV.QS.A ND..F.TF.. TK...GL.KK .RT.P.A... L.....N...  500
Aca1C      .V..L..YC. ..DAT...DA Y.N...DM.. NV.P..K... EI.PCEAV.M QSF.V.YN.A SDI.F.AFT. KD...GL.KK .RT.P.G... L.........  500
Lch1C      .I..L..YV. ..DT.I..KP ......EA.S .ILP...... .I.ACREV.K MS.AV.QT.A ND..F..FT. KD...GL.KK .RT.P.A... .......Y..  500
Tni1A      .V..L..QV. SMDPN...TE ....R.A.H. N..G...... .ISTCQQV.Q SS.TV.QK.A DD..S.II.. .E...GK.KK .RT.P.A... .......Y..  500
Tru1A      .V..L..HV. SMDPN...TE E...R.V.H. N..G..K... .I.ACQQV.Q NS.TV.QN.A DD..S.II.. .E...GK.KK .RT.P.A... .......Y..  500
Oni1A      .V..L..HV. SMDPK...TE ....V.K.H. N..G...... TI.ACQEA.E SS.TV.RA.A DD....II.. TD...GL.KK .RT.P.A... .......Y..  500
Gac1A      .V..L..HV. SMDPK...TE A.....E.H. N..G..K.S. .I.ACQEV.Q SS.KV.RT.A DD..S.IM.. .D...GL.KK .RT.P.A... .......Y..  500
Dre1Aa     .V..L..QV. SSDPR...TE E.....N.H. NM.G...... .I.ECQTV.. SS.KV.NT.A DD..M.IF.. ND...GL.KK .KT.P.G... L.........  500
Tfu1A2     .V..L..HV. SMDPT...TE ....R.K.H. N..G.L.... .ISVCQ.V.R SS.KV.NA.A DD..M.IF.. NN...GL.KK .KT.P.G... ..........  500
Dre1Ab     .V..L..QV. SMDPK...TE ......E.H. N..G...... NI.TCQTM.T NS.SV.EA.A DD..S.II.. .D...GL.KK .RT.P.A... L......Y..  500
Tfu1A1     .I..L..NV. S.DAK...TD ....A.QTHR N..G...... NI.PCQTM.A NS.SV.QA.A DD..MVII.. QE...GL.KK .KI.P.A... ......N.K.  500
Ola1Ca     VVA.V..YV. ..DS....NE E.....EV.A S.....K.N. EISPCEEQ.. RS.AV.QA.A DD..F..LS. RD...GQ.KK .KV.P.A... MT....YY.E  500
Oni1Cb     VLA.V..Y.. .NDS....NA E.....DV.. S..R.VK.S. EI.PCEEQ.A QS.AV.QA.A DD..F..FS. QE...GKVKK .RV.P.G... M.....Y..E  500
Ame1C      .VS....YA. ..DS....NE E.....DVN. S.......T. .I.KCQEVQA QC.AV.QP.A DDI.F..FA. RD...GS.KK IKM.P.G... L.....FY..  500
Gac1C      VLS.A.QYV. T.D.....NA E.....EV.S S..G..K.N. EI.PCEEQ.. GS.AV.QA.A DD..V..FS. EE...GK.KK .RV.P.A... LT.....Y.N  500
Tru1C      VLT.L..Y.. ........NA E.....EV.A S.AE..K.N. EISSCEEQ.. QS.EV.QA.A ND..M...V. RD...AKVKK .RVNP.A... ......YY..  500
Tni1C      VLS.L..Y.. ........NA E.....EV.A S..K..K.N. EI.SCEEQ.C QS.AL.QA.A ND..M...V. RD...AKVKK .RVNP.A... .......Y..  500
Ola1Cb     TVA.L..Y.. ..DA....TE ......EV.R S..P.H..S. EI.SVQDQ.F SS.TL..A.A DD.....F.. RD...GK.KK LRV.P.A... .S....Y...  500
Oni1Ca     TVA.L..Y.. ..DA....TE ......EVE. S.......V. NI.AVQAQV. SS.AV.QA.A DD.....F.. RE...GR.KK .RI.P.A... .S....YY..  500
Gac_0      TVA.L..Y.. ..DA....TE ......DV.R S..P....A. .I.SVQAQA. SS.VV.QA.A DD.....F.. RD...GR.KK LRV.P.A... .G....Y...  500
Tri1C      TVA.L..Y.. ..DAH...TE ......EVE. L..H....L. .I.LCNTQVQ SS.AV.QA.A DD....I.H. RD...GR.KK LRV.P.A.V. .S....YY..  500
Dre1C      .VA.L..... ..DTH...NS ..N.R.DV.H S..H....S. .I.FVQTQ.. ES.AV.QA.A DE.....F.. RK...GL.KK MK..P...V. L.....YY..  500
Dre1B      .I.....YV. ..D.H...TA E.....DVNK ..AP.T.... .I.KCQEI.E GSY.I..GIA DD..F.GCL. NE...GL.KK .RT.P.A... L.....QY..  500
Oni1B      .V.....YI. ..D.H...TE E.....DVNK N..H.T.... QI.NCQNV.E TSYLS..QIA DD..F.GYL. AE...GL.KK .RT.P.A... L.....Q...  500
Gac1B      .V.....YV. ..D.H...TE E.....DVNK G..H.S.... QI.NCQEV.E TSYLS..LIA DD..F.GYL. HE...GL.KK .RC.P.A... L.....Q...  500
Ola1B      .V.....YV. ..D.H.C..E E.....DANR G..F.T.... QISKCQDV.E ASYLS..KIA DD..F.GHL. TE...GL.KK .RT.P.A... L.....Q...  500
Tru1B      .V.....YV. S.D.H...TE E.....DVNK G..Y.S.... QI.VCK.I.E ASYVS..RIA DD..FYGCL. HE...GL.KK .RT.P.A... M.....Q...  500
Tni1B      VV......V. ..D.H...TE E.....DVNK G..Y.S.... QI.VCQ.I.E ASYVS..QIA DD..F.GCL. HE...GL.KK .RT.P.A... M.....Q...  500
Hsa1B      .I..L...V. G.DSH...TE T...L.K.N. A.AP.T.... .I.KCQAV.E SSYQV..A.A DD.ELYCFQ. LP...GL.KK .RT.P.A.V. ..........  500
Mmu1B      .I..L...V. G.DTH...TE T...V.E.NT ...P....P. .I.ECREA.E NSYQV..A.A DD.ELYCFQ. LP...GL.KK .RT.P.A.V. ..........  500
Mdo1B      .V..L...V. ..DAH.D.TD A...Q.K.NH S.AP....L. .I.ECQEL.E SSYQV..T.A DD.ELYCFH. AT...GL.KK .RT.P.A.V. ..........  500
Sha1B      VV..L...V. ..DAH.D.NE S...Q.K.NH S.AP...... EI.ECQKI.E SSYEV..A.A DD.ELYCFQ. LP...GL.KK .RS.P.A.V. ..........  500
Aca1B      .I..L...M. ..DH....CS ....H.V.NT A..P....T. .I.ECCNV.D ASYAV.RA.A DDI.F.SFR. VE...GR.KK .RT.P..... ..........  500
Fpe1B      .I..L...A. ...K....TD R...R.E.NT Q.AP...... .I.QCRDT.E SSY.L..A.A DD..FCCFQ. .E...GL.KK .RT.P.A... .S........  500
```

```
Xtr1B     .I..L..... ..D.E...TE ..N.R.DAGS P..P.Y.... .I.PCREV.E RSYVT..AIA DD..F.CLC. .D...GL.KK .RS.P.A.F. .......Y.E  500
LchB      VI..L...V. ..D.E.S.TE S.....EMNK K..P...... ...ECKEM.Q QSYKV..A.A DD.NFCCF.. MS...GM.K. FKT.P.A... ..........  500
Dm        .AS....NLI VDDLSD..DE T.NT..T.AF QP.T.T..T. ..KPCLAQ.E E.TIDVTK.I NE.NLRILVH QDY..G.MKK .RI.P.AY.. M.....YY..  500
ci        .MSYVV.EA. GF.YS...TQ ..RV..R.TV QPIT.H.... Q.TPCQEV.E TS.SV.NN.A DD.HLN.SA. KH...GLVKK FKM.P.A... A...I..L..  500

Hsa1C     RGQFCLTYES AMTRLFLEGR TETVRSCTRE ACNFVRAMED KKTDPQCLAL FRVAVDKHQA LLKAAMSGQG VDRHLFALYI VSRFLHLQSP FLTQVHSEQW  600
SSc1C     ..R....... .......... .......... ..S......H Q......... ..L....... .......... .......... ..Q..R.... ..D.......  600
Mmu1C     .......... .......... .......... ..Q.....DN .E..QH.... .......... .......... I......... M..L..M... .....Q.Q..  600
Hsa1A     M.K......A S.....R... ........T. S.D.....V. PQ.VE.R.K. .KL.SE...H MYRL..T.S. I.....C..V ..KY.AVE.. ..KE.L..P.  600
Mmu1A     M.K......A S.....R... ........T. S....L..M. PT.AE.RFK. .KI.CE...H .YRL..T.A. I.....C..V ..KY.AVD.. ..KE.L..P.  600
Mdo1A     M.K......A S.....R... ........M. S....L..VN PESVENK.K. L.I.AE...H MYRL..T.A. I.....C..V ..KY.AVD.. ..KE.L.DP.  600
Gga1A     M.K.S....A S.....R... ........I. S....QT..N PESNENKMKS ..L.AT...H .YRL..T.A. I.....C..V ..KY.SVD.. ..KE.L..P.  600
Fpe1A     M.K.S....A S.....R... ........V. S.....T... PESSENK.KF ..I.AA...H .YRL..T.A. I.....C..V ..KY.AVD.. ..KE.L..P.  600
Aca1A     M.K......A S.....R... ........TQ S.K..K..D. PE.LEKKI.. .KA.ATH..L .YRL..T.N. I.....C..V ..KY.AVE.. ..KE.L..P.  600
Xtr1A     KEK......A S.....R... ........I. S.D..L..S. PQ.NEKR.Q. .KE.AE...Q MYRL..T.S. I.....C..V ..KY.GVD.. ..KE.L..P.  600
Lch1A     K.K.Y....A S.....R... ........V. S....LS.V. PQ.NE.R.K. .KI.SE...N MYRL..T.A. I.....C..V ..KY.GVD.. ..KE.L..P.  600
Ler1A     K.K......A S.....R... ........SQ STQ..L..VN SQ.NEEK.K. ..L.A....S MYRH..T.E. I.....C..V ..KY.GMD.. ..KE.L..P.  600
Xtr1C     K.K......A S.....R... ........V. SSQ..K..MN PQ.NEER.K. ..L.AE...M .YRH..T.G. I.....C..V ..KY.GVD.. ..KE.L..P.  600
Ler1C     K.T....... S.....R... ........V. T....T.LDG .HSNEE..N. ..I.AKN..H ISRL....C. I.....C..V ..KY.GVS.. ..QE.L..P.  600
Cmi1C     K.K....... S.....R... ........IQ SS........ HLSASE..K. .HL.AKT..H ISRLS.A.C. I.....C..V ..KY.GVH.. ..QE.L..P.  600
Aca1C     KKK......A S.....R... ........I. S....K..M. PQD.SAR.H. ....AE...N .YRQ..T.A. I.....C..V ..KY.G.D.. ..RE.L..P.  600
Lch1C     K.K......A S.....R... ........S. S.A..K..V. AY.NAAR.K. .K..AE...Q MYRL..T.A. I.....C..V ..KY.GVD.. ..NE.L..P.  600
Tni1A     K.K......A S.....R... ........V. S.A...S.IR DE.TEER.R. LKK.AE...N .YRL..T... I.....C..V ..KY.GED.A ..KE.L..P.  600
Tru1A     K.K......A S.....R... ........M. S.A...S.IR DE.TEER.R. LKK.AE...N MYRL..T.E. I.....C..V ..KY.GEE.A ..KE.L..P.  600
Oni1A     K.K......A S...M.R... ........I. T.A.....VG DE.REER.R. LKL.AE...N .YRL..T... I.....C..V ..KY.GED.. ..KE.L..P.  600
Gac1A     KKK......A S.....R... ........S. S.A.....IK GE.RDE..R. LKQ.AE...N MYRL..I.E. I.....C..V ..KY.GED.. ..KE.L..P.  600
Dre1Aa    K.K......A S.....R... ........N. T.A..H..M. EA.REER.K. LKA.TE...N .Y.L..T.K. I.....C..L ..KY.GED.. ..KE.L..P.  600
Tfu1A2    KKK......A S.....R... ........M. T.D.....M. EQ.REEK.R. LKL.AE...E .YRM..T.K. I...I.C..V ..KY.GDD.A ..KE.L..P.  600
Dre1Ab    K.K......A S.....R... ........T. S.A.....NS NH.RE.K.Q. LKN.AE...Q MYRL..T.H. I.....C..V .LKY.GQD.. ..KE.L..P.  600
Tfu1A1    K.K......A S.....R... ........I. TSA..K..VN NE.REKK.S. L.H.AGN..H MYQM..T.K. I.....C..V ..QY.QQD.. ..KK.L..P.  600
Ola1Ca    ..T......A S.....R... ........N. SSA....L.N GQPADV.RR. ..G.SE...Q .YRM..T.A. I...I.C..V ..KY.G.E.. ..KE.LA.P.  600
Oni1Cb    ..T......A S.....R... ........N. SSA.I..L.G GEATDV.KC. ....SE...L .YRL..T.A. I.....C..V ..KY.GVE.. ..KE.L..P.  600
Ame1C     ..M......A S.....R... .......SS. S.A.IK.F.N GEDVEV.RR. I.N.SE...M .YRL..T.A. I.....C..V ..KY.GIE.. ..KEAL..P.  600
Gac1C     Q.R......A S.....K... ........N. S.A....L.G GEDADV.RR. .HE.SE...Q .YRM..T.A. I.....C..V ..KY.QVE.. ..KE.L..P.  600
Tru1C     QKR.S..... S.....R... ........N. S.A.I..L.G GE.TDV.RR. ..T.SE...R .CRM.ST.A. I.....C..V ..KC.GVE.. ..KELE..A.  600
Tni1C     QKR.S..... ......R... ........N. S.A.I..L.G GE.TDV.RR. ..T.CE...R .CRM.ST.A. F.....C..V ..KY.GVE.. ..KE.L..A.  600
Ola1Cb    ..S......A S.....R... .......SNQ S.A..K.L.G GEGAD..KR. ..Q.SE...N .YRM..T.A. I.....C..V ..KY.GVD.. ..KE.L..P.  600
Oni1Ca    .SG......A S.....R... .......SN. S.A.IK.L.S GE.EEE.RR. .QL.SE...N .YRM..T.A. I.....C..V ..KY.GVE.. ..KE.L..P.  600
Gac_0     ..G......A S.....R... .......SN. SSA....L.T .EAVD..RH. ..L.SER..N .YRM..T.A. I.....C..V ..KY.GVD.. ..KE.L..P.  600
Tri1C     ..S......A S.....R... ........N. SAA..K.L.N GEDEES.RR. ..L.SET..N .YRM..T.A. I.....C..V ..KY.GVD.. ..KE.L..P.  600
Dre1C     ..T......A S.....R... .......SN. S.A..L.L.G GEDRE..RK. L.KTAE...N .YRL..T.S. I.....C..V ..KY.GVE.. ..KE.L..P.  600
Dre1B     K.E....... S...M.R... ........C. STA....... DT.NE.R... .KQ.AE...N MYRL..T.A. I.....C... ..KVMGID.. ..K..L..P.  600
Oni1B     QRV....... S...M.RD.. ........S. .VA....... AA.NA.R... ..K.AE...N MYRL..T.S. I.....C... ..KY.GVD.. ...K.L..P.  600
Gac1B     Q.V....... S...M.RD.. ........T. .VA....... EA.KA.R... ..K.AE...N MYRL..T.S. I.....C..L ..KY.GVD.. ..KK.L..P.  600
Ola1B     Q.V....... S...M.RD.. ........S. .VA....... VA.NA.R.S. .QK.AE...N MYRL..T.S. I.....C... ..KY.GAD.. ..NK.L..P.  600
Tru1B     Q.L....... S...M.RD.. ........S. .VA..K...S AS.NA.R... ..K.A....N MYRL..T.S. I....LC... I.KY.NVD.. ..KK.L..P.  600
Tni1B     Q.L....... S...M.RD.. ........S. .VE......S AA.NA.R... ..K.A....N MYRL..T.S. I....LC... ..KY.GVD.L ..KK.L..P.  600
Hsa1B     ..K......A S...M.R... ........S. STA..Q..ME GH.KADLRD. .QK.AK...N MYRL..T.A. I.....C..L ..KY.GVS.. ..AE.L..P.  600
Mmu1B     K.K......A S...M.R... ........N. SAA..Q..MK GHKKQDLQD. ..K.SE...N MYRL..T.A. I.....C... ..KY.GVS.. ..AE.L..P.  600
Mdo1B     K.K......A S...M.RD.. ........T. .TA..Q..M. SYMK.DLQD. ..K.AE...L .YRL..T.A. I.....C..V ..KY.G.H.. ..A..L..P.  600
Sha1B     K.N......A S...M.RD.. ........A. TTA.....T. SYMK.DLQD. ..K.AE...H .YRL..T.A. I.....C..V ..KY.G.H.. ..A..L..P.  600
Aca1B     K.R......A S.....R... ........S. .TA...S.A. PCSSSER.E. .H..AE...H .YRL..T.S. L.....C..V ...Y.GVE.. ..DK.L..P.  600
Fpe1B     K.C......A S.....R... ........A. STA...S.G. A..VTERQR. .KL.A....H MYRL..T.A. I.....C..V ...Y.GI... ..A..L..P.  600
Xtr1B     K.H......A S.....RD.. ........TQ TSD..K.... PQSQEKR... Y.A.AEH..L MYRW..T.K. I.....C... ..KY.GTD.A ..QK.L..P.  600
LchB      K.A....... S.....RD.. ........N. T.A..Q..L. T...KER.S. ..K.SE...Y .YRL..T.A. I.....C..V ..K..GVH.. ..N..LA.P.  600
Dm        A.R.S....A S.....R... .....P..I. SSAW.K..QN PT.NDERVKM MQA.C.R..L GYQD..C.R. I.....C..V ..KY.EVD.. ..NE.L..P.  600
ci        K.R.S....A S.....R... ........S. M.A.AKS... DF.NKDRY.. LKK...R.IN GY.E..T... I...I.C..V ..KY.K.E.. ..QK.LQ.P.  600

Hsa1C     QLSTSQIPVQ MDPDYVSSGG GFGPADDHGY GVSYIFMGDG MITFHISSKK SSTKT 655
SSc1C     ..A....... .......... ......E... ......T.ED T......... ...R. 655
Mmu1C     L....V.... T......... .....H.... .I......EN A......... ...E. 655
Hsa1A     R.....T.Q. V..E...... ....VA.D.. .....LV.EN L.N......F .CPE. 655
Mmu1A     R.....T.Q. V......C.. ....VA.D.. .....IV.EN F.H......F ..PE. 655
Mdo1A     R.....T.Q. VN.E...... ....VA.D.. .....IV.EN L.N..V...F ..PE. 655
Gga1A     R.....T.QH I..EML.... ....VA.D.. .....ILDEN S.H..V...F .CSE. 655
Fpe1A     R.....T.QH I..EML.C.. ....VA.D.. .....ILDEN S.H..V...I .CSE. 655
Aca1A     R.....T.QH I..GME.... ....VA.D.. .....IV..N L.N..V...Y .CPE. 655
Xtr1A     R.....T.Q. VQ.EN..... ....VA.D.. .....IV.EN L.N......F ..PE. 655
Lch1A     R.....T.Q. V..E...... ....VA.D.. .....LV.EN L.NL.V...L ..LE. 655
Ler1A     K.....T... ...K...... ....VA.D.. .....MV.EN L.NM.....F ..PE. 655
Xtr1C     R.....T.I. V..E...C.. ....VA.D.. .....IV.EN L.N......F ..HE. 655
Ler1C     C.....T.I. I..E.I.I.. ....VA.D.. .....IV..H L.N....C.V ..PL. 655
Cmi1C     S.....T.L. V..E.I.C.. ....VA.D.. ....FIV.EN L.N....C.V ..QY. 655
Aca1C     R.....T.I. L......C.. ....V..N.. .....IV.ED L.N..V.C.V ..PE. 655
Lch1C     R.....T.I. A..E...C.. ....VA.D.. .....IV.ED L.N....G.I .GLD. 655
Tni1A     R.....T.L. L..E..T... ....VA.D.. .....IL.EN L.N......R ..PE. 655
Tru1A     R.....T... V..E...... ....VA.D.. .....IV.EN L.N......R ..PE. 655
Oni1A     K.....T.L. V..E...... ....VA.D.. .....IL.EN H.N......R ..PE. 655
Gac1A     R.....T.L. V..E...A.. ....VA.D.. .....IL.EN L.N......H ..PE. 655
Dre1Aa    R.....T.L. ...E..T... ....VA.D.. .....IL.ED L.N......H ..HE. 655
Tfu1A2    R.....T.L. I..E..T... ....VA.D.. .....IL.ED L.N......Y ..IE. 655
Dre1Ab    R.....T.L. G..E..T... ....VA.D.. ..A.VIV.EK L.N......R ..PE. 655
Tfu1A1    R.....T.L. P..E...... ....VA.D.. ..A..IV.EN L.N....C.Y ..PEA 655
Ola1Ca    R.....TSI. V..E.I.C.. ....VA.D.. ....N.I.EN V.N....C.H .CPS. 655
Oni1Cb    R.....T.I. V..E...C.. ....VA.D.. ....CVL.EN ..N....C.H .CPD. 655
Ame1C     R.....T.F. L....ITC.. ....VA.D.. ....SLI.EK I.S..V...H .CPD. 655
Gac1C     R.....T.L. V..E...C.. ....VA.D.. ....CAL.EK .LS...TC.H .CPN. 655
Tru1C     R..S.H..YM I..E.LCY.. ....VT.D.. ..C.LML.GD VL.L...C.N .CPD. 655
```

```
Tni1C    ..AS.HV.HM I..E.LCY.. ....VT.G.. ..C.LML.GD VL.L.V.C.N .CPA. 655
Ola1Cb   R.....T... ....F..L.. ....VA.D.. .....IV.ED ..N..V...H .CGE. 655
Oni1Ca   R.....T... ....FI.L.. ....VA.D.. .....IV.ED .VN..V...Y .CSQ. 655
Gac_0    R.....T... ....FI.L.. ....VA.D.. .....IV.ED ..N..V...H .CSE. 655
Tri1C    R.....T.I. I......L.. ....VA.D.. .....IT.ED ..N..V...H .CNQ. 655
Dre1C    R.....T... ...EFI.L.. ....VA.D.. .....I..ED ..N..V.C.H .CKE. 655
Dre1B    R.....T.Q. L..K...A.. ....VA.D.. .....IV.EN L........F ..PE. 655
Oni1B    K.....T.Q. L..K..GG.. ....VA.D.. .....IV.EN L........F ..PD. 655
Gac1B    R.....T.Q. L..K...A.. ....VA.D.. .....IV.EN L........F ..PD. 655
Ola1B    R.....T.Q. L..K..GA.. ....VA.D.. .....IV.EN L........F ..PN. 655
Tru1B    R.....T.Q. L..K..GA.. ....VA.D.. .....IV.EN L........F .CPH. 655
Tni1B    R.....T.Q. L..K..GA.. ....VA.D.. .....II.EN L........F .CPH. 655
Hsa1B    R.......Q. I..NHLGA.. ....VA.D.. ....MIA.EN T.F......F ..SE. 655
Mmu1B    S.......Q. I..NHLGA.. ....VA.D.. ....MIA.EN TMF......Y ..SE. 655
Mdo1B    R......AQ. I..NHIAA.. ....VA.D.. ....MIA.EN T.F..V...F ..SE. 655
Sha1B    R.....TAQ. I..NHLAA.. ....VA.D.. ....MIA.EN T.F......F ..SE. 655
Aca1B    R.....T.Q. I..ECA.... ....VA.D.. .....IA.EN LV...V...F ..PE. 655
Fpe1B    R.....T.Q. L...H..... ....VA.D.. .....IA.EN L....V...F ..SE. 655
Xtr1B    R.....T.Q. L...H..A.. ....VA.D.. ......A.EN L..L.....F ..PE. 655
LchB     A.....T.Q. TN........ ....VAED.. .....IV.ED L........Y ..PE. 655
Dm       R.....T.H. T..NCI.A.. ....VA.D.. .....IA.EN L.F....A.T TCQQ. 655
ci       R.....T.H. A..NFL.G.. ....VA.D.. .....ICHEN L.M..V...Y ..SE. 655
```
