# Supplementary material for: The Origin and Diversity of Cpt1 Genes in Vertebrate Species
Source: PLoS One. 2015 Sep 30;10(9):e0138447. doi: 10.1371/journal.pone.0138447 (PMC4589379; doi:10.1371/journal.pone.0138447)
Supplement: S3 Fig — —Maximum likelihood phylogeny (gap alignment) using aBayes for branch support (Figure A), Maximum likelihood phylogeny with 1000 bootstraps replicates (Figure B), Bayesian Phylogenetic analysis (Figure C) and Phylogenetic analysis using Neighbor-Joining method (Figure D). (PDF) [file pone.0138447.s003.pdf]

Supporting information 3 Figs: Supporting Phylogenetic analysis

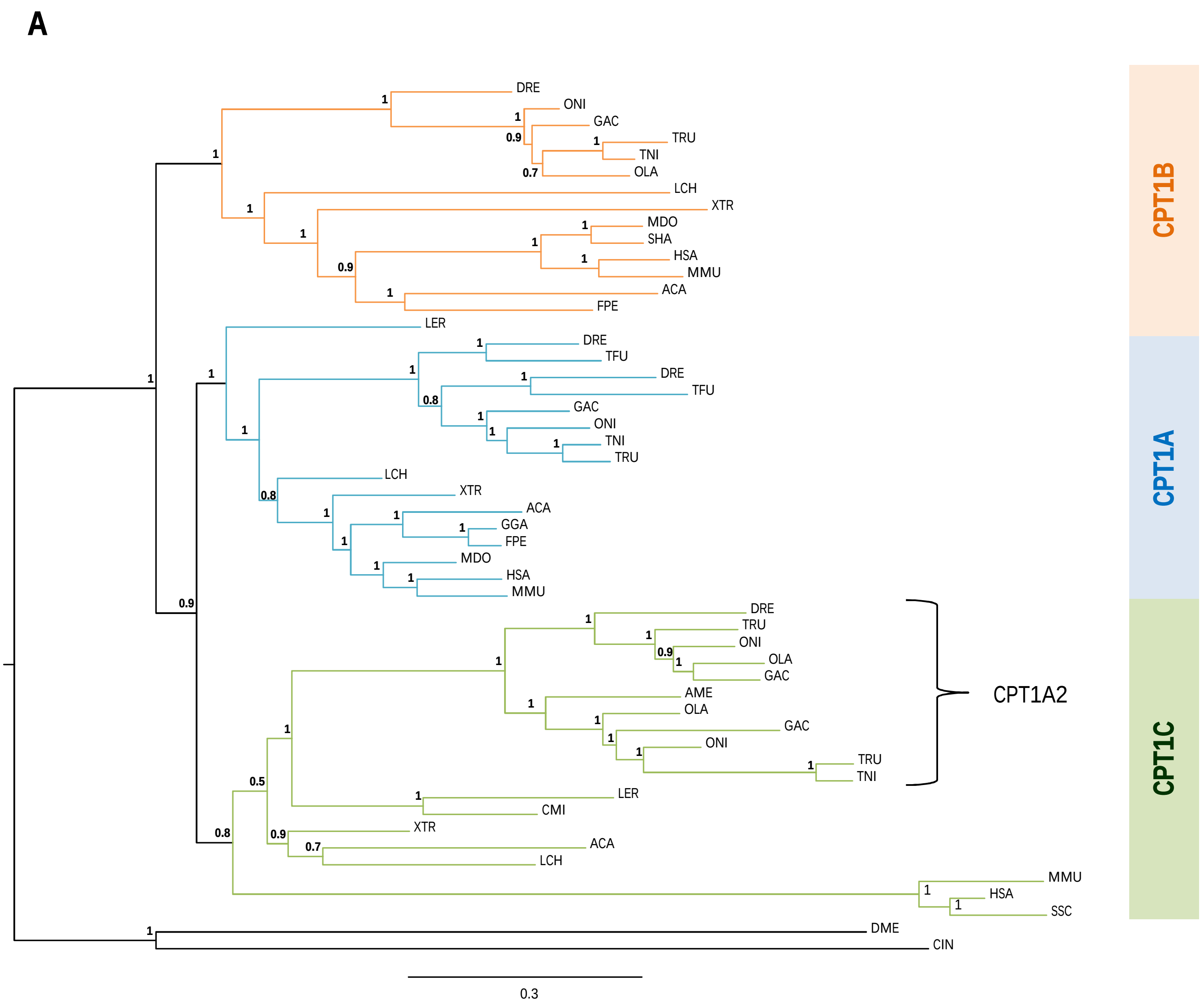

Phylogenetic analysis methods are the same as described in the main manuscript with the exception that sequence alignment input in PhyML contained gaps.

HSA – *H. sapiens*; SSC- *S. scrofa;* MMU – *M. musculus*; SHA – *S. harrisii*; MDO – *M. domestica*; ACA – *A. carolinensis* ; GGA – *G. gallus*; FPE – *F. peregrinus;* XTR – *X. tropicalis* ; LCH – *L. chalumnae* ; DRE – *D. rerio*; AME – *A. mexicanus;* TNI – *T. nigroviridis*. TFU – *T. fulvidraco;* GAC – *G. aculeatus* ; TRU – *T. rubripes* ; ONI – *O. niloticus* ; OLA – *O. latipes* ; CMI – *C. milii;* LER - *L. erinacea;* CIN – *C. instestinalis* ; DME – *D. melanogaster*

B

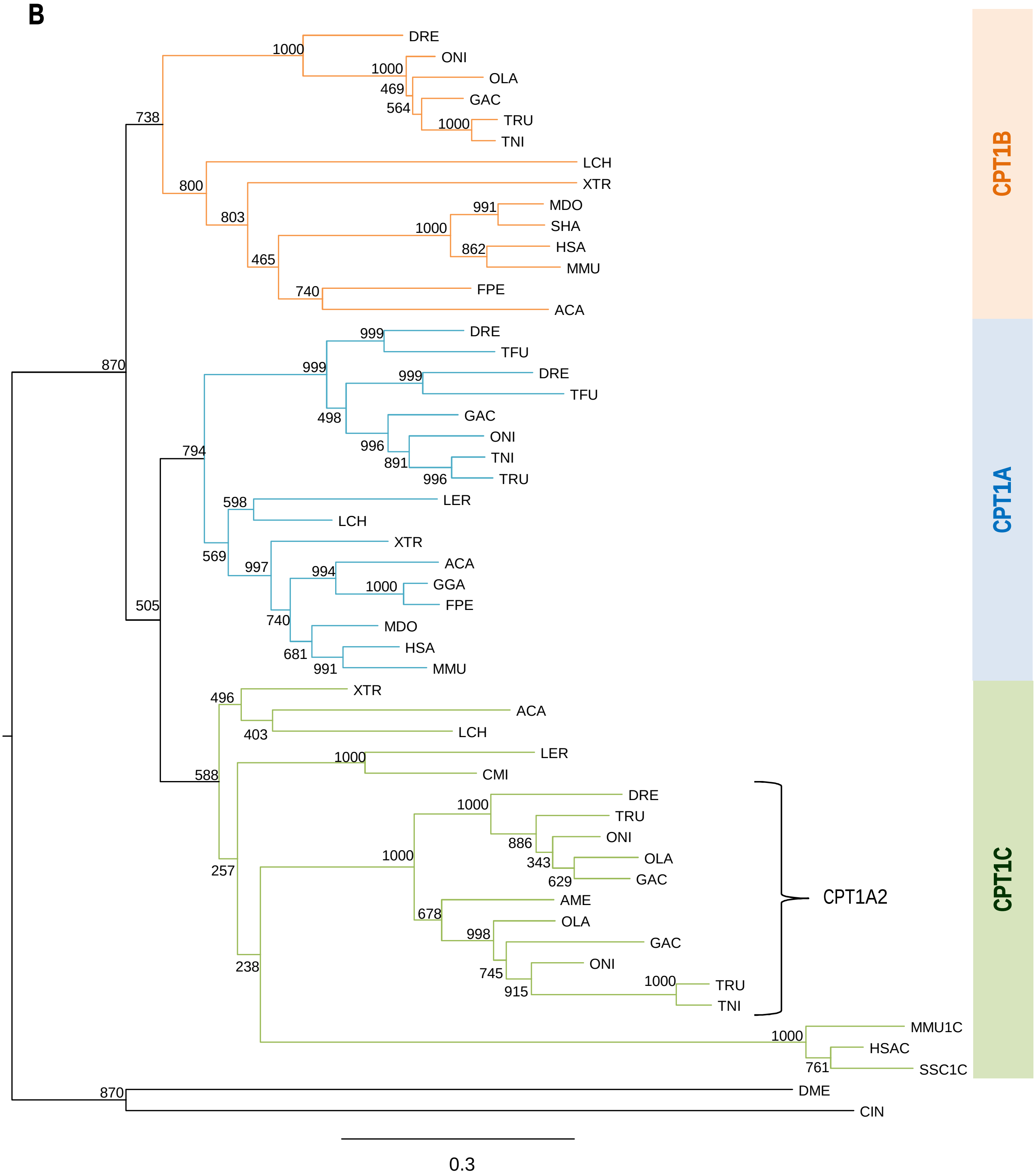

Maximum likelihood phylogenetic analysis was performed in PhyML v3.0 server. using the protein evolutionary model LG +G +F (previously calculated in Protest) and the number of bootstrap replicates was set to 1000. The resulting tree was visualized in Fig Tree V1.3.1 avaliable at http://tree.bio.ed.ac.uk/software/figtree/ and rooted DME and CIN

HSA – *H. sapiens*; SSC- *S. scrofa;* MMU – *M. musculus*; SHA – *S. harrisii*; MDO – *M. domestica*; ACA – *A. carolinensis* ; GGA – *G. gallus*; FPE – *F. peregrinus;* XTR – *X. tropicalis* ; LCH – *L. chalumnae* ; DRE – *D. rerio*; AME – *A. mexicanus;* TNI – *T. nigroviridis*. TFU – *T. fulvidraco;* GAC – *G. aculeatus* ; TRU – *T. rubripes* ; ONI – *O. niloticus* ; OLA – *O. latipes* ; CMI – *C. milii;* LER - *L. erinacea;* CIN – *C. instestinalis* ; DME – *D. melanogaster*

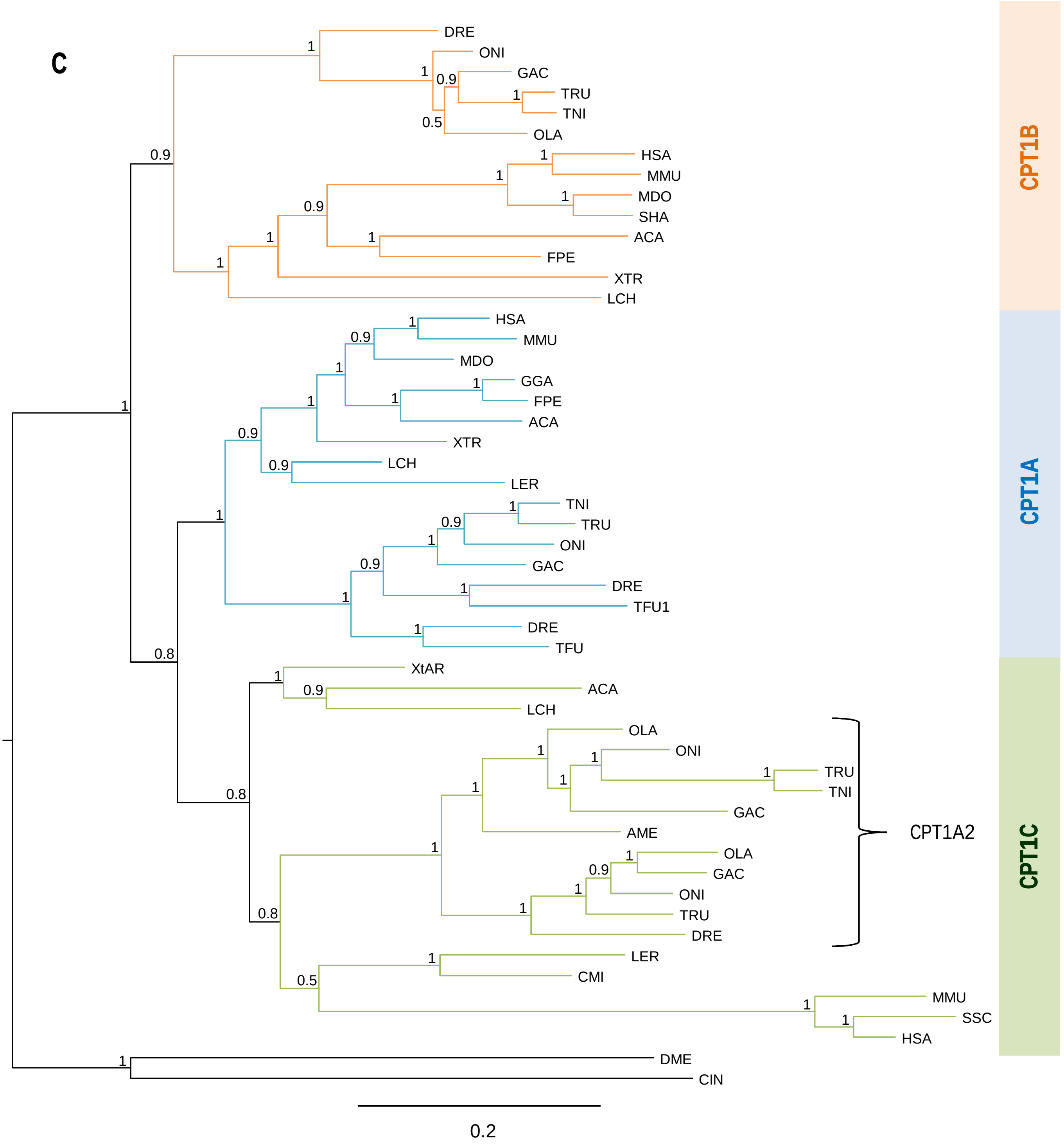

Bayesian phylogenetic analysis was performed using MrBayes v3.2.3 available in CIPRES Science Gateway V3.3. MrBayes was run for 1 million generations with the following parameters: rate matrix for aa=mixed, nruns=2, nchains=4, temp=0.2, sampling set to 1000 and burin to 0.25. The resulting tree was visualized in Fig Tree V1.3.1 avaliable at http://tree.bio.ed.ac.uk/software/figtree/ and rooted DME and CIN.

HSA – *H. sapiens*; SSC- *S. scrofa;* MMU – *M. musculus*; SHA – *S. harrisii*; MDO – *M. domestica*; ACA – *A. carolinensis* ; GGA – *G. gallus*; FPE – *F. peregrinus;* XTR – *X. tropicalis* ; LCH – *L. chalumnae* ; DRE – *D. rerio*; AME – *A. mexicanus;* TNI – *T. nigroviridis*. TFU – *T. fulvidraco;* GAC – *G. aculeatus* ; TRU – *T. rubripes* ; ONI – *O. niloticus* ; OLA – *O. latipes* ; CMI – *C. milii;* LER - *L. erinacea;* CIN – *C. instestinalis* ; DME – *D. melanogaster*

D

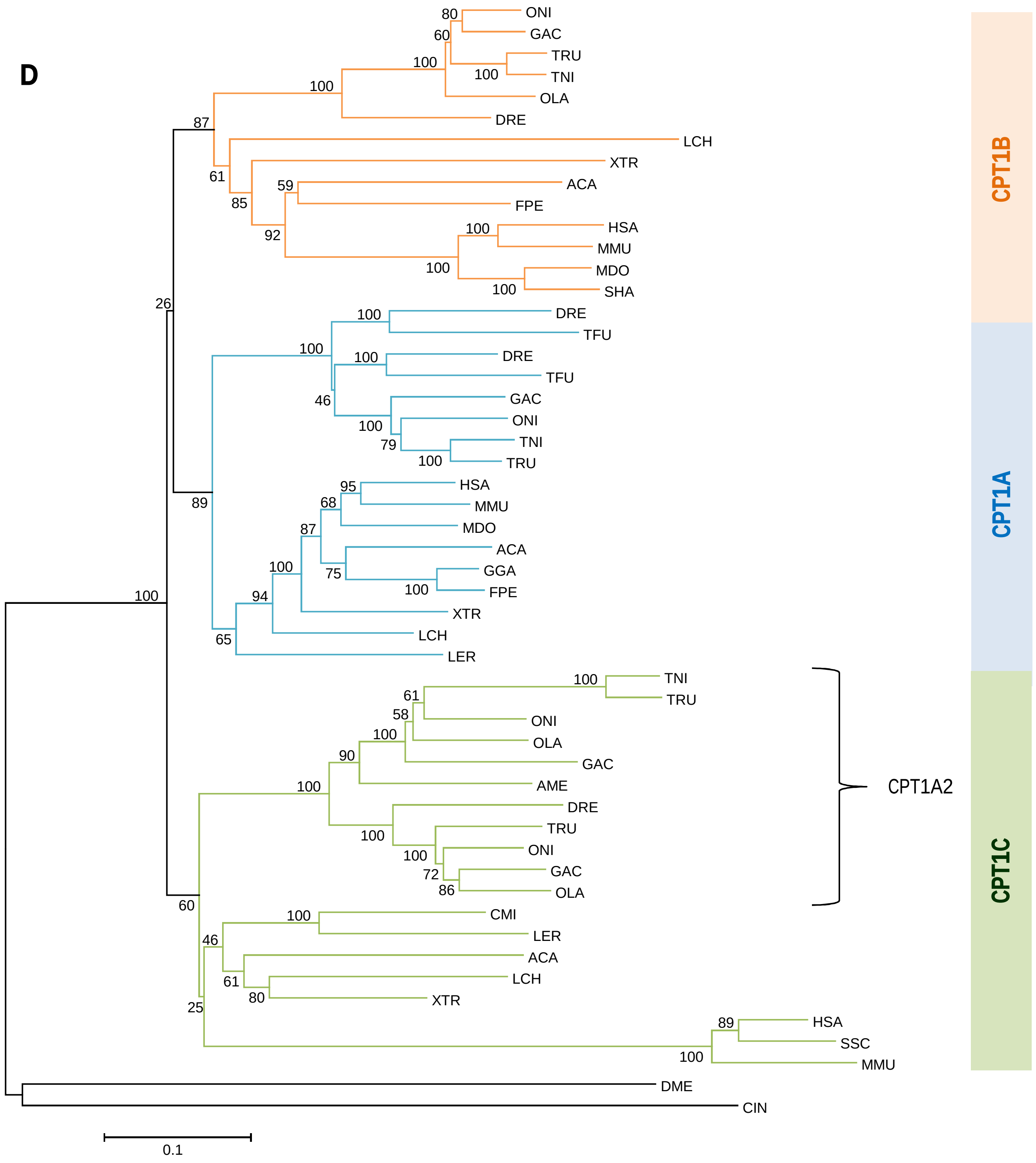

Phylogenetic analysis conducted in MEGA6 using Neighbor-Joining method . The percentage of replicate trees in which the associated taxa clustered together in the bootstrap test (100 replicates) are shown next to the branches. The evolutionary distances were computed using the JTT matrix-based method and are in the units of the number of amino acid substitutions per site.

HSA – *H. sapiens*; SSC- *S. scrofa;* MMU – *M. musculus*; SHA – *S. harrisii*; MDO – *M. domestica*; ACA – *A. carolinensis* ; GGA – *G. gallus*; FPE – *F. peregrinus;* XTR – *X. tropicalis* ; LCH – *L. chalumnae* ; DRE – *D. rerio*; AME – *A. mexicanus;* TNI – *T. nigroviridis*. TFU – *T. fulvidraco;* GAC – *G. aculeatus* ; TRU – *T. rubripes* ; ONI – *O. niloticus* ; OLA – *O. latipes* ; CMI – *C. milii;* LER - *L. erinacea;* CIN – *C. instestinalis* ; DME – *D. melanogaster*
