# Supplementary material for: The Origin and Diversity of Cpt1 Genes in Vertebrate Species
Source: PLoS One. 2015 Sep 30;10(9):e0138447. doi: 10.1371/journal.pone.0138447 (PMC4589379; doi:10.1371/journal.pone.0138447)
Supplement: S6 Fig — (PDF) [file pone.0138447.s006.pdf]

Supporting information 6 Fig: Supporting phylogenetic analysis of *Cpt1c* neighboring genes *Dnaaf3* and *Tnnt1*.

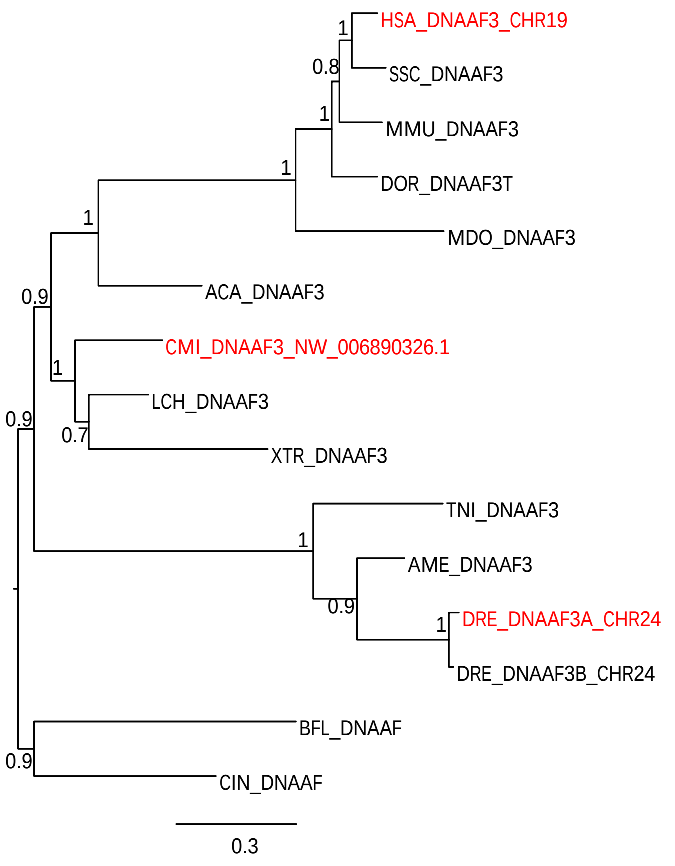

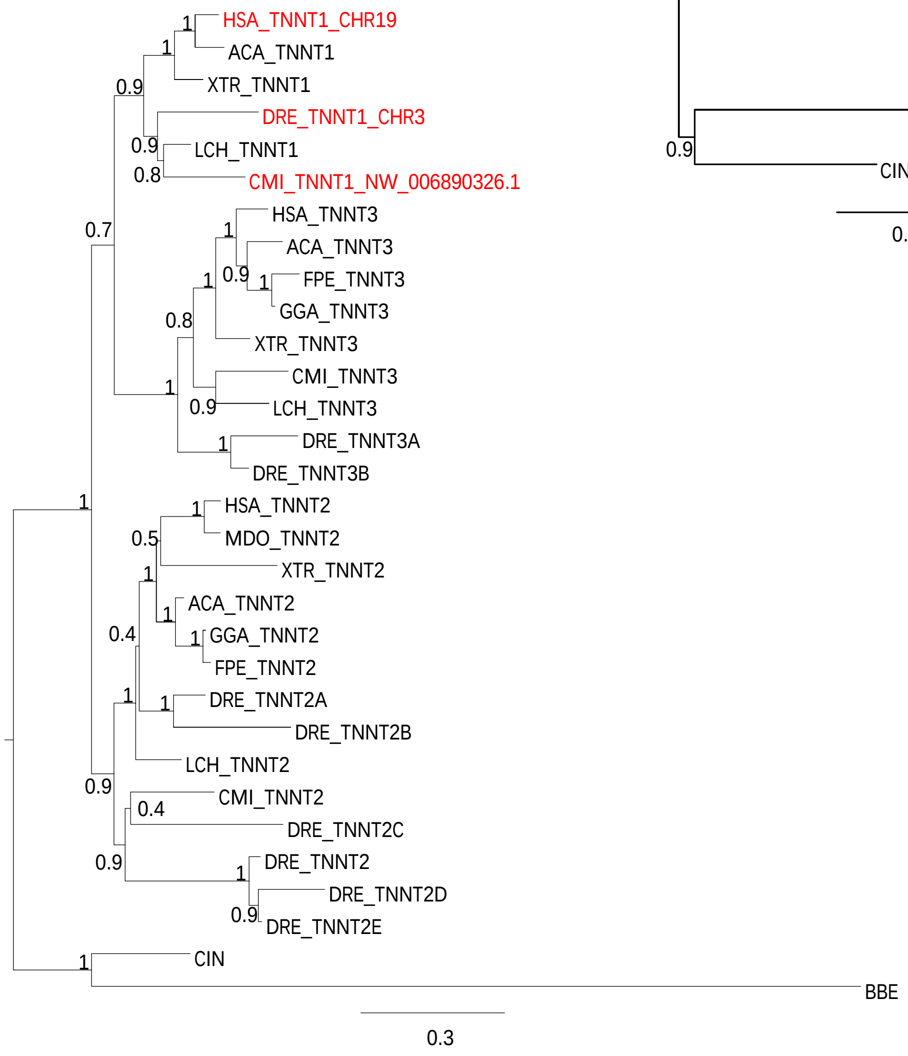

Sequence alignment was performed using MAFFT with the L-INS-i method (14). The final alignment was curated in BioEdit version 7.2.5 (15) with the removal of all columns containing gaps. Molecular phylogenetic analysis by Maximum Likelihood was performed in PhyML with SMS (smart model selection) option, node values represent branch support using the aBayes algorithm. Genes neighbouring *Cpt1c* are depicted in red, with the corresponding chromosomal location.

HSA – *H. sapiens*; SSC- *S. scrofa;* MMU – *M. musculus*; MDO – *M. domestica*; DOR – *D. ordii;* ACA – *A. carolinensis* ; GGA – *G. gallus*; FPE – *F. peregrinus;* XTR – *X. tropicalis* ; LCH – *L. chalumnae* ; CMI – *C. milii* ; DRE – *D. rerio*; AME – *A. mexicanus; TNI – T. nigroviridis*, CMI – *C. milii,*, CIN – *C. instestinalis;* BBE – *B. belcheri;* BFL – *B. floridae*
