# Supplementary material for: The Origin and Diversity of Cpt1 Genes in Vertebrate Species
Source: PLoS One. 2015 Sep 30;10(9):e0138447. doi: 10.1371/journal.pone.0138447 (PMC4589379; doi:10.1371/journal.pone.0138447)
Supplement: S1 Table — (PDF) [file pone.0138447.s007.pdf]

Supporting information 1 Table: List of sequences used for the molecular phylogenetic analysis and respective accession numbers (GenBank or Ensembl).

Species	Gene	Accession number
A. carolinensis	CPT1A	XP_003214835.1
	CPTC	XP_003222743.1
	CPT1B	ENSACAP00000001055
C. intestinalis	CPT1	ENSCINP00000007072
C. milii	CPT1C	AGD98733.1
D. melanogaster	CPT1	CAB52415.1
D. rerio	CPT1Aa	XP_002666893.2
	CPT1Ab	XP_005166530.1
	CPT1Ca	XP_005164116.1
	CPT1Cb	XP_002666747.2
	CPT1B	XP_005159068.1
F. peregrinus	CPT1A	XP_005236351.1
	CPT1B	XP_005243516.1
G. aculeatus	CPT1A	ENSGACP00000014767
	CPT1Ca	ENSGACP00000010584
	CPT1Cb	ENSGACP00000008742
	CPT1B	ENSGACP00000016316
G. gallus	CPT1A	NP_001012916.1
H. sapiens	CPT1A	NP_001867.2
	CPT1B	NP_689451.1
	CPT1C	NP_001186681.1
L. chalumnae	CPT1A	ENSLACP00000021346
	CPT1C	ENSLACP00000014661
	CPT1B	ENSLACP00000008053
L. erinacea	CPT1A	KF570112
	CPT1C	KF570111

M. domestica	CPT1A	XP_001363149.1
	CPT1B	XP_001366412.1
M. musculus	CPT1A	NP_038523.2
	CPT1B	NP_034078.2
	CPT1C	NP_710146.1
O. latipes	CPT1Ca	XP_004080367.1
	CPT1Cb	XP_004071913.1
	CPT1B	XP_005470933.1
O. niloticus	CPT1A	XP_003440402.1
	CPT1Ca	XP_003438524.2
	CPT1Cb	XP_003446513.1
	CPT1B	XP_005470933.1
T. nigroviridis	CPT1A	CAG01138.1
	CPT1C	CAG07569.1
	CPT1B	CAG11364.1
X. tropicalis	CPT1A	XP_004913492.1
	CPT1C	NP_001107300.1
	CPT1B	NP_001072766.1
S. scrofa	CPT1C	XP_005664799.1
T. rubripes	CPT1A	XP_003967444.1
	CPT1Ca	XP_003961459.1
	CPT1Cb	XP_003964428.1
	CPT1B	XP_003967147.1
T. fulvidraco	CPT1Aa	AFO11024.1
	CPT1Ab	AFO11025.1
